# Protein supersaturation powers innate immune signaling

**DOI:** 10.1101/2023.03.20.533581

**Published:** 2023-03-21

**Authors:** Alejandro Rodriguez Gama, Tayla Miller, Jeffrey J. Lange, Jianzheng Wu, Xiaoqing Song, Shriram Venkatesan, Jay R. Unruh, Dan Bradford, Randal Halfmann

**Affiliations:** 1. Stowers Institute for Medical Research, Kansas City, MO; 2. Department of Biochemistry and Molecular Biology, University of Kansas Medical Center, Kansas City, KS, USA

## Abstract

The response of cells to existential threats such as virus invasion often involves semi-crystalline polymerization of certain signaling proteins, but the highly ordered nature of the polymers has no known function. We hypothesized that the undiscovered function is kinetic in nature, emerging from the nucleation barrier to the underlying phase transition, rather than the material polymers themselves. We explored this idea using fluorescence microscopy and Distributed Amphifluoric FRET (DAmFRET) to characterize the phase behavior of all 116 members of the death fold domain (DFD) superfamily, the largest group of putative polymer modules in human immune signaling. A subset of them polymerized in a nucleation-limited manner able to digitize cell state. These were enriched for the highly connected hubs of the DFD protein-protein interaction network. Full-length (F.L) signalosome adaptors retained this activity. We then designed and carried out a comprehensive nucleating interaction screen to map the pathways of signaling through the network. The results recapitulated known signaling pathways including a recently discovered link between the different cell death subroutines of pyroptosis and extrinsic apoptosis. We went on to validate this nucleating interaction *in vivo*. In the process, we discovered that the inflammasome is powered by constitutive supersaturation of the adaptor protein, ASC, implying that innate immune cells are thermodynamically fated for inflammatory cell death. Finally, we showed that supersaturation in the extrinsic apoptosis pathway commits cells to die, whereas the lack of supersaturation in the intrinsic apoptosis pathway permits cells to recover. Our results collectively suggest that innate immunity comes at the cost of occasional spontaneous cell death, and uncover a physical basis for the progressive nature of age-associated inflammation.

Cells of the immune system respond rapidly and decisively to perceived threats via the formation of large protein complexes known as signalosomes. Signalosomes transduce minute signals to major outputs such as programmed cell death (1–4)).

Signalosome formation begins when a threat receptor protein oligomerizes upon binding a pathogen- or damage-associated molecular pattern. These oligomers then either directly or indirectly activate effector proteins such as transcriptional regulators and pro-caspases. In many signalosomes, receptors and effectors are bridged by one or more adaptor proteins. The underlying protein-protein interactions typically involve members of the death fold (DFD, comprising the death domain [DD], death effector domain [DED], caspase activation and recruitment domain [CARD], and pyrin domain [PYD] subfamilies), Toll/Interleukin-1 Receptor (TIR), and/or RIP homotypic interaction motif (RHIM) families (4–6).

Some signalosomes exhibit an unusual biochemical activity that, ironically, is otherwise associated with pathogenic infectious proteins known as prions (2, 7–18). Specifically, representatives of each of the DFD, TIR, and RHIM families have been found to amplify signaling by catalyzing their own activation via self-templated supramolecular polymerization. The initial formation, or “nucleation”, of a prion polymer involves a rare fluctuation in protein conformation and density. In the cases of pathogenic prions, that fluctuation tends to occur so infrequently that the proteins persist in their soluble forms —sometimes for years— even though the polymerized state is thermodynamically favored. Soluble states that persist despite a thermodynamic drive towards a solid phase are said to be supersaturated.

The ability of innate immune signaling proteins to form prion-like polymers appears to precede the emergence of complex life (19, 20). Its apparently fundamental role is nevertheless unclear. Proposed functions include signal amplification, scaffolding, and cooperativity (4, 6, 21). None of these completely withstand scrutiny, however (5). While signal amplification is clearly important for allowing the immune system to mount decisive cellular responses, signalosomes are poorly suited to this function because solid state deposition limits the otherwise exponential activation that would occur if the proteins remained in solution. Indeed, soluble positive feedback mechanisms abound in biology (22), and the autocatalytic potential of pro-caspase activation in particular, would seem to obviate the prion-like mechanism for amplification. Hypothetical scaffolding functions can be largely dismissed by the fact that arbitrarily condensing or even dimerizing effectors suffices for their activation, as has been well-established for apical pro-caspases (23, 24) and effector kinases (25, 26), suggesting that the ordered structures of the polymers are materially irrelevant for activation. Indeed, even MAVS, a DFD protein with well-characterized prion-like behavior (13, 27), failed to form detectable polymers when stimulated endogenously (28). Finally, a cooperativity function is unsatisfactory given that other forms of assembly are better suited. Polymers have a finite nucleus size and, therefore finite cooperativity (29), no different than other quaternary states of proteins. Liquid liquid phase separation (LLPS) in contrast approaches infinite cooperativity near the phase boundary (30). It also has relatively few sequence constraints and is therefore highly accessible from an evolutionary perspective (31). Why crystalline ordering should be central to innate immune signaling therefore remains unresolved.

Our recent investigation of the CBM signalosome, which regulates proinflammatory transcription factor NF-kB activation, suggests a plausible function for prion-like polymerization. We showed that the high entropic cost of the underlying transition in both density and ordering (5, 32, 33) creates a nucleation barrier large enough for the DFD adaptor protein, BCL10, to exist in a constitutively supersaturated state that stores energy for subsequent switch-like and decisive activation (24). The more phase separation is coupled to conformational ordering, the more nucleation is rate-limited by intramolecular fluctuations. This allows for smaller nuclei, and therefore, theoretically, increased sensitivity to pathogen-associated molecular patterns. Indeed, immune signaling pathways necessarily have extraordinary sensitivity —perhaps enough to detect single viral genomic RNAs (34)— in order to defeat enemies that will otherwise rapidly and catastrophically self-replicate. While this idea remains to be tested in the context of immune signaling, we have shown for a pathologic polymer that nucleation via an intramolecular fluctuation within a single polypeptide molecule can trigger a cell-wide change in protein activity (Kandola et al.).

These considerations lead us to hypothesize that innate immune signaling is commonly regulated by functional nucleation barriers arising from disorder-to-order transitions encoded in the underlying proteins’ sequences.

Our hypothesis predicts a certain irreversibility of DFD signaling that lends it to cell fate decisions such as immune cell differentiation and programmed cell death. This irreversibility arises from the fact that, once nucleated, polymers act as a sink for newly synthesized protein that prevents the cell from returning to the naive, supersaturated state. Unless the DFD polymers activate a biological mechanism for their own dissolution or expulsion by the cell, the consequences of their nucleation will tend to be permanent. Conversely, we would predict that DFDs associated with reversible cell states —such as transient activation of the mitochondrial apoptosis pathway during development (35, 36)— will not be supersaturated.

The highly ordered nature of DFD polymers facilitates signaling specificity that allows orthogonal DFD pathways to co-exist. DFDs in the same pathway tend to belong to the same DFD subfamily (37) and to make functionally important quasi-homotypic interactions, for example nucleating each other’s polymerization. These interactions variously regulate the responsiveness of innate immunity and hypothetically create redundancies that ensure cells die in the face of pathogens ever-evolving to circumvent individual pathways. Indeed, the now widely observed flexibility of cell death pathways has fueled an emerging perspective that they all function within a single, coordinated cell death system (38–40). The extent to which specificity and crosstalk are governed by DFD structure as opposed to the broader protein and cellular context has not been studied systematically. Doing so will be necessary to fully understand how cells make life and death decisions.

With these questions in mind, we systematically assessed the intrinsic capacity of every human DFD to 1) supersaturate in living cells, and 2) template the nucleation of other DFDs. We achieved this goal using a combination of high throughput fluorescence microscopy and a powerful cytometry method, Distributed Amphifluoric FRET (DAmFRET) (32, 41), to evaluate sequence-encoded nucleation barriers to self-assembly in the fully orthogonal biological context of budding yeast cells. This effort uncovered 18 protein modules with prion-like behavior, and further identified DFD interactions that regulate their nucleation. The resulting networks largely recapitulated known signalosomes, with prion-like executive function concentrated in the highly connected hubs of the network. We further mapped signaling through both DFDs of the major inflammasome hub —ASC/PYCARD. Then, using custom optogenetic tools to manipulate endogenous signaling, we confirmed an inter-signalosome nucleating interaction linking two major modes of cell death, while showing that ASC is thermodynamically precommitted to activate. Finally, we tested the hypothesis that nucleation-limited polymerization governs the reversibility of cell death signaling by creating domain-swapped chimeras of intrinsic apoptosis signaling proteins, and found that prion-like DFDs indeed confer irreversibility. Collectively, our work uncovers a fundamental executive function of nucleation barriers in innate immunity.

## A subset of DFDs are supersaturable

We used a sequence- and literature-guided search to identify 116 DFDs across 104 human proteins (Table S1 and Figure S1). Most of these are multidomain proteins with a single DFD at either terminus, a location that allows maximum accessibility for protein-protein interactions. Twelve proteins contained two DFDs. These were arranged in tandem —often as a single structural unit— with the exceptions of TNFRSF21 and NLRP1. We therefore included in our analyses the ten tandem DFDs in addition to all DFDs in isolation.

We sought to characterize the intrinsic phase behavior (sequence-encoded phase boundaries and kinetic barriers) of each DFD, i.e. in the absence of other DFDs or regulatory interactions but under otherwise physiological conditions. For this purpose we used the budding yeast, *Saccharomyces cerevisiae*, as an orthogonal eukaryotic cell that completely lacks DFDs and their specific regulatory factors. We subcloned all individual and tandem DFDs for strong inducible expression as fusions to the bright, monomeric fluorescent protein, mEos3.1. The fusion was made opposite the native N- or C-terminus where possible so as to minimize non-native steric effects.

**Figure 1:**
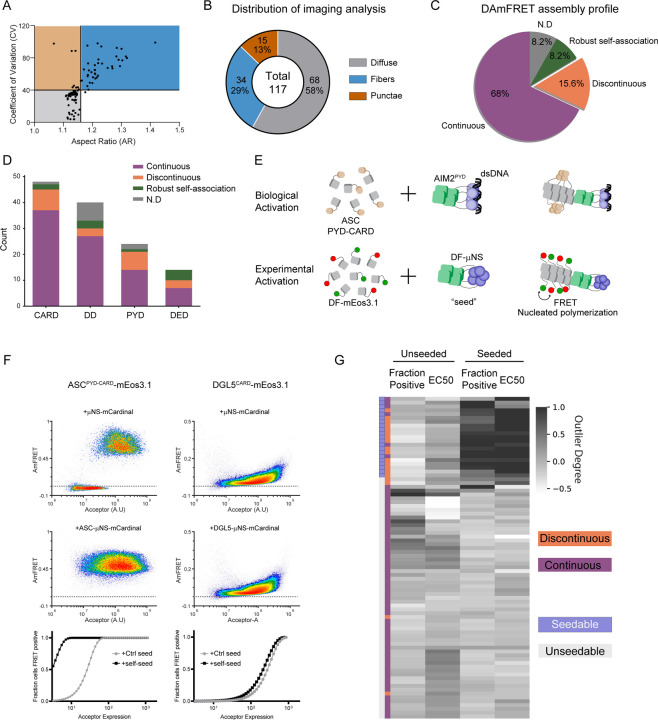
A subset of DFDs are supersaturable A. Characterization of protein localization based on image properties of DFDs in orthogonal yeast cells by high-throughput confocal microscopy and Fiji-based processing. B. Classification of the proteins as entirely diffuse, partly fibrous or punctate based on boundaries on the scatter plot of coefficient of variation vs aspect ratio depicted in A. C. Classification of the proteins based on the distribution of self-assembly (AmFRET) across a wide range of expression, in a population of yeast cells using Distributed Amphifluoric FRET (DAmFRET). D. Distribution of protein classifications from DAmFRET across the subfamilies of DFD superfamily. E. Schematic depicting our experimental design to probe the ability of a DFD to be seeded. Fusion of the same DFD with a multivalent viral condensate-forming protein, μNS, in trans was to mimic biological activation of a DFD protein that is supersaturated. F. Representative DAmFRET analysis of a supersaturated DFD that could be seeded (left) and of another DFD that expressed comparatively well but could not be seeded and hence potentially not supersaturable. Note that the supersaturated protein exhibits a discontinuous distribution of AmFRET across the expression range. G. A heat map of DAmFRET parameters (fraction of high-AmFRET containing cells, and EC50 - protein expression level at which 50% of cells contain high-AmFRET) that would change drastically upon getting seeded by μNS-fusion in trans, reveals that proteins that show a clear difference in both the parameters, in the presence of the seed are those that exhibit discontinuous DAmFRET profiles.

We then used DAmFRET to analyze the self-association of each protein as a function of its intracellular concentration following twenty hours of induction. The data revealed a diversity of behaviors by DFDs (Figure 1C, 1D and S2A), ranging from no self-association at any concentration to robust self-association even at the lowest concentrations. For many of the DFDs, cells partitioned between a low FRET population and high FRET population. In some cases (discovered further below), the cells appeared to do so rather sharply beyond a specific concentration, as evidenced by a prominent kink in the DAmFRET plot. In seventeen cases the cells acquired FRET discontinuously, switching semi-stochastically to a discrete high FRET state over a range of concentrations. We previously showed that discontinuous transitions result from large sequence-encoded nucleation barriers that are characteristic for prion-like self-assembly, whereas continuous transitions instead reflect phase separation through dynamic low-affinity interactions (24, 32, 41, 42).

Ordered self-assemblies tend to deviate from sphericity, and some DFDs have been found to polymerize into long filaments in the cytoplasm (14, 24, 43). We therefore assessed the subcellular distribution of each protein using high-throughput confocal microscopy. Just over half (63) of the DFDs expressed with a uniformly diffuse distribution in essentially all cells. The others localized to discrete structures in a fraction of cells. Of the 55 DFDs that had populated a high FRET state, 53 formed detectable assemblies in cells (Figure S2B). By measuring the aspect-ratios and coefficients of variation in fluorescence, these were further classified as punctate (n = 17) or fibrillar (n = 36) (Figure 1A and B). Each of the DFD subfamilies had multiple members in each category. Of the DFDs that populated a FRET-positive state, the filamentous DFDs were strongly biased towards those with discontinuous DAmFRET profiles (Figure S2C, chi-square contingency test, p<0.0001). All of the eight DFDs with diffuse fluorescence despite forming FRET-positive species lacked a nucleation barrier, suggesting their assembly is limited finite oligomers or emulsions (44–48).

As a direct test for nucleation barriers, we next introduced artificial genetically-encoded “seeds” for each DFD. We designed these seeds to generically mimic the oligomerized states of full length DFD proteins when they are naturally activated by pathogen-associated multivalent ligands, by genetically fusing each DFD to an inert and well-characterized homomultimeric domain, μNS (49). Provided that multimerization stabilizes the polymer conformation relative to potential off-pathway conformations, the resulting multimer will lower the nucleation barrier for any corresponding DFD expressed at supersaturating concentrations in trans, causing cells to switch from the low to the high FRET population (24) (Figure 1E). As expected, co-expressed seeds caused cells to switch to the high FRET state for all DFDs that had transitioned discontinuously in the absence of seeds (Figure 1F and G), but generally not those that had transitioned continuously (chi-square contingency test, p= 1.83e-09). Analyzing one such seeded example by fluorescence microscopy, we observed a corona of filaments radiating outward from the μNS punctum (Fig. S2D). We conclude that multiple DFDs exhibit sequence-encoded nucleation barriers that allow for their supersaturation and subsequent switch-like assembly in cells.

## A central role of supersaturation in signalosomes

**Figure 2:**
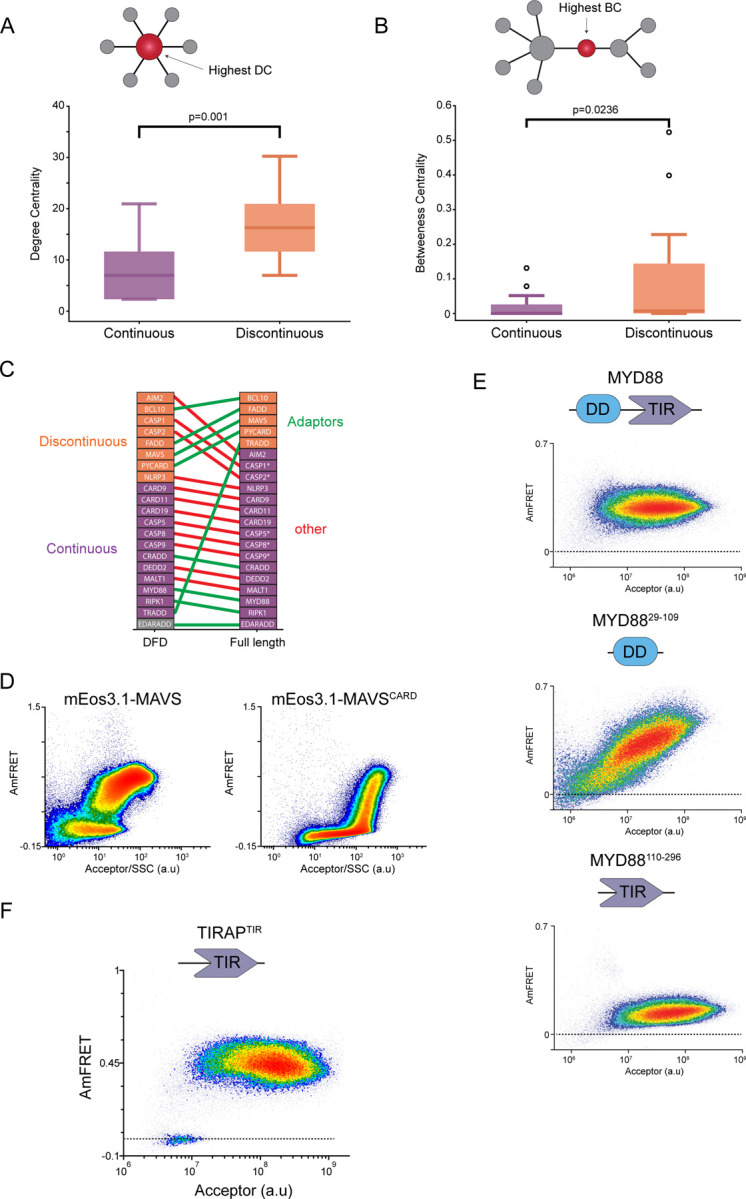
A central role for supersaturation in signalosomes A & B. Box whisker plot of the degree of centrality (A) and betweenness of centrality (B) revealing a significantly higher score in both parameters for DFD proteins that exhibit a discontinuous DAmFRET profile compared to those with a continuous profile. C. Visualization of how the DAmFRET profiles of isolated DFD domains (left) change in their full-length contexts (right) showing that only adaptor proteins tend to retain supersaturability (discontinuous profile) in their full-length context. Note that this list is only of proteins that contain only a single DFD. D. DAmFRET of MAVS full-length and CARD domain-only showing greater supersaturability of the full-length, revealed by a prominent discontinuous distribution of populations. E. DAmFRET panel intriguingly showing no detectable supersaturability of MYD88 or its individual DFD domains, despite its being an adaptor protein. F. Discontinuous DAmFRET profile of TIRAP which contains a DFD domain in TIR, functioning upstream of MYD88 to activate TLR signaling in response to pathogenic stimuli.

Is supersaturability important for signaling through the DFD network? To evaluate, we first used the STRING database (50) to construct the network of physically interacting DFD-containing proteins. We then calculated two fundamental centrality measures —degree and betweenness— for each DFD protein in the network. Central proteins tend to be essential (51), more abundant (52), slower evolving (53), and more frequently targeted by pathogens (52, 54) than noncentral proteins. Proteins with a high degree of centrality constitute hubs in protein-protein interaction networks, while proteins with high betweenness constitute bottlenecks that disproportionately control information flow through the network. We found that both measures were higher for discontinuous DFDs than for continuous DFDs (Figure 2A and B). This indicates that DFDs with a discontinuous profile are positioned for greater control over pathway activity and suggests an essential general function of supersaturation in innate immunity.

For supersaturation to function as hypothesized, the DFD must remain supersaturable in the context of its full-length protein. Any interactions with other parts of the protein that either lower the nucleation barrier or raise the solubility would reduce supersaturation and, therefore its control over the pathway. Multiple DFDs that are otherwise capable of polymerization have been shown to be prevented from doing so by autoinhibitory interactions. We, therefore, evaluated the phase behaviors of multiple FL proteins sampled from across the DFD protein network (in addition to those proteins screened above that consist of only a DFD: CARD18, PEA15, POP3, PYDC1, and PYDC2). Many of the proteins were comparably (in)soluble to their corresponding DFDs (Figure 2C and Figure S3). Others formed uniform moderate AmFRET consistent with autoinhibited oligomers, as we previously observed for FL CARD9 (15, 24). A few of them were, however, comparably or even more supersaturable than their corresponding discontinuous DFDs. Strikingly, all of these proteins function as adaptors: ASC, MAVS, BCL10, and FADD (Figure 2C).

Of the adaptor proteins found to be supersaturable, MAVS is particularly informative with respect to function. MAVS signaling is incredibly sensitive, potentially detecting as few as a single dsRNA in the cytoplasm (34, 34, 55, 56). The protein also localizes to the mitochondrial outer membrane, where its activation depends on mitochondrial membrane potential (57, 58). Because the mitochondrial targeting sequence is at the protein’s C-terminus, we had fused mEos to the N-terminus (directly to the CARD domain) to avoid disrupting mitochondrial localization. To determine if the difference between MAVS and MAVS^CARD^ is attributable to mEos having been fused to the opposite terminus of the CARD domain in the DFD-only construct, we next compared MAVS and MAVS^CARD^ with identical fusions (to the N-terminus). Remarkably, the low-to-high FRET transition for MAVS^CARD^ was now continuous, rendering the difference between MAVS and MAVS^CARD^ even greater than before (Figure 2D). Our findings suggest that mitochondrial localization increases the dependence of nucleation on a conformational fluctuation while lowering its dependence on a density fluctuation, which theoretically allows the antiviral response to be triggered by a smaller number of activated MAVS molecules. This otherwise exceptional impact of FL protein context on DFD nucleation therefore supports our hypothesis that nucleation barriers heighten the sensitivity of innate immune signaling pathways.

A minority of the FL adaptors lacked a detectable nucleation barrier, exhibiting a single population of cells consistent with the proteins either fully monomeric or fully multimerized. Two of these, EDARADD and CRADD, do not respond to pathogen-associated signals. RIPK1 functions in concert with two other DFD-containing adaptors that were discontinuous (FADD and TRADD). MYD88 is the only DFD-containing adaptor in its subnetwork. We further tested if shorter inductions times with lower expression levels would reveal a nucleation barrier for MYD88. They did not (not shown). These findings are consistent with evidence that MYD88 exists as an autoinhibited oligomer in cells (59–61). MYD88 functions to link the activation of TIR-containing Toll-like receptors (TLRs) to DFD-containing effector proteins(62), and accordingly is the only adaptor with both a DFD and a TIR module (Figure 2E). Interestingly, TLR signaling in response to natural low-dose PAMPs requires a TIR-only adaptor TIRAP/MAL, upstream of MYD88 (Bonham et al., 2014, Horng et al., 2002), and this protein forms self-assembling filaments *in vitro*, via its TIR domain (9). We, therefore, reasoned that TIRAP rather than MYD88 may provide a functional nucleation barrier for TLR signaling. To explore this hypothesis in an unbiased manner, we tested representative TIR domains from multiple TIR-containing subpathways —TLR2, TLR3, TLR4, TLR5, SIGIRR, IL1RL2, IL1RAP, PIK3AP1, BANK1, SARM1, TICAM1, TICAM2, and TIRAP, as well as FL TIRAP ( Figure S4). Remarkably, TIRAP was the only member with an apparent nucleation barrier (Figure 2F). These data suggest that TLR signaling is kinetically controlled by adaptor TIR domain supersaturation analogous to DFD signalosomes.

The degree of supersaturation is defined as the natural logarithm of the ratio of total protein concentration to saturating concentration (63). If supersaturation is functional, one can expect evolution to have driven the numerator and denominator in opposite directions. Our hypothesis, therefore, predicts that proteins with discontinuous DFDs proteins will have lower saturating concentrations and greater endogenous concentrations. To obtain a proxy for the former, we fit the self-seeded DAmFRET data to a Weibull function to determine the concentrations at which 50% of cells acquired FRET (Table S1). We next extracted each protein’s physiological expression level from the Protein Abundance Database (64). We found that discontinuous DFDs tend to have lower saturating concentrations (Figure S5A-B) and to be expressed more abundantly (Figure S5C) than continuous DFDs. Moreover, these two values are indeed anticorrelated (Figure S5D), confirming that discontinuous DFDs are more likely to be supersaturated in their endogenous physiological contexts.

## The nucleating interactome

Multiple DFD subnetworks have been found to propagate signaling via nucleating interactions between different DFDs. To identify such interactions systematically, we mated our library of seed-expressing yeast strains with our library of mEos-expressing DAmFRET strains (Figure S6A). We then analyzed all ~20,000 arrayed diploid yeast strains by DAmFRET. After excluding files with fewer than 2500 cells, we fit the fraction of FRET-positive cells as a function of concentration to a Weibull function. We then used outlier selection based on the resulting EC50 and the total fraction of FRET-positive cells to identify nucleating interactions (Figure S6B and C).

In total, we identified 181 nucleating interactions (Figure S7), representing just 1.7% of the total library. Most of the interactions agree with the known specificity of the corresponding full length proteins and recapitulate known signalosomes, suggesting that signalosome assembly/composition is largely encoded by the structures of the DFDs themselves rather than the regulation and cellular context of their respective full length proteins. For example, the CARD of CASP1, the effector for the inflammasome, was nucleated exclusively by other CARDS in the inflammasome subnetwork.

**Figure 3:**
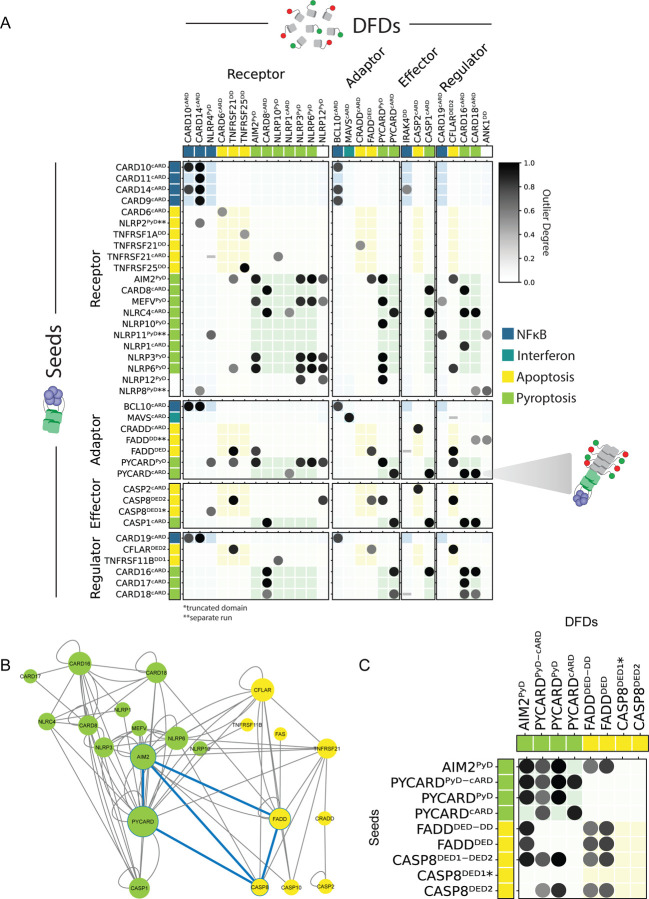
DAmFRET library screen reveals the nucleating interactome of DFDs A. Matrix visualization of DAmFRET data from the screen for μNS-fused DFDs in trans (down) that have positive effects on the nucleation of DFD-mEos3.1 constructs (across). Darker shading of the circle (higher Outlier Degree – see Methods) means a greater positive effect of DFD nucleation by the corresponding seed, termed nucleating interaction. Only proteins with detectable interactions are shown for brevity. Interactions among members of the same signaling pathway (in legend) appear in color shaded squares. B. Visualization of the extent of nucleating interactions by various DFDs reveals those with multiple interactions, including between apoptosis and pyroptosis (blue lines). Network was created in Cytoscape with node size corresponding to its degree of centrality. C. Matrix visualization (just as in panel A) for the DFD domains interacting across pyroptosis and apoptosis.

We observed multiple cross-seeding interactions between pyroptosis and extrinsic apoptosis DFDs (Figure 3B and C). For example, AIM2^PYD^, a receptor for the inflammasome, nucleated and/or was nucleated by Death-Inducing Signaling Complex members FADD^DED^, CASP10^DED-DED^, CASP8^DED-DED^, and CFLAR^DED-DED^. These heterotypic interactions are consistent with prior evidence for cross-talk between these two pathways(65, 66), while also suggesting that it is much more pervasive than previously realized.

Our results reveal two canonical signalosomes —the apoptosome and the PIDDosome, that lack supersaturable components. These are also atypical in their having a finite oligomeric rather than polymeric structure (67, 68), which emphasizes the dependence of nucleation barriers on semi-crystalline ordering. These two signalosomes are also exceptional from a functional perspective— they respond exclusively to cell-intrinsic signals of stress rather than extrinsic signals of infection (69, 70). Hence their functions do not require the extreme sensitivity that nucleation barriers hypothetically offer.

## Supersaturation commits cells to extrinsic apoptosis

We next explored the hypothetical role of supersaturation in endogenous innate immunity. We focused this effort on the inflammasome and extrinsic apoptosis signaling pathways, taking advantage of their strong cross-seeding interactions with the AIM2 receptor.

**Figure 4:**
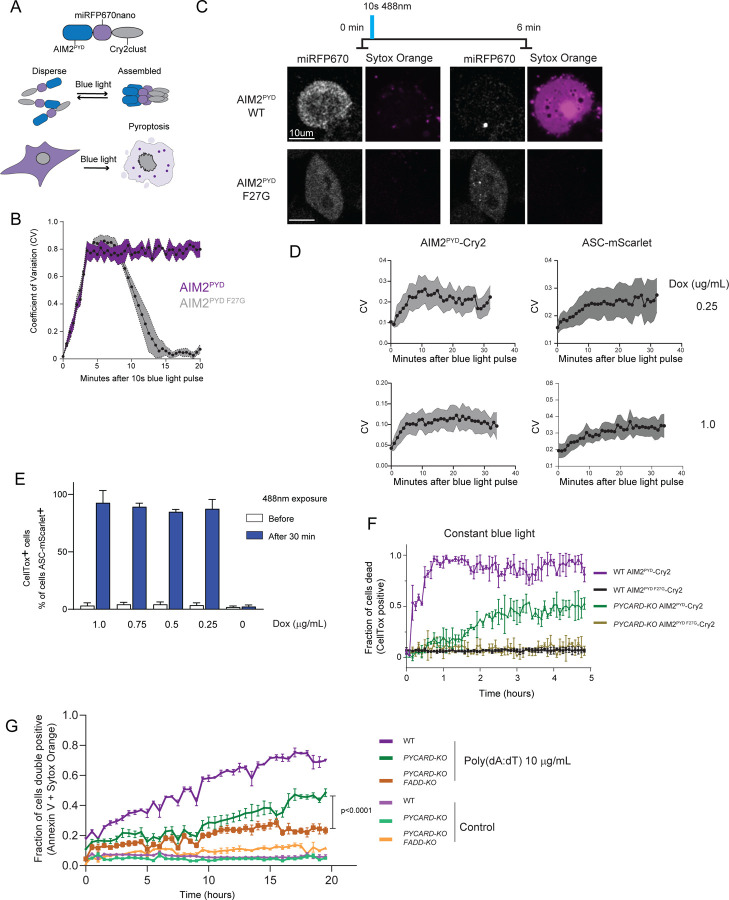
Supersaturation commits cells to extrinsic apoptosis A. Schematic of the experiment to transiently oligomerize the test DFD domain, AIM2 PYD, optogenetically and check the permanence of its self-assembly as well as a functional effect on cell death. B. Histogram of the measure of protein localization in HEK293T cells following 10 seconds of optogenetic activation shows WT AIM2 PYD rapidly switching localization from diffuse to punctate (high CV) and remaining stable long after the activation pulse, while F27G mutant oligomers induced by the blue light pulse disappear shortly after. C. Representative timelapse images from confocal microscopy of THP-1 monocytes showing the detection of self-assembly of WT AIM2 PYD but not a non-assembling point mutant F27G, minutes after a transient optogenetic oligomerization. Self-assembly of AIM2 PYD also results in cell death revealed by Sytox Orange staining. Scale bar D. Coefficient of variation (CV) analysis of AIM2 PYD-Cry2, directly oligomerized by the blue light pulse and of Doxycycline-inducible ASC-mScarlet not interacting with the optogenetic cluster directly, in engineered THP-1 cells. Plot reveals downstream activation of ASC self-assembly by AIM2 PYD polymers that remain stable long after the removal of the optogenetic pulse. Top and bottom panels reveal no distinct effect of ASC polymerization on ASC expression levels dictated by Doxycycline concentration. E. Quantification of CellTox staining of THP-1 cells 30 minutes after 10 seconds of optogenetic activation and in cells with varying Doxycycline (Dox) levels showing rapid cell death, a measure of pyroptosis, specifically following optogenetic pulse and irrespective of Dox concentration. F. Plot of fraction of CellTox-positive cells over time when cells were subjected to intermittent optogenetic pulse to oligomerize AIM2 PYD every 5 minutes, showing rapid cell death (violet trace) only when AIM2 PYD is WT and when ASC (PYCARD) is present. The absence of ASC results in slower kinetics, suggestive of apoptosis (green trace), while when AIM2 PYD is prevented from nucleating into stable self-assembly by point mutant version F27G, cell death virtually does not occur irrespective of the status of ASC (PYCARD) (black and golden traces). G. Quantification of apoptotic cell death of THP-1 cells by double staining of Sytox and Annexin V tracked over 20 hours, following exposure to AIM2 ligand, poly (dA:dT). p-value obtained from ANOVA followed by pair comparison.

To this end we developed an optogenetic tool to inducibly oligomerize AIM2^PYD^ independently of its cognate ligand, so that we could precisely control the strength of stimulation and measure the frequency of cell death through either pathway. We fused AIM2^PYD^ to the miRFP670nano fluorescent protein followed by Cry2clust (Figure 4A), a domain that reversibly oligomerizes in response to 488 nm blue light (71). To validate the protein, we first expressed it in HEK293T cells, which lack the downstream effectors of AIM2 that would otherwise trigger cell death upon its activation. We found that a 10-second pulse of blue light caused rapid and persistent clustering of the wild-type (WT) AIM2^PYD^ fusion protein. In contrast, AIM2^PYD^ F27G, harboring a mutation that disrupts a critical interface for polymerization, formed clusters that then dissociated within 15 minutes following the blue light pulse (Figure 4B), confirming that their persistence in the case of WT protein results from AIM2^PYD^ forming a polymer-like seed. This behavior is consistent with the self-assembly capacity of AIM2 observed by DAmFRET.

We then analyzed the downstream consequences of AIM2^PYD^ clustering in a human monocytic cell line, THP-1, with intact inflammasome and extrinsic apoptosis signaling pathways. In this context we expect that blue light exposure will trigger inflammasome assembly and cell death via pyroptosis, as assessed by Sytox Orange incorporation. AIM2^PYD^ remained diffuse in THP-1 cells before blue-light exposure but very rapidly formed clusters following a 10-second pulse of blue light. Sytox Orange accumulated in the cytoplasm and nucleus within two minutes of AIM2^PYD^ clustering. The rapid kinetics of cell death did not depend on the duration of the blue light pulse (Figure 4C). Cells expressing the polymerization defective mutant AIM2^PYD^ F27G failed to respond to blue light (Figure 4C).

We next directly investigated inflammasome assembly in response to blue light by knocking out *PYCARD*, the gene encoding ASC, in our AIM2^PYD^-Cry2clust THP-1 cells and then reconstituting it with ASC-mScarlet. We compared the expression level of the fusion protein to that of endogenous ASC in the original cell line to ensure that any response we may see is not an artifact of over-expression and confirmed that it expressed well below endogenous levels (Figure S8A-C). We then tracked AIM2^PYD^ and ASC localization following blue light exposure. In this experiment, we tracked pyroptosis by CellTox incorporation before and after 30 minutes of a 10-second blue light pulse. We found that both proteins clustered almost immediately after the pulse, and pyroptosis followed shortly thereafter (Figure 4D). The efficiency of ASC clustering and cell death did not depend on the expression levels of ASC within the range tested (Figure 4E). Given the rapidity of the response, the specificity of our stimulus, and the sub-endogenous expression level of ASC, these results argue that ASC is endogenously supersaturated even prior to stimulation.

To next investigate AIM2 induction of apoptosis, a slower form of programmed cell death, we incubated the cells in an incucyte with blue light stimulation every 5 minutes over a period of 5 hours. As expected, cells expressing endogenous ASC died rapidly ( Figure 4F). We then repeated the experiment in the *PYCARD-KO* cells, and again observed cell death, but this time with greatly delayed kinetics consistent with apoptosis. This form of cell death nevertheless depended on AIM2^PYD^ seed formation because the F27G mutation completely blocked CellTox staining (Figure 4F).

Next, we tested whether activation of endogenous AIM2 could induce cell death independent of ASC. We first observed that the AIM2 ligand, polydA:dT, robustly triggered cell death, albeit less abruptly than for light-induced AIM2^PYD^ clustering. By 18 hours, approximately 70% of WT THP-1 cells had become double positive for Sytox and AnnexinV-Alexa-488, a marker for dying or dead cells. Consistent with our previous findings, cell death was delayed for THP-1 *PYCARD*-KO cells (Figure 4G). Finally, we asked if this delayed form of cell death depended on FADD, the supersaturable adaptor for extrinsic apoptosis. Indeed, knocking out *FADD* (in addition to *PYCARD*) significantly reduced cell death (Figure 4G and S9). We suspect the residual cell death results from the ability of activated AIM2 to polymerize and directly engage CASP8, the effector of extrinsic apoptosis, analogously to FADD itself. Altogether, these data reveal that AIM2 forms persistent polymeric seeds following even transient stimulation, and these suffice to trigger apoptosis in the absence of pyroptosis. While we cannot conclude from these data alone that FADD is endogenously supersaturated, a compelling case can be made from previously published observations. Specifically, FADD self-association is required for extrinsic apoptosis (72, 73), increases linearly with time following stimulation, and proceeds to the extent that does not vary with the amount of receptor activation (74, 75). These properties collectively indicate that FADD activation is self-templating and ultimately governed by a pre-existing threshold that is presumably, the degree of supersaturation.

## Nucleation barriers underlie sensitive and irreversible cell death

Nucleation-limited polymerization releases potential energy that had been stored via supersaturation. This process will be irreversible in sufficiently closed systems, and therefore lends itself to cell death signaling, consistent with our findings for pyroptosis and extrinsic apoptosis. However, apoptosis can also be triggered intrinsically, via intracellular stresses that converge on the release of cytochrome C from mitochondria and subsequent apoptosome assembly. Remarkably, activation of intrinsic apoptosis is not necessarily irreversible, and in fact occurs transiently during development (35, 36). Our hypothesis predicts that the corresponding DFD proteins should therefore not be supersaturated. Indeed, our screen (Figures S2A and S3) found that neither the apoptosome nor its accessory signalosome, the PIDDosome (69, 70), have supersaturable components.

**Figure 5:**
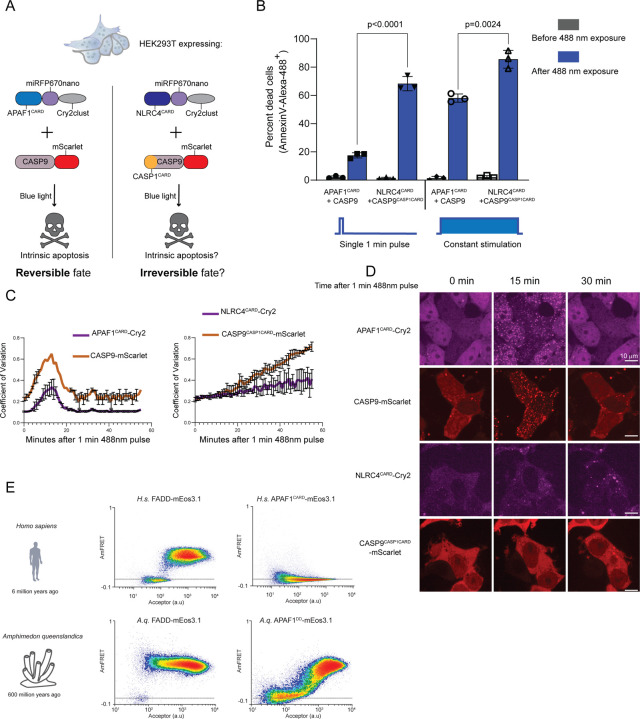
Nucleation barriers dictate irreversible cell death A. Schematic of experiment in HEK293T cells to probe the sensitivity of induction of cell death upon optogenetic stimulation of intrinsic apoptosis by either the typical APAF1 CARD and CASP9 pair or by the engineered NLRC4 CARD (in place of APAF1 CARD) and chimeric CASP9 with the CARD domain from CASP1 (CASP9^CASP1CARD^). While the former activates apoptosis from intrinsic triggers and is not supersaturable, the latter responds to extrinsic triggers in a normal context and is supersaturable. B. Quantification of cell death of the HEK293T chimeric cells (as in A) using AnnexinV-Alexa 488 staining, either upon a single 1 minute pulse of 488 nm laser followed by measurement after 2 hours (left half) or upon continuous excitation with 488 nm laser for the entire 2 hours before the measurement (right half). P-value derived from t-test analysis C. Coefficient of variation (CV) analysis of HEK293T cells expressing optogenetic constructs after a single 1 minute 488nm laser activation. Left, APAF1^CARD^-Cry2 and CASP9-mScarlet display rapid cluster formation that dissociates around 20 min. Right, A single pulse of 488nm laser activates cluster formation of NLRC4^CARD^-Cry2 and chimeric CASP9^CASP1CARD^. Such clusters continue to grow independent of any blue light source. D. Representative images from experiment in C. Clusters of APAF1^CARD^-Cry2 and CASP9-mScarlet do not self-sustain while NLRC4^CARD^-Cry2 and CASP9^CASP1CARD^ polymers continue to grow. E. DAmFRET analysis showing conservation of supersaturability of FADD from humans to model sponge, *Amphimedon queenslandica*, While the sponge APAF1DD showed a continuous DAmFRET profile compared to seemingly monomeric human homolog, it was nevertheless not supersaturated.

To test if the reversibility of intrinsic apoptosis activation results from its lack of nucleation barriers, we used HEK293T cells that lack the inflammasome sensor NLRC4, adaptor ASC, and effector CASP1. To allow for precise control of intrinsic apoptosis, we then co-transfected APAF1^CARD^ fused with miRFP670nano and Cry2clust, and CASP9 fused with mScarlet (Figure 5A). We then stimulated the cells with blue light for one minute and then monitored protein localization and cell death. We observed that APAF1^CARD^ and CASP9 colocalized to visible clusters in virtually all cells within 15 minutes (Figure 5C,D). These clusters then dissolved with similar kinetics over the course of the next ten minutes, confirming that neither of the proteins were supersaturated. A small fraction of the cells (< 20%) died within two hours ( Figure 5B).

CASP9 activation at the apoptosome can be detected within two minutes of assembly, and CASP3 cleavage within 5 minutes (76). Hence, it is very likely that a fraction of CASP9 had been activated in all cells prior to the dissolution of puncta, and this sufficed to kill some of the cells. To determine if prolonged stimulation increases cells’ commitment to death, we next stimulated for the entire two hours. This resulted in > 80% death. Together, these data suggest that apoptosome formation does not commit cells to death, consistent with expectations.

We therefore next asked if cells commit to death when the apoptosome is artificially supersaturated prior to stimulation. To do so, we cotransfected the supersaturable DFD, NLRC4^CARD^, and CASP9^CASP1 CARD^ (a CASP9 chimera with its own CARD replaced with that of CASP1, which is seeded by NLRC4^CARD^). After stimulating these cells with blue light for one minute, we observed that NLRC4^CARD^ formed clusters by 15 minutes, and CASP9^CASP1 CARD^ subsequently colocalized to these. Remarkably, rather than the clusters then disassembling as they had for the WT DFDs, they instead continued to grow at a constant rate for at least 50 minutes (Figure 5C,D), confirming that one or both proteins were supersaturated. Also unlike for the WT DFDs, most of these cells died by 2 hours even with just the one minute stimulation (Figure 5B). Continuous stimulation did not significantly increase the fraction of dead cells. We conclude that supersaturability governs the (ir)reversibility of cell death signaling.

Both the intrinsic and extrinsic pathways of apoptosis are conserved across metazoa (77–79). To determine if the differences in supersaturability between these two pathways is also conserved, we analyzed by DAmFRET the phase behavior of the APAF1^DD^ and FADD homologs from the model sponge, *Amphimedon queenslandica*. As for their human counterparts, FADD was supersaturable whereas APAF1^CARD^ was not (Figure 5C), suggesting an ancient role of supersaturation in extrinsic apoptosis that may precede metazoans.

## Discussion

In this work we sought to interrogate whether nucleation barriers encoded in the protein modules that orchestrate immune responses act as cell fate determinants. Using DAmFRET to characterize DFD phase behaviors, we found that seventeen protein modules encode nucleation barriers that allow them to achieve deeply supersaturating concentrations in living cells.

### Nucleation barriers are a central aspect of innate immune signaling

DFDs with nucleation barriers tend to be central hubs and bottlenecks of information flow in innate immune signaling networks. They also tended to have lower solubilities and yet be expressed to higher levels *in vivo*, consistent with a high degree of supersaturation. Most of the adaptor proteins retained their nucleation barrier in their full-length context. We found for one exceptional case, MYD88, that a nucleation barrier occurred in an upstream adaptor of the TIR domain family. Our results indicate that nucleation barriers are widespread among the modules controlling innate immune signaling.

### Nucleation barriers may enhance sensitivity

Supersaturation provides a thermodynamic driving force for signal amplification by allowing for all molecules above the solubility limit to be activated “for free”. This theoretically reduces the fraction of total molecules that need to be activated by directly interacting with — or “sensing” — ligand-bound receptors. We found that full length adaptor proteins, uniquely, tend to have even larger nucleation barriers with a relatively increased dependence on conformational fluctuations, than their corresponding DFDs in isolation. This was especially pronounced for the mitochondrial antiviral signaling adaptor, MAVS, which has been found to respond decisively to extremely low numbers of dsRNA molecules in the cytoplasm. Two exceptions to this trend are the apoptosome and the PIDDosome, which appear to entirely lack supersaturable components. These are also atypical in their having a finite oligomeric rather than polymeric structure (67, 68), which emphasizes the dependence of nucleation barriers on semi-crystalline ordering. These two signalosomes are also exceptional with respect to function— they respond exclusively to cell-intrinsic signals of stress rather than extrinsic signals of infection (69, 70). Hence their functions do not require the extreme sensitivity that nucleation barriers hypothetically offer.

### Nucleation barriers may enhance specificity

We also observed nucleation barriers among receptor DFDs, although in all tested cases, these appeared to be subject to autoinhibition in the corresponding full-length proteins. Are they nevertheless relevant to function? We speculate that the nucleation barrier provides a pause in activation once autoinhibition is released by ligand binding, and that this pause functions as a kinetic proofreading mechanism to limit “false-positive” activation on benign PAMP look-alikes. For example, full length AIM2 activation occurs preferentially on long dsDNA, where it can form larger oligomers sufficient to overcome a polymer nucleation barrier, and thereby ignores dsDNA fragments that would be too short for pathogenesis (2). Further work using protein modeling and molecular simulations, will be necessary to understand the physical origins of nucleation barriers.

### A plausible role for liquid-liquid phase separation in innate immune signaling

While our findings emphasize the unique kinetic functionality of ordering transitions, they do not exclude roles for LLPS in innate immune signaling. On the contrary, the unparalleled thermodynamic functionality afforded by LLPS is likely to be exploited here as in other signaling networks, as suggested by recent demonstrations of LLPS by certain DFD proteins (80). Our results suggest that LLPS by DFD-containing receptors will be most impactful for signaling. This is because receptor DFD assemblies tended to be more labile, and their nucleation more responsive to concentration changes. We previously showed for one such receptor, CARD9, that the full length protein forms autoinhibited multimers via multivalent coiled coil interactions, and that the lability of CARD9^CARD^ polymers prevents seeds from forming outside of these multimers (15, 24). The present findings with full length proteins reveal a common tendency of receptors to oligomerize or condense. Given that receptors tend to be expressed at lower levels than adaptors, we speculate that this activity will tend to be limited to the elevated local concentrations that occur upon binding multivalent ligands. These condensates would support seed formation just as we have shown here with μNS fusions. LLPS will have limited functionality in subsequent signal amplification, however, because it is stoichiometrically coupled to the number of ligand scaffolds. Signal amplification, and hence extreme sensitivity, requires supersaturation that in turn requires conformational ordering in the condensed phase. Further work using protein modeling and molecular simulations, will be necessary to understand the structural origins of the nucleation barriers.

### A network of nucleating interactions among DFDs

We reasoned that identifying nucleating interactions between DFDs could uncover a common framework for immune cell fate determination. Our systematic screen for such interactions revealed extraordinary specificity among DFDs, with only a tiny fraction of all possible DFD pairs resulting in enhanced nucleation. These were largely homotypic in nature, with DFDs tending to interact preferably with other members of their subfamily. Most of the exceptions to this trend occurred between the PYD members of the inflammasome and the DED and DD members of the DISC. Increasing evidence has suggested the formation of a macromolecular complex termed the PANoptosome that contains protein modules related to pyroptosis, apoptosis, and necroptosis (81). While the hierarchy of interactions remains unsolved, our findings show that it likely involves multiple DFDs interacting across subfamilies.

### Constitutive supersaturation in at least one programmed cell death pathway

We went on to validate one such interaction *in vivo*, by showing that signaling bifurcates upon activating the AIM2 receptor, with one cell death outcome driven by endogenously supersaturated ASC and the other outcome driven by FADD. In both cases, signaling required an intact polymerization interface for AIM2^PYD^, which gave rise to highly persistent seeds. This persistence likely lends AIM2 to PANoptosome formation, and may serve as a “backup” form of cell death signaling through the FADD pathway when pyroptosis is compromised. Our findings collectively provide strong evidence that programmed cell death in response to extrinsic stimuli is driven by supersaturation, *in anticipation of* pathogenic stimuli.

We observed slow and heterogeneous cell death via FADD signaling, a phenomenon also reported by other labs (75, 82, 83), and which has thwarted attempts to exploit extrinsic apoptosis in treating cancer (84). We suggest that it results from the stochasticity of nucleation and/or heterogeneity of effective FADD concentrations around its phase boundary.

### Supersaturation may drive age-associated inflammation

We posit that innate immune cells employ a network of nucleated polymers to ensure cell fate commitment and supersensitivity at the expense of having protein modules with an intrinsic ability to spontaneously activate. Innate immune cells are a primary source of inflammatory signals as organisms age. We speculate that the cumulative probability of “accidental” nucleation and activation of signalosomes triggering inflammation and programmed cell death increases with age, and thus may be associated with the onset of the chronic inflammatory state known as inflammaging. Altogether the approach outlined here paves the way to further dissect nucleation barriers and protein supersaturation networks in physiological systems.

## Materials and Methods

### Reagents and antibodies

Reagents include Hygromycin B (Invivogen, ant-hg-1), Penicillin-Streptomycin (ThermoFisher, 1514014gp), PMA (BioVision, 1544–5), Puromycin (Invivogen, ant-pr-1), Sytox Orange (ThermoFisher, S11368), CellTox (Promega, G8741). Antibodies, anti-FADD (sc-514414), anti-FADD (05–486), anti-Actin (sc-8432).

### Plasmid construction

Yeast expression plasmids were made as previously described in Khan et al 2018. Briefly, we used a golden gate cloning-compatible vector V08 which contains inverted BsaI sites followed by 4x(EAAAR) linker and mEos3.1. V08 vector drives the expression of proteins from a GAL promoter and contains the auxotrophic marker URA3. The vector V12 derives from V08 and consists of inverted BsaI sites after the mEos3.1 and 4x(EAAAR) linker to tag inserts at the N-terminus. Inserts were ordered as GeneArt Strings (Thermo Fisher) flanked by Type IIs restriction sites for ligation between BsaI sites in V08 and V12. All other inserts were cloned into respective vectors via Gibson assembly between the promoter and respective fluorescent or tag marker. All plasmids were verified by Sanger sequencing.

Lentivirus vectors were as previously described in Rodriguez Gama, et al 2022. Briefly, AIM2-miRFP670-Cry2clust optogenetic constructs were cloned into pLV-EF1a-IRES-Hygro (Addgene #85134) which encodes a HygromycinB resistance cassette. To create lentiviral vectors for the expression of AIM2 fused with miRFP670 and Cry2, we inserted via Gibson the coding sequence of cloned inserts in m25 into pLV-EF1a-IRES-Hygro. Finally, for the doxycycline-controlled lentiviral vectors, we cloned the respective coding sequences from *PYCARD* into pCW57.1 (Addgene #41393).

### Yeast strain construction

For expression of expression clones for DAmFRET experiments, we employed strain rhy1713, as previously described in Khan et al 2018. To create artificial intracellular seeds of protein modules, sequences were fused to a constitutive condensate-forming protein, μNS (471–721), herein “μNS”. Yeast strain rhy2153 was created by replacing the HO locus in rhy1734 with a cassette consisting of natMX, the tetO7 promoter, and a tandem pair of counter selectable *URA3* ORFs derived from *C. albicans* and *K. lactis*, followed by µNS-mCardinal. To create strains expressing the fusion protein modules-μNS and others, AseI digests of yeast expression plasmids containing desired proteins were transformed into rhy2153 to replace the counter-selectable *URA3* ORFs with the gene of interest. The resulting strains express the proteins of interest fused to μNS-mCardinal, under the control of a doxycycline-repressible promoter. Transformants were selected for 5-FOA resistance and validated by flow cytometry for the correct expression of intracellular seeds.

### Nucleating interaction library construction strategy

To create the nucleation interaction matrix we created two yeast libraries. One yeast library expresses the “target modules” from mEos3.1 tagged expression clones. The second library expresses the “seeds” from the doxycycline-repressible promoter. We then mated both libraries and arranged them by “target module”. This way we would measure the effect of the “seeds” over “target modules” during the same collection time. First, both yeast libraries were pinned onto agar ommitrays (Thermo Scientific 242811) SD-URA for ‘target modules library and YPD for “seeds”. Once both libraries grew on agar they were mated onto agar ommitrays (SD-URA+NAT+Dox). After this step, mated colonies were pinned into liquid SD-URA+NAT+Dox for diploid selection and storage of glycerol stocks. The entire nucleating interaction screening consisted of 384 96-well plates.

### DAmFRET assay preparation and data collection

We performed DAmFRET analysis as previously described in Khan et al. 2018. Yeast transformants were selected in SD-URA plates. Briefly, single transformant colonies were inoculated in 200 μl of SD-URA in a microplate well and incubated in a Heidolph Titramax platform shaker at 30°C, 1350 RPM overnight. Cells were washed with sterile water, resuspended in galactose-containing media, and allowed to continue incubating for approximately 20 hours. Microplates were then illuminated for 25 min with 320–500 nm violet light to photoconvert a fraction of mEos3.1 molecules from a green (516 nm) form to a red form (581 nm). At this point, cells were either used to collect microscopy data or continue the DAmFRET protocol.

For the DAmFRET collection of the nucleating interaction, glycerol stock plates were pinned into liquid SD-URA without dox and incubated for 16 hours at 30°C, 1350 RPM overnight. We performed an SD-URA media change to recover cells for an additional 20 hours. After this protein expression was induced with SGal-URA for 20 hours. 4 hours prior DAmFRET data collection, we change media with fresh SGal-URA. 4× 96-well plates were combined into a 384-well plate, totaling 96× 384-well plates for the entire screening.

DAmFRET data was collected on a ZE5 cell analyzer cytometer. Autofluorescence was detected with 405 nm excitation and 460/22 nm emission; SSC and FSC were detected with 488 nm excitation and 488/10 nm emission. Donor and FRET fluorescence were detected with 488 nm excitation and 425/35 nm or 593/52 nm emission, respectively. Acceptor fluorescence was excited with 561 nm excitation and 589/15 nm emission. For each well, we collected a volume of 13 uL, resulting in approximately 50,000 events per sample. Data compensation was done in the built-in tool for compensation (Everest software V1.1) on single-color controls: non-photoconverted mEos3.1 and dsRed2 (as a proxy for the red form of mEos3.1). For nucleating interactions, all regular channels for DAmFRET were collected with the addition of a collection of mCardinal intensity by 561 nm excitation and 670/30 nm emission.

### DAmFRET data analysis

Data were processed on FCS Express Plus 6.04.0015 software (De Novo). Events were gated for single unbudded cells by FSC vs. SSC, followed by gating of live cells with low autofluorescence and donor positive. Live gate was then selected for double positives (donor and acceptor). Plots represent the distribution of AmFRET (FRET intensity/acceptor intensity) vs. Acceptor intensity (protein expression).

For automated analysis of DAmFRET data, we follow the process described in Kandola et al. 2021. Briefly, FCS files were gated using an automated R-script running in flowCore. Before gating, the forward scatter (FS00.A, FS00.W, FS00.H), side scatter (SS02.A), donor fluorescence (FL03.A) and autofluorescence (FL17.A) channels were transformed using a logicle transform in R. Gating was done using gating for cells using FS00.A vs SS02.A then selecting for single cells using FS00.H vs FS00.W and finally selecting for expressing cells using FL03.A vs FL17.A. Cells falling within all of these gates were then exported as FCS3.0 files for further analysis.

We initially attempted to classify each DAmFRET dataset as one-state or two-state, and discontinuous or continuous for the latter, using an algorithm previously developed for this purpose (42). However, the algorithm invariably misclassified discontinuous datasets as “continuous” when the AmFRET level of the high FRET state changed with concentration, i.e. exhibited positive or negative slope as for FAS^DD^ and CASP2^CARD^, respectively. For this reason, we used the following approach.

DAmFRET histograms were divided into 64 logarithmically spaced bins across a pre-determined range large enough to accommodate all potential data sets. The upper gate values were determined for each bin as the 99th percentile of the DamFRET distribution in that bin. We defined an are corresponding for the expression clone x0935 which expresses mEos3.1 alone as the negative FRET gate for no assembly. For all samples, cells falling above this negative FRET gate can be said to contain assembled (FRET-positive) protein. A metric reporting the gross percentage of the expressing cells containing assembled proteins is therefore reported as fgate, a unitless statistic between 0 and 1.

This gate is then applied to all DAmFRET plots to define cells containing proteins that are either positive (self-assembled) or negative (monomeric). In each of the 64 gates, the fraction of cells in the assembled population was plotted as a ratio to the total cells in the gate.

These values were then fit to a Weibull function from which the statistics EC50, δ, and their respective errors (reported as the square of the residuals) were extracted (Khan et al. 2018). The confidence of the Weibull fit was defined as chi-square and the number of iterations to achieve a confident fit (Iter). We verified for all samples where conclusions are drawn from δ and/or EC50 that the Weibull fit closely approximated the raw DAmFRET plot.

### Determination of continuity

To determine the continuity of an adequately expressed expression clone, as defined using the same guidelines as determining positive nucleation interactions, we selected a window of +/− 14% expression range around the computed EC50. Using the Hartigan & Hartigan’s dip test for unimodality performed around this region, we determined that a “discontinuous” DAmFRET profile results with a confidence of p value less than 0.05. If p value was larger the profile was determined “continuous.” The resulting continuous profiles, were then subjected to further analysis using the fgate parameter, where if greater than or equal to 0.8 it was classified as “robust self-assembly”. We then verified the accuracy of this analysis by visual inspection of DAmFRET plots.

### Determination of positive nucleating interactions

We first performed quality control of the DAmFRET data. Files containing at least 2500 cell events positive for meos3.1 were further processed. We assumed that a positive nucleating interaction results in the nucleation of target domains. In the DAmFRET analysis, we expect two major changes, a decrease in the EC50 and an increase in the fraction assembled (fgate). We then performed outlier identification for both parameters. We applied the 1.5*Interquartile rule (IQR) to identify the outliers on the lower end of the EC50 and the upper end of fgate values. We used a multiparameter determination to extract the DFDs in the form of seeds capable of modifying the average, normalized, degree of outlier for each fgate and EC50. Values greater than or equal to 0.5 were determined hits. For each target module, we measured the effect of 192 “seeds” which provided a distribution of the effect on nucleation of positive “seeds” we included two negative controls.

### Cell culture

HEK293T cells and THP-1 cells were purchased from ATCC. THP-1 *PYCARD*-KO and GSDMD-KO were purchased from InvivoGen. HEK293T cells were grown in Dulbecco’s Modified Eagle’s Medium (DMEM) with L-glutamine, 10% fetal bovine serum (FBS), and PenStrep 100U/mL. THP-1 cells were grown in Roswell Park Memorial Institute (RPMI) medium 1640 with L-glutamine and 10% FBS. All cells were grown at 37°C in a 5% CO2 atmosphere incubator. Cell lines were regularly tested for mycoplasma using the Universal mycoplasma detection kit (ATCC, #30–1012K).

### Generation of stable cell lines

For the generation of stable cell lines, we followed the steps detailed in Rodriguez Gama et al 2022. Briefly, constructs were packaged into lentivirus in a 10 cm plate 60% confluent of HEK293T cells using the TransIT-LT1 (Mirus Bio, MIR2300) transfection reagent and 7 μg of the vector, 7 μg psPAX2, and 1 μg pVSV-G. Lentivirus was harvested and incubated with 293T with polybrene or infected at 1000xg for 1 hour for THP-1 cells. For transduction of pCW57.1 derived vectors, HEK293T and THP-1 cells were selected with Puromycin (1 ug/mL) for 7 days. After this time, cells were sorted for positive expression of mScarlet and expanded in continuing selection with puromycin. For transduction of expression clones tagged with miRFP670nano-Cryclust, THP-1, and HEK293T cells were selected with HygromycinB (350 ug/mL and 150 ug/ml, respectively) for 7 days. Cells were sorted for positive expression of miRFP670nano and expanded for further experiments with continued selection.

### Fluorescence microscopy and optogenetic activation

The yeast and mammalian cells were imaged in an LSM 780 microscope with a 63x Plan-Apochromat (NA =1.40) objective. T-Sapphire excitation was done with a 405nm laser. mEos3.1 and mScarlet-I excitation were made with a 488 nm and 561 nm laser respectively. For time-lapse imaging, samples were maintained at 37 °C and 5% CO2 with a stage top incubator. To stimulate Cry2clust we used the 488 nm laser at a power setting of 50% for a pulse of 10 seconds, which is the amount of time it took to scan the user-generated region of interest unless indicated otherwise. 561 and 633 nm lasers were used for imaging mScarlet-I and miRFP670nano, respectively. Pyroptosis events were tracked by incorporating the Sytox Orange reagent into the cell.

High-content imaging was performed on the Opera Phenix High-content screening system. Briefly, yeast strains expressing “target modules” were pinned into liquid SD-URA and performed mEos3.1 protein induction as described for DAmFRET assays. Instead of subjecting samples to the ZE5 flow cytometer, 10 ul were transferred into a well containing 90 ul of SGal-URA of a 96-well optically clear flat-bottom (PerkinElmer 6055302). Data analysis of the high content imaging was performed in Fiji following a series of image segmentation to identify individual cells. After this mEos3.1 intensity was determined and the coefficient of variation (CV) of pixel distribution was plotted against the aspect ratio (AR) of the meos3.1 pixel distribution.

For quantification of cell dead events using incucyte, THP-1 cells were plated on a 24 or 96 well plate at a density of 400e6/well or 100e6/well respectively with PMA (10 ng/mL) for 16 hrs. For AIM2-Cry2clust activation, an initial collection of unexposed measurements was taken for 30 min. Then the plate was exposed to 488 nm laser every 5 min. For treatments with polydA:dT cells were treated and immediately subjected to imaging every 30 minutes for 19 hours.

### Quantification and statistical analysis

Two-sided Student’s t-tests were used for significance testing unless stated otherwise for two sample comparisons. The graphs represent the means ± SEM of independent biological experiments unless stated otherwise. Statistical analysis was performed using GraphPad Prism 9, Python and R packages.

## Supplementary Material

Supplement 1 Supplementary figure legends
Figure S1
A schematic of the DFDs of all the proteins included in this screening, along with their classification into subfamilies.
Figure S2
A. DAmFRET profiles of some of the DFD proteins along with their classification as continuous, discontinuous profiles, and robust self-assemblies.
B. Distribution of protein localization among DFDs that exhibited high-AmFRET populations in the DAmFRET screen.
C. Analysis showing a dominance of fibrous morphology of DFDs that had a discontinuous DAmFRET profile.
D. Confocal microscopy panel showing the emergence of filaments from the μNS-fused seed.
Figure S3
DAmFRET profiles of representative DFDs as single domains and in the full-length context.
Figure S4
DAmFRET plots of several TIR domains showing a lack of supersaturability or discontinuity. PYCARD full-length is the positive control and mEos3.1-only serves as the negative control.
Figure S5
A. Boxplot comparing the distribution of EC50 between continuous and discontinuous DFDs included in our screen. Distributions were compared and found significantly different using Mann Whitney U (U=179, n_continuous_=17, n_discontinuous_=13, p=0.0044). The same DFDs included in panel C were used for this comparison.
B. Boxplot comparing the distribution of EC05 between continuous and discontinuous DFDs included in our screen. Distributions were compared and found significantly different using Mann Whitney U (U=162, n_continuous_=17, n_discontinuous_=13, p=0.0328). Only DFDs with an EC05 error less than 0.1 were included.
C. Boxplot comparing the distribution of Protein Abundance between continuous and discontinuous proteins included in panel B and C from our screen. Distributions were compared and found significantly different using Mann Whitney U (U=41, n_continuous_=17, n_discontinuous_=12, p=0.0074)
D. A scatter plot of Protein Abundance (ppm, vs EC50 showing their negative correlation with a Spearman R value of −0.4308 (p=0.0035). Protein Abundance values are taken from PAXdb: Protein Abundance Database.
Figure S6
A. Visual representation of the creation of our pairwise screening library. 147 DFDs expressed in a transformed plasmid tagged with mEos3.1 selected for with URA mated to 149 seeds tagged with mCardinal integrated into the yeast genome resulting in 22,706 pairwise combinations.
B. DAmFRET was run on all pairwise combinations. As a quality control, only those with a total cell count greater or equal to 2500 and a mean acceptor intensity greater or equal to 3.5 were considered for the analysis. DAmFRET plots of FADD^DED-DD^ with a negative seed (mEos3.1) and self seeded are shown. Positive seeding interactions (as shown by the self seeded example) have a lower, left shifted EC50 and an increase in the percentage of cells assembled (as determined by a gate defining low fret states, shown in orange)
C. Hits are determined by a multiparameter combination of the degree of EC50 outlier and the degree of fraction assembled outlier as defined by 1.5 times the interquartile range (IQR). Points on the graph are shaded by this parameter. Boxplots are showing those seeds that are determined to be outliers lying in the green box. The scatter plot shows the combination of EC50 and fraction assembled outliers indicated by those lying within the green box. Of these, hits are determined by a combined normalized degree outlier score of EC50 and fraction assembled greater or equal to 0.5.
Figure S7
The full version of the matrix shown in Figure 3A including full length proteins.
Figure S8
A. Cartoon depicting the doxycycline-inducible ASC-mScarlet that replaced endogenous PYCARD in THP-1 cells.
B. Representative blots from capillary Western analysis (Bio-Techne/ Protein Simple) of the engineered ASC-mScarlett construct alongside endogenous ASC (PYCARD). Actin was the loading control.
C. Quantification of the data showing significantly lower-than endogenous levels of ASC in the engineered construct at even the highest level of induction by Doxycycline.
Figure S9
Western blotting verifying the knock-out status of PYCARD and/or FADD in the respective engineered stable THP-1 cell lines.

Supplement 2
