# Protein supersaturation powers innate immune signaling

**DOI:** 10.1101/2023.03.20.533581

**Published:** 2024-03-03

**Authors:** Alejandro Rodriguez Gama, Tayla Miller, Shriram Venkatesan, Jeffrey J. Lange, Jianzheng Wu, Xiaoqing Song, Dan Bradford, Jay R. Unruh, Randal Halfmann

**Affiliations:** 1 Stowers Institute for Medical Research, Kansas City, MO.; 2 Department of Biochemistry and Molecular Biology, University of Kansas Medical Center, Kansas City, KS, USA

## Abstract

Innate immunity protects us in youth but turns against us as we age. The reason for this tradeoff is unclear. Seeking a thermodynamic basis, we focused on death fold domains (DFDs), whose ordered polymerization has been stoichiometrically linked to innate immune signal amplification. We hypothesized that soluble ensembles of DFDs function as phase change batteries that store energy via supersaturation and subsequently release it through nucleated polymerization. Using imaging and FRET-based cytometry to characterize the phase behaviors of all 109 human DFDs, we found that the hubs of innate immune signaling networks encode large nucleation barriers that are intrinsically insulated from cross-pathway activation. We showed via optogenetics that supersaturation drives signal amplification and that the inflammasome is constitutively supersaturated *in vivo*. Our findings reveal that the soluble “inactive” states of adaptor DFDs function as essential, yet impermanent, kinetic barriers to inflammatory cell death, suggesting a thermodynamic driving force for aging.

## Introduction

Innate immune signaling transduces small signals to robust cellular responses. At the extremes, human cells respond to single molecules of pathogen origin with explosive inflammatory cell death.^1–7^ This level of sensitivity and signal amplification is likely essential to defeat enemies that can otherwise rapidly self-replicate to catastrophic effect.

Signaling initiates when pathogen- or damage-associated molecular patterns (PAMPs or DAMPs) activate cognate receptor protein oligomers that then activate effector proteins such as transcriptional regulators and caspases, typically via one or more intermediary proteins known as adaptors. Most interactions between receptors, adaptors, and effectors involve death fold domains (DFDs), a large and ancient superfamily of homotypic interaction modules whose origin appears to have preceded eukaryotes.^8–11^ DFDs comprise the caspase recruitment domain (CARD), death domain (DD), death effector domain (DED), and pyrin domain (PYD) subfamilies.

The energy released by tiny numbers of PAMPs or DAMPs binding to receptors is far too small to drive innate immune responses directly. The signal must be amplified in an energy consuming process.^12–14^ Other signal transduction pathways achieve amplification by energizing signaling molecules using chemical fuel (e.g. ATP, GTP) or electrochemical gradients. In contrast, DFDs amplify signaling by forming stable open-ended polymers.^15–25^ The polymers activate downstream signaling factors by simply localizing them to high proximity.^26,27^ At sufficiently high concentrations in vitro or when overexpressed, some DFDs also template their own activation in a prion-like fashion without an overt energy source.^15–20,25^ Whether they also do so at endogenous concentrations *in vivo* is not yet clear. Other DFDs fail to polymerize or do so without self-templating activity.^19,28–36^ Therefore, despite consensus that DFD polymerization can provide a stoichiometric basis for signal amplification, the driving force for polymerization *in vivo* is not known.

Newly synthesized polypeptides lose free energy upon folding.^37,38^ However, a few proteins can become stuck in metastable “native” states that retain some of this energy to drive a future change in activity when the protein is triggered by a stimulus to fold into its more stable conformation.^39,40^ We posited that DFDs could also exist in inactive states that are metastable with respect to open-ended active polymers. The kinetic barrier for such a mechanism would be the initial formation, or nucleation, of the polymer. Nucleation occurs when a small number of protein subunits form an unstable embryo of the polymer that is just large enough to recruit more subunits.^41–43^ The amount of energy required for the nucleus to form spontaneously, known as the nucleation barrier, is determined by the protein’s supersaturation (concentration in excess of the polymer’s solubility limit) and its sequence-encoded conformational preferences in the physiological environment. In extreme cases such as bona fide prions, the sequence-encoded component of the nucleation barrier (hereafter referred to as the “structural nucleation barrier”) is so large in vivo that it preserves a deeply supersaturated state of the protein for years or decades before a nucleating event releases that energy.^44,45^ Importantly, nucleation involves a tiny fraction of the available molecules of the protein and can occur without any external change to the proteins’ folding landscape. This can allow for arbitrarily large numbers of proteins to activate in response to vastly substoichiometric stimuli. We previously showed that life has exploited this property in at least one case: the DFD adaptor for the CBM signalosome, BCL10, uses this principle to activate pro-inflammatory NF-κB.^46^ Owing to a sequence-encoded nucleation barrier, BCL10 is supersaturated even before stimulation and is therefore poised to respond decisively to fungal pathogen exposure. Is this a general paradigm for innate immune signaling, and if so, what advantage does it offer over classical signal transduction?

Effective signaling requires that a given signal be unequivocally linked to a specific response, while crosstalk between signaling pathways tends to be deleterious.^47,48^ Prion-like proteins notoriously interact with other cellular factors to cause disease. How then could such a mechanism underlie innate immunity, where the consequence of even a single aberrant interaction leading to “unintended” nucleation could kill the cell? Alternatively, might some level of nucleating crosstalk functionally increase cells’ robustness to pathogens?

These considerations lead us to hypothesize that DFDs function as phase change material in the biological equivalent of a thermal battery, wherein latent heat stored in a supersaturated phase is released on-demand by crystallization.^49,50^ This energy source would play the role of e.g. ATP in other signaling cascades, with the important difference that the signal-amplifying DFD is precharged for the role. Our hypothesis makes the following predictions: 1) some DFDs of innate immune signaling will be able to populate cell-wide soluble states that are supersaturated with respect to assembled states; 2) structural ordering and thermodynamic stability of the assembled states will increase with the kinetic stability of the soluble states that precede them; 3) those DFDs will be constitutively supersaturated *in vivo* (i.e. prior to stimulation); and 4) at least some of the DFDs from different signaling pathways will supersaturate independently of each other. We here use a combination of biophysical, bioinformatic, and cytological approaches in both orthogonal nonhuman and physiological human cellular contexts to investigate the capacity for supersaturation-driven signal amplification by all human DFDs. Our results collectively support all four predictions and thereby uncover a thermodynamic driving force for innate immune signaling.

## Results

### Nucleation barriers are prevalent among DFDs

We previously reported that several DFDs whose activation accompanies ordered polymerization -- MAVS, ASC, and Bcl10 can supersaturate in cells ^16,46^. Whether other DFDs share this property is central to our understanding of how innate immunity produces robust outputs from infinitesimal inputs. We used a sequence- and literature-guided search to identify 116 instances of DFDs in the human proteome, involving 104 human proteins (Table S1 and Figure S1A). Most of these are multidomain proteins with a single DFD at either terminus, a location that allows maximum accessibility for protein-protein interactions. Twelve proteins contained two DFDs, typically one closely following the other. To determine if the DFDs in such pairs should be evaluated separately or together, we used the Predicted Aligned Error (PAE) generated by AlphaFold.^51^ PAE is the expected positional error at residue x if the predicted and actual structures are aligned on residue y. Seven of the DFD pairs exhibited very low interdomain PAE scores comparable to those within the component DFDs (Figure S1B, bottom), suggesting a conserved fixed geometric relationship between the domains. We therefore considered these tandem DFDs as single members of their respective subfamilies, for a total of 109 DFD modules.

We sought to characterize the intrinsic phase behavior (sequence-encoded phase boundaries and kinetic barriers) of each DFD module, i.e. in the absence of other DFDs or regulatory interactions but under otherwise physiological conditions. For this purpose we used the budding yeast, *Saccharomyces cerevisiae*, as an orthogonal eukaryotic cell that completely lacks DFDs and their specific regulatory factors. We codon-optimized and cloned the DFDs for strong inducible expression as fusions to the bright and exceptionally monomeric amphifluorescent protein, mEos3.^52^ The fusion was made opposite the native N- or C-terminus of each protein to minimize non-native steric effects.

**Figure 1.**
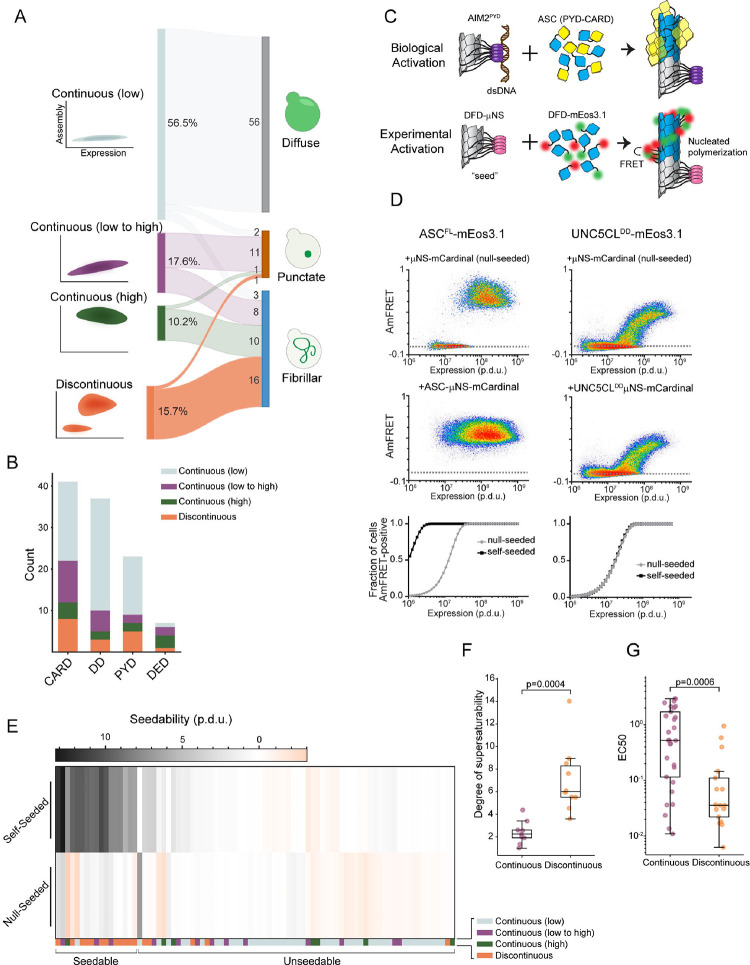
Nucleation barriers are prevalent among DFDs A. Illustration showing how the concentration-dependence of self-assembly as classified by DAmFRET (left) relates to the subcellular morphology of self-assemblies classified by high-throughput confocal microscopy (right). The number of DFDs in each link are indicated. B. Distribution of DAmFRET classifications across the four subfamilies of DFDs. C. Schematic of our experimental design to assess the ability of each DFD to seed itself. Top: Biological activation of the exemplary signalosome -- the Aim2 inflammasome -- occurs when the receptor Aim2 oligomerizes on the multivalent PAMP, dsDNA, and then templates the polymerization of the adaptor protein, ASC. Bottom: Experimental activation of potentially supersaturated DFDs was designed to mimic biological activation, by expressing each DFD in trans with the same DFD expressed as a fusion to the modular condensate-forming protein, μNS. D. Representative DAmFRET data contrasting a supersaturable protein (left) with one that is not supersaturable (right). Note that the supersaturated protein exhibits a discontinuous distribution of AmFRET across the expression range. The dashed horizontal line approximates the mean AmFRET value for monomeric mEos3. Procedure Defined Units (p.d.u). E. Heat map of seedability values showing that DFDs that have discontinuous DAmFRET classifications tend to be seedable. Procedure Defined Units (p.d.u). F. Degree of supersaturability represented as the fold change reduction in EC50. Discontinuous DFDs show a significantly higher reduction in the EC50 when compared to continuous DFDs. Mann Whitney U=1.0 (p=0.0004), n_continuous_ = 9, n_discontinuous_ = 10. See Degree of supersaturability under Materials and Methods, for details. G. Boxplot comparing the EC50 values of continuous and discontinuous DFDs when seeded. Discontinuous DFDs have significantly lower EC50 values, indicating greater polymer stability. Mann Whitney U = 179, n_continuous_ = 17, n_discontinuous_ = 13 (p = 0.0006). See also Figures S1, S2, and Table S1.

We then used DAmFRET to analyze the self-association of each protein as a function of its accumulated intracellular concentration following twenty hours of expression. DAmFRET produces a snapshot of the population-level distribution of single-cell measurements of ratiometric FRET (“AmFRET”) between the two fluorescent forms of mEos3. The data revealed a diversity of behaviors (Figure 1A, 1B, S1C, and Table S1), ranging from no self-association at any concentration to self-association in all cells at all concentrations. For many of the DFDs, cells formed both a low and a high AmFRET population with a continuous transition between the two populations. For 17 DFDs, the transition instead occurred discontinuously such that a range of DFD concentrations existed wherein cells with the same concentration populated either a low or high AmFRET value. These included the aforementioned DFDs of MAVS, ASC, and BCL10. We previously showed that discontinuous transitions result from large structural nucleation barriers that are characteristic of prion-like self-assembly.^43,46,53^ We find this to be a surprisingly large number given the paucity of proteins with known prion-like behavior. For example, a systematic DAmFRET screen of the top 100 computationally predicted yeast prion-forming proteins found that only 14 exhibited discontinuous DAmFRET profiles.^54^ We conclude that nucleation barriers are prevalent among DFDs.

Our hypothesis predicts that assemblies with structural nucleation barriers will tend to be more ordered and more stable than those lacking structural nucleation barriers. DFD polymerization can create microscopically visible filaments in cells.^18,46,55^, whereas less-ordered assemblies tend to form spherical or amorphous puncta. To probe the relationship of structural nucleation barriers to ordered polymerization, we therefore examined the intracellular distribution of each DFD using high-throughput confocal microscopy. We found that all of the DFDs that populated AmFRET-positive states also formed visible assemblies, whereas only five of the 61 DFDs with entirely low AmFRET did so (Figure 1A). We further classified the assemblies as either fibrillar or punctate based on the aspect-ratios and coefficients of variation in cellular fluorescence (Figure S1D). Each of the DFD subfamilies exhibited a diversity of behaviors (Figure 1B). As predicted, however, the assemblies of DFDs that had transitioned discontinuously were almost invariably fibrillar (16 of 17, Figure S1E, S1F and S1G). So too were those of DFDs with continuous high DAmFRET (10 of 11). In contrast, only 8 of 19 DFDs with continuous low-to-high DAmFRET formed fibrillar assemblies; the rest were punctate. Hence, the microscopy data confirm that discontinuous DAmFRET profiles of DFDs involve highly ordered polymerization, but not necessarily the other way round.

To next probe the relationship of structural nucleation barriers to polymer stability, we eliminated supersaturation to reveal each DFD’s saturating concentration, which is determined by the strength of subunit interactions in its assembled structure. We did so by introducing artificial genetically-encoded “seeds” of each DFD. We designed these seeds to mimic the oligomerized states of full-length DFD proteins when they are naturally activated by PAMPs or DAMPs, by genetically fusing each DFD to an inert and well-characterized homomultimeric domain, μNS^56^ (Figure 1C). We expected the resulting multimer to eliminate the nucleation barrier of the corresponding DFD expressed in trans, causing all cells with supersaturating concentrations of that DFD to switch from the low to the high AmFRET population^46^ (Figure 1D). As expected, co-expressed self-seeds caused cells to switch to the high AmFRET population for most (12 of 17) discontinuous DFDs (Figure 1E). Analyzing two such examples by fluorescence microscopy, we observed a corona of filaments radiating outward from the μNS puncta (Figure S1H, bottom left and top right). To control for potential nonspecific effects of μNS-DFD fusions, we also expressed these two DFDs in the presence of each other’s seed. Seeding did not occur in these cases (Figure S1H, top left and bottom right), confirming that it requires homotypic interactions between the seed and the corresponding DFD. Inspection of the DAmFRET plots of the five nonseedable discontinuous DFDs reveal that most are only slightly discontinuous. They include CARD9^CARD^, which we previously showed to polymerize with a negligible nucleation barrier above its solubility limit of approximately 100 μM.^19^ Hence, at least some of these five are false-positive in the unseeded analysis, presumably as a consequence of cell-to-cell heterogeneity in e.g. volume.^57^ Most of the continuous DFDs (61 of 66) did not exhibit self-seeding (Figure 1E, Table S1). As a proxy for saturating concentration, for each self-seeded DFD, we obtained the concentration at which half of cells exhibited AmFRET.^54^ By comparing this value (EC50) to the corresponding values obtained in the absence of seed, we found that discontinuous DFDs achieved soluble concentrations that exceeded their saturating concentration by more than six-fold on average (Figure 1F). More importantly, and as predicted, discontinuous DFDs exhibited lower saturating concentrations than continuous DFDs (Figure 1G, and Table S1), confirming the expected relationship between structural nucleation barriers and polymer stability.

While our data thus far confirm that discontinuous DFDs undergo nucleation-limited self-assembly, it does not clarify if such assembly involves native DFD interactions rather than amyloid-like misfolding.^43^ To answer that directly, we next introduced point mutations to disrupt assembly via conserved known interfaces between folded DFD subunits.^18,19^ Across multiple DFDs examined, all such mutations indeed reduced or eliminated the high-AmFRET population (Figure S2). To more systematically evaluate the nature of DFD assembly in our experiments, we subjected the seeded and unseeded cells to semi-denaturing detergent-agarose gel electrophoresis (SDD-AGE), a technique that distinguishes amyloids from other protein states based on their detergent-resistance and size dispersity.^58^ We found that unlike our amyloid control (RIPK1^RHIM^), none of the DFD assemblies survived sarkosyl exposure (Figure S1I), consistent with their retaining the death fold rather than misfolding into amyloid. These data collectively suggest that multiple DFDs encode nucleation barriers large enough for them to accumulate to supersaturated levels while staying soluble over organismal timescales.

### Supersaturation is a defining feature of innate immune signaling hubs

**Figure 2.**
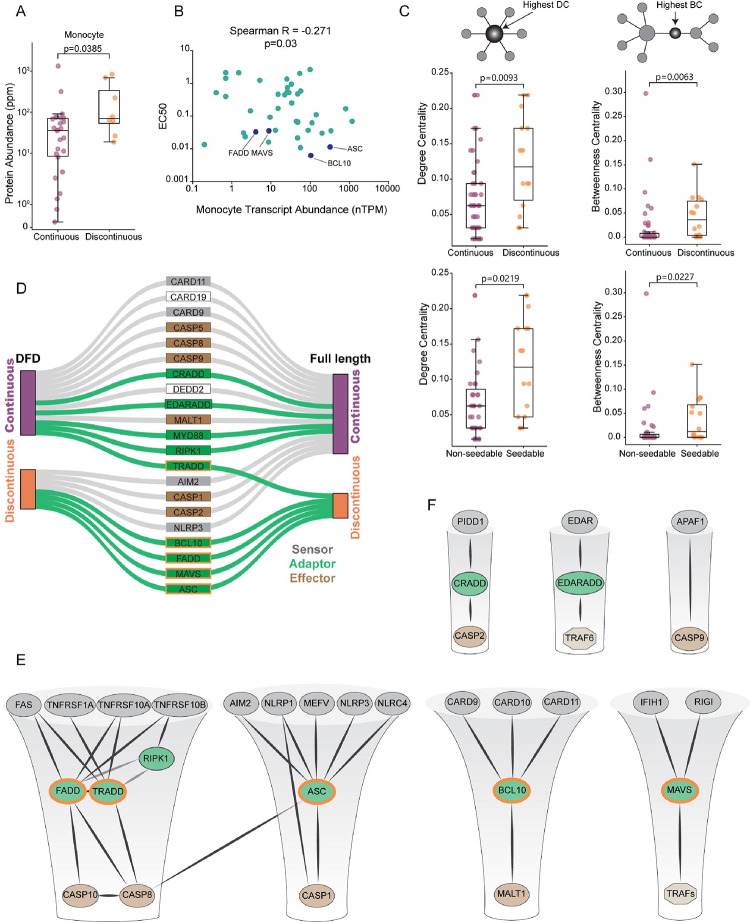
Supersaturation is a defining feature of innate immune signaling hubs A. Boxplot comparing the endogenous expression levels of continuous and discontinuous DFD-containing proteins indicating discontinuous DFDs have higher protein abundances in monocytes. Mann Whitney U = 40, n_continuous_ = 27, n_discontinuous_ = 8 (p = 0.0385). Protein Abundance values are from PAXdb.^61^ B. Scatter plot of Monocyte normalized Transcript per Million and EC50 values for self-seeded DFDs. Spearman R = −0.271 (p = 0.03). Adaptor DFDs are highlighted. Dataset extracted from Human Protein Atlas C. Top, box plots of degree centrality and betweenness centrality of discontinuous and continuous DFDs in the endogenous network of physically interacting DFD proteins, revealing that the former are more centrally positioned in the network. For both centrality measures n_continuous_ = 50, n_discontinuous_ = 14. For degree centrality, Mann Whitney U = 190.5 (p = 0.0093). For betweenness centrality, Mann Whitney U = 188.5 (p = 0.0063). Bottom, box plots of centrality measures of non-seedable and seedable DFDs as determined in Figure 1E. For both centrality measures n_non-seedable_ = 35, n_seedable_ = 16. For degree centrality, Mann Whitney U = 167.5 (p = 0.0219). For betweenness centrality, Mann Whitney U = 172.5 (p = 0.0227). D. Visualization of how the DAmFRET profiles of isolated DFD domains (left) change in their full-length contexts (right), showing that only adaptor proteins (green connections) tend to retain discontinuous transitions in their full-length context. E. Subnetworks of prominent signalosome adaptor proteins that were found to be supersaturable. Edges connect nodes with experimentally determined physical interactions with confidence > 0.9 in STRING. All proteins shown have DFDs except TRAFs. F. Subnetworks of signalosomes lacking supersaturable DFDs. Edges connect nodes with experimentally determined physical interactions with confidence > 0.9 in STRING. All proteins shown have DFDs except TRAF6. See also Figures S3 and S4, and Tables S1 and S2.

The depth of supersaturation *in vivo* is determined by the ratio of a protein’s expression level to its saturating concentration.^59^ Each term can evolve independently by gene regulation and DFD sequence, respectively. Consequently, if the function of discontinuous DFDs involves supersaturation, we can expect evolution to have increased their expression while decreasing their saturating concentrations, relative to those of continuous DFDs. Such a relationship would be unexpected in the absence of functional supersaturation, because most soluble proteins are only expressed to the edge of their saturating concentration.^60^ Having already assessed saturating concentrations, we therefore next evaluated the expression level of each DFD in monocytes using published transcriptomic and proteomic datasets curated by the Protein Abundance Database and the Human Protein Atlas.^61,62^ As predicted, discontinuous DFDs tended to have higher endogenous expression than continuous DFDs (Figure 2A, S3A and Table S1). This relationship persisted even in the whole body proteome (Figure S3B). We then asked how the saturating concentrations of DFDs relate to their expression levels in primary immune cells.^63^ Remarkably transcript levels significantly anticorrelated with saturating concentrations in all 18 canonical immune cell populations (Figure 2B and S3C), confirming that discontinuous DFDs are likely to be supersaturated in their endogenous physiological contexts.

A protein’s importance to a biological response can be approximated by its centrality in the corresponding network. Central proteins tend to be essential,^64^ more abundant,^65^ slower evolving,^66^ and more frequently targeted by pathogens^65,67^ than noncentral proteins. Proteins that have many direct interactions, or high “degree centrality”, are hubs in protein-protein interaction networks. Proteins that bridge many other nodes, or have high “betweenness centrality”, act as bottlenecks that disproportionately control information flow through the network. To gauge the importance of supersaturability to DFD signaling pathways, we therefore assessed these two fundamental centrality measures for all DFD-containing proteins in the network of physically interacting DFD-containing proteins, as extracted from the STRING database.^68^ Proteins with discontinuous and/or seedable DFDs proved to have greater degree and betweenness centralities than those with continuous or non-seedable DFDs (Figure 2C and S3D), consistent with greater control by the former over pathway activity and an essential executive function of supersaturation in innate immunity.

For a DFD nucleation barrier to function as hypothesized, the DFD must be supersaturated (soluble yet poised for polymerization) in the context of its full-length (FL) protein. Any interactions with other parts of the protein that either lower the nucleation barrier or increase thermodynamic solubility would reduce the extent of supersaturation and therefore capacity to drive signal amplification. Multiple DFDs that are otherwise capable of polymerization are prevented from doing so by autoinhibitory interactions in the context of full-length protein oligomers.^19,31–36^ Our screen of isolated DFDs already included five FL proteins that consist of little more than the DFD itself; none of these were supersaturable. We therefore evaluated the phase behaviors of 21 diverse DFD-containing FL multidomain proteins. Many of the proteins behaved comparably to their corresponding DFDs (Figure 2D, Figure S4A, and Table S2). Others had continuous low or moderate DAmFRET even though their isolated DFDs had polymerized discontinuously to high AmFRET, consistent with autoinhibited oligomerization. We confirmed this interpretation for one of these (NLRP3) by showing that seeds of the DFD, but not the FL protein itself, eliminated the nucleation barrier of its cognate adaptor (ASC; see below and Figure S4B). Five of the FL proteins were, however, comparably or even more supersaturable than their corresponding DFDs. These collectively represent all four DFD subfamilies: BCL10 and MAVS (driven by CARD), TRADD (driven by DD), FADD (driven by DED), and ASC (driven by PYD and CARD). Strikingly, all of these function specifically as adaptors in well-characterized signalosomes of innate immunity, where they link multiple PAMP or DAMP receptors to one or more effectors (Figure 2E). In contrast, signalosomes that are less specific to innate immunity, particularly the apoptosome, the PIDDosome, and the EDA receptor complex, lacked supersaturation (Figure 2F). These data reveal that interactions of the DFD with the surrounding protein context can either inhibit or enhance supersaturation, but that the latter occurs preferentially (if not exclusively) among adaptors with innate immune signaling functions. That these prion-like adaptor proteins are the hubs of their respective signaling networks agrees with their hypothesized executive function.

### Supersaturation drives signal amplification in human cells

We next asked if supersaturation drives signal amplification *in vivo*. We focused on the intrinsic pathway of apoptosis, by which cell death normally occurs in response to persistent intracellular stresses.^69–71^ Consistent with the relatively muted signaling of this pathway relative to, for example, pyroptosis, we found that the apoptosome lacks supersaturable DFDs (Figure S5A).

**Figure 3.**
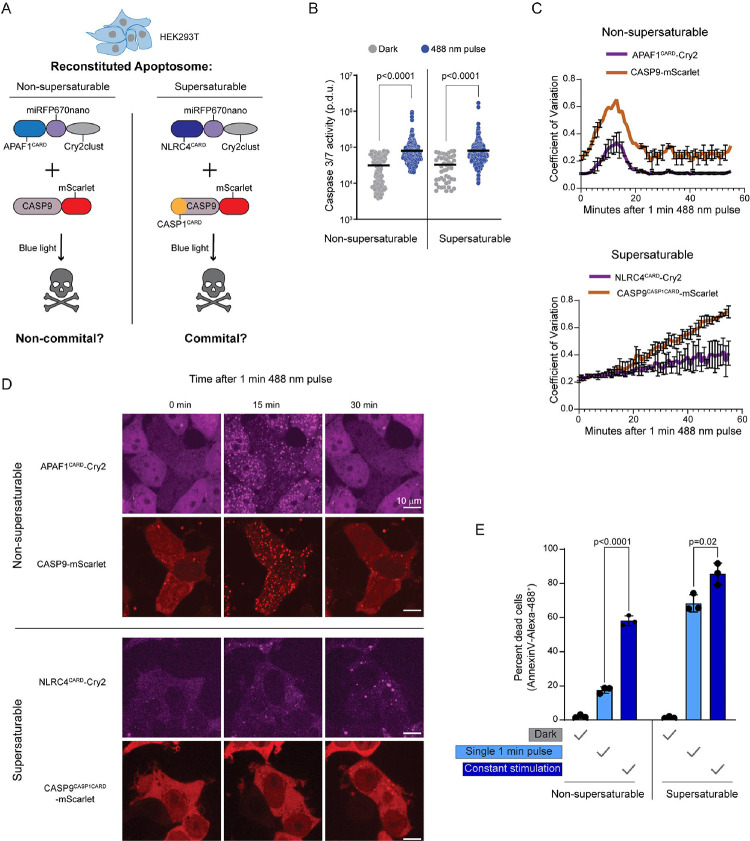
Supersaturation drives signal amplification in human cells A. Schematic of experiment in HEK293T cells to reconstitute the apoptosome with optogenetic control, in either a nonsupersaturable or supersaturable format. The nonsupersaturable format comprises the typical APAF1^CARD^ and CASP9 pair; the supersaturable format comprises the chimeric APAF1 with NLRC4^CARD^ in place of APAF1^CARD^ and chimeric CASP9 with CASP1^CARD^ replacing CASP9^CARD^ (CASP9^CASP1CARD^). B. Caspase 3/7 activity reporter fluorescence intensities in the absence of stimulation or after 1 min 488 nm stimulation for cell lines expressing the nonsupersaturable or supersaturable pairs, showing that both pairs signal comparably to CASP3 while oligomerized. P-value derived from Mann-Whitney analysis. APAF1^CARD^-Cry2 + CASP9-mScarlet, Dark n = 163, Pulse n = 375. NLRC4^CARD^-Cry2 + CASP9^CASP1CARD^, Dark n = 46, Pulse n = 305. C. Coefficient of variation (CV) of fluorescence distribution in HEK293T cells expressing the indicated protein pairs after a single 1 minute 488 nm laser activation. Top, APAF1^CARD^-Cry2 and CASP9-mScarlet display rapid cluster formation that dissociates by 20 min. Bottom, NLRC4^CARD^-Cry2 and chimeric CASP9^CASP1CARD^ cluster less rapidly but the clusters continue to grow indefinitely. D. Representative images from experiment in C. Clusters of APAF1^CARD^-Cry2 and CASP9-mScarlet form then dissociate while NLRC4^CARD^-Cry2 and CASP9^CASP1CARD^ crusters only get larger. E. Quantification of cell death of the HEK293T chimeric cells (as in A) using Annexin V-Alexa 488 staining, either two hours after a single 1 minute pulse of 488 nm laser, or after two hours of “constant” stimulation whereby cells were subjected to a 1 second pulse every 1 min. p-value derived from t-test. See also Figure S5 and Table S3.

To test if the absence of nucleation barriers contributes to noncommittal signaling through the apoptosome, we adapted an optogenetic approach that exploits the reversible oligomerization of Cry2clust in response to 488 nm blue light^26,46,72^ to allow for precise experimental control over apoptosome initiation. HEK293T cells lack the inflammasome constituents NLRC4, ASC, and CASP1, allowing us to repurpose their DFDs in this cell line. Accordingly, we transduced the cells with mScarlet fusions of either the non-supersaturable WT apoptosome effector, CASP9, or a chimeric version that harbored the supersaturable DFD of CASP1 in place of its own (CASP9^CASP1CARD^). We simultaneously transduced the cells with blue light-inducible seeds of the cognate upstream DFD -- opto-APAF1 or opto-NLRC4, respectively (Figure 3A). Cry2clust oligomerizes within seconds after stimulation and then reverts back to its monomeric form over several minutes.^72,73^ Therefore, to first compare the abilities of the reconstituted apoptosomes to transmit signals irrespective of amplification, we measured the activation of CASP3/7, the downstream target of CASP9, after just one minute of blue light stimulation. We found that both cell lines activated a fluorescent CASP3/7 reporter to the same extent at this timepoint (Figure 3B), revealing that the WT and chimeric protein pairs comparably activate CASP9 while they are oligomerized.

To now assess DFD-mediated signal amplification, we evaluated both the persistence of DFD assemblies and their ability to commit cells to apoptosis following an otherwise sublethal stimulus. We anticipated that cells expressing the non supersaturated pair of apoptosome proteins will 1) form reversible clusters and consequently 2) survive a short blue light stimulus. In contrast, cells expressing the supersaturated pair of proteins will 1) form irreversible clusters that prolong signaling, and consequently 2) die even after a short blue light stimulus. To test prediction 1, we stimulated the cells with blue light for one minute and monitored subsequent protein localization. We found that both proteins coalesced to visible puncta in essentially all cells within 15 minutes. Both of the CASP9 variants followed suit (Figure 3C,D). The WT puncta then dissolved over the course of the next ten minutes. In contrast, the chimeric puncta instead continued to grow at a constant rate for at least the next 40 minutes (Figure 3C,D), as expected.

To test prediction 2, we measured cell death two hours after the initiation of blue light stimulation for either one minute or the full two hours. Neither the WT nor chimeric pair of proteins induced cell death in the absence of blue light, as determined by an absence of cells staining with Annexin V (Figure 3E). The WT protein pair induced death in approximately 18% and 55% of cells following the short and long stimulation, respectively (Figure 3E), confirming the expected dose-dependence of signaling. The chimeric protein pair induced death in most cells even for the short stimulation, revealing that supersaturation amplified signaling to a greater extent than had been achieved by the non-supersaturated native DFDs of intrinsic apoptosis.

### Innate immune cell death is driven by endogenously supersaturated adaptors

**Figure 4.**
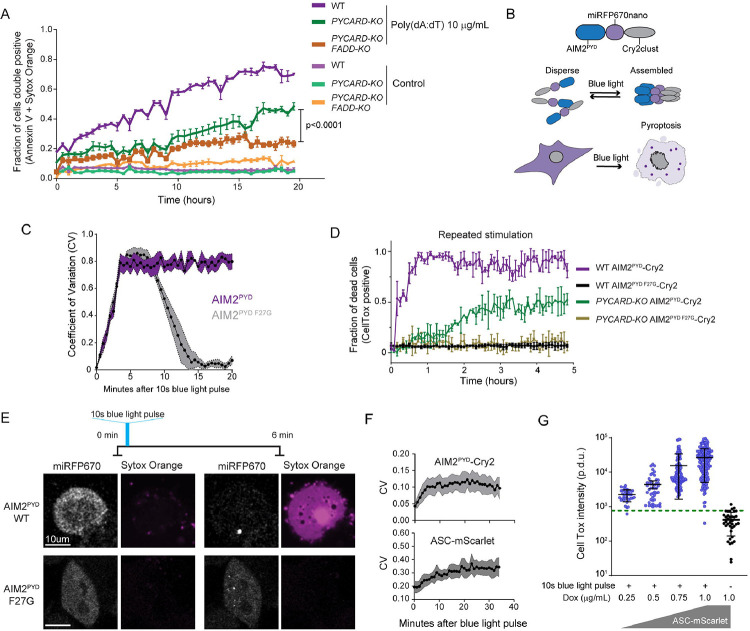
Innate immune cell death is driven by endogenously supersaturated adaptors **A.** Time course of apoptotic cell death of THP-1 cells following exposure to AIM2 ligand, poly(dA:dT). p-value obtained from ANOVA followed by pair comparison. B. Schematic of the experiment to transiently optogenetically stimulate AIM2^PYD^ to monitor the dynamics of ASC^PYD^ assembly. This experiment was conducted in HEK293T cells because they do not undergo pyroptosis. C. Time course of fluorescence intensity distribution in HEK293T cells following 10 seconds of optogenetic activation, showing that WT AIM2^PYD^ forms clusters (high CV) that persist, while the F27G mutant forms clusters that subsequently disperse. D. Time course of cell death of THP-1 cells when subjected to a blue light pulse every 5 minutes (“repeated”), showing rapid cell death (violet trace) only when AIM2^PYD^ is WT and when ASC is present. The absence of ASC results in slower death (green trace), consistent with apoptosis. The F27G mutation of AIM2^PYD^ blocks cell death irrespective of ASC (black and golden traces). E. Representative confocal microscopy images from a timelapse of individual THP-1 monocytes showing that transient optogenetic stimulation of WT but not F27G mutant of AIM2^PYD^ causes it to form puncta that coincide with cell death. Sytox Orange was used for this experiment because it can be excited without activating Cry2. F. Coefficient of variation (CV) of fluorescence distribution of AIM2^PYD^-Cry2 and ASC-mScarlet in THP-1 *PYCARD-KO* cells following a 10s blue light pulse. This shows that AIM2^PYD^ and ASC-mScarlet (with slightly delayed kinetics) rapidly form clusters that persist well after stimulus removal. ASC-mScarlet was induced to only ~20% of the ASC expression in WT cells using 1.0 μg/ml Doxycycline (Dox). G. Quantification of CellTox staining in individual ASC-mScarlet THP-1 *PYCARD-KO* cells 30 minutes after a 10 second blue laser pulse, at different levels of Dox-induced ASC-mScarlet expression. Green dotted line indicates 95 % confidence interval (CI) for background fluorescence intensity, above which cells were considered CellTox-positive. Error bars denote standard deviation. Control, n = 37. 0.25 μg/mL Dox, n = 36. 0.5 μg/mL Dox, n = 47. 0.75 μg/mL Dox, n = 113. 1 μg/mL Dox, n = 180. See also Figure S5 and Table S3.

We next asked if supersaturation functions in innate immunity at endogenous expression levels, by focusing on extrinsic apoptosis and pyroptosis in human THP-1 monocytes. We first treated the monocytes with poly(dA:dT), a ligand for the inflammasome receptor, AIM2. By 18 hours, approximately 70% of the cells were dead or dying, as determined by double positive staining with Sytox and Annexin V-Alexa-488. We then deleted *PYCARD* to determine if cell death depended on ASC, the adaptor for the inflammasome and pyroptosis. Death was delayed, but not eliminated (Figure 4A). Crosstalk has been observed between the inflammasome and extrinsic apoptosis signaling.^74–79^ We therefore also deleted *FADD*, the adaptor for extrinsic apoptosis, and found that death was reduced further still (Figure 4A and S5B). These data reveal that AIM2 activation triggers programmed cell death through both ASC and FADD.

To eliminate the potentially confounding activation of orthogonal dsDNA sensors,^80^ we fused AIM2^PYD^ to the miRFP670nano fluorescent protein followed by Cry2clust (Figure 4B). We found that when expressed in HEK293T cells -- which lack the downstream effectors of AIM2 that would otherwise kill the cells upon stimulation -- the protein rapidly and persistently clustered following a 10-second blue laser pulse. We next introduced the F27G mutation to disrupt a critical PYD-PYD interface.^18^ In contrast to WT, the mutant protein clusters dissociated within 15 minutes following the blue light pulse (Figure 4C), altogether confirming that this fusion protein -- hereafter dubbed “opto-AIM2” -- grants direct control over AIM2^PYD^ seed formation.

We therefore proceeded to express opto-AIM2 in THP-1 cells. We subjected the cells to 1 second of 488 nm laser excitation every 15 minutes while monitoring cell death through CellTox incorporation. This regime sufficed to kill essentially all cells within one hour (Figure 4D). In the absence of ASC (*PYCARD*-KO), approximately 50% of cells still died, but now with delayed kinetics consistent with the known hierarchical activation of apoptosis when pyroptosis fails ^81–83^. Cell death was completely eliminated by the F27G mutation, irrespective of the presence of ASC, confirming that both modes of cell death involve AIM2 making canonical DFD polymer interactions.

We reasoned that if ASC and/or FADD are supersaturated, a pulse of blue light will suffice to kill the cells. As expected, we found that a 10-second blue laser pulse triggered persistent protein clustering and cell death within six minutes, as indicated by Sytox Orange accumulation in the cytoplasm and nucleus (Figure 4E). Persistent clustering and cell death were both eliminated by the F27G mutation, confirming their dependence on PYD-PYD interactions (Figure 4E).

We next directly investigated inflammasome assembly in response to blue light by reconstituting the *PYCARD*-KO with doxycycline-inducible ASC. To allow us to monitor ASC expression level and localization in live cells, we fused to its C-terminus mScarlet-I, a rapidly maturing and fully monomeric fluorescent protein.^84,85^, and expressed the protein to a lower level than endogenous ASC (Figure S5C–D) to ensure that it is not aberrantly supersaturated. We then tracked AIM2^PYD^ and ASC localization following a 10-second blue laser pulse. Both proteins began to cluster almost immediately. AIM2^PYD^ clusters then ceased growing by 10 minutes, whereas ASC clusters continued to grow for the full duration of the half hour time course (Figure 4F), confirming that a strong thermodynamic drive for ASC self-assembly exists even in the absence of stimulus.

ASC polymers serve as a platform for CASP1 molecules to activate themselves by self-cleavage in trans. Activated CASP1 then causes pyroptosis by activating gasdermin D (GSDMD) to form pores in the plasma membrane.^86^ We therefore also assessed membrane integrity via CellTox incorporation either before the pulse or 30 minutes after, a timepoint that coincides with the cessation of CASP1 activity and full GSDMD cleavage.^87^ The pulse indeed induced membrane permeabilization (Figure 4G). The efficiency of ASC clustering (Figure 4F) and the fraction of cells staining with CellTox (those above the dashed line in Figure 4G) did not depend on its expression level even when reduced to 10% that of endogenous (Figure S5C–E). These data, along with the specificity of our stimulus and the rapidity of the response, lead us to conclude that endogenous ASC exists in a deeply supersaturated state prior to stimulation.

Multiple mechanisms beyond ASC polymerization can in principle amplify signaling via the inflammasome. Nevertheless, we observed that the intensity of CellTox staining at the cellular level increased with ASC expression across the range tested (Figure 4G). Given the low (~50 nM) saturating concentration for ASC polymers,^88^ this observation suggests a stoichiometric dependence of signal amplification on polymerized ASC subunits.

### The nucleating interactome is highly specific

The evolution of signaling networks is limited by crosstalk between pathways.^47,48^ Given that DFDs all share the same fold and are co-expressed in many of the same cells, they should be highly susceptible to nucleating each other. This would be functional when it occurs between DFDs in the same pathway, but likely deleterious when it occurs between DFDs in different pathways. To determine specificity and systematically map the nucleating interactome of DFDs, we mated our library of seed-expressing yeast strains with a library of strains expressing each mEos3-fused DFD (Figure S6A) to create over 10,000 arrayed diploid yeast strains that we then systematically screened by DAmFRET (Figure S6B and C).

**Figure 5.**
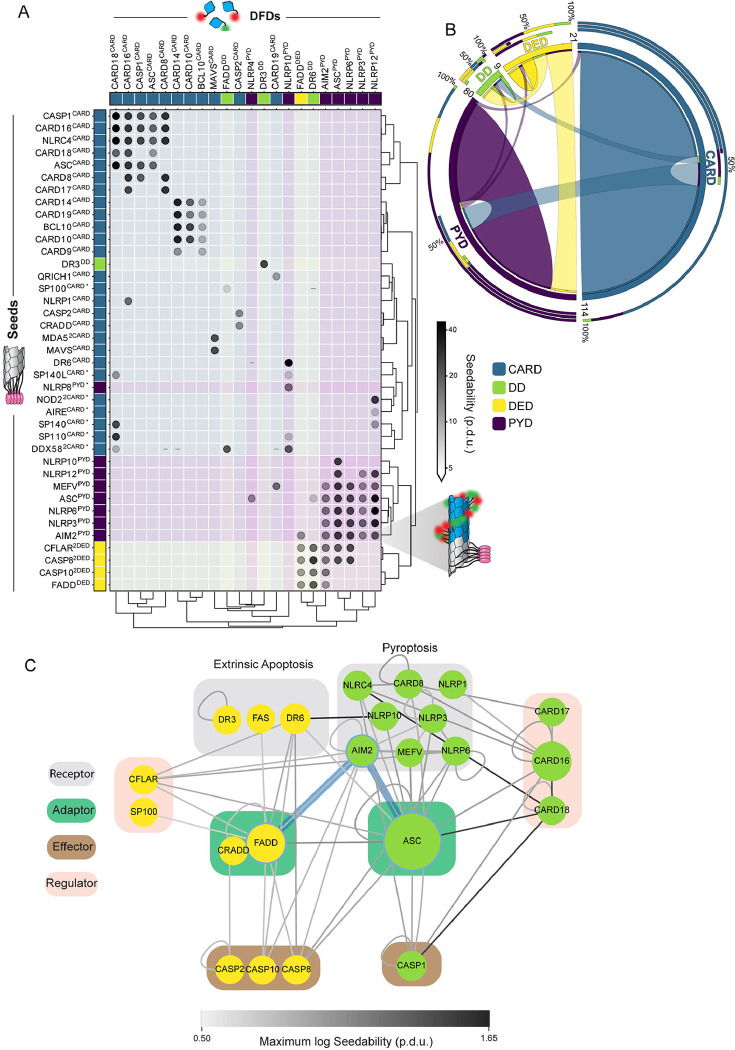
The nucleating interactome is highly specific A. Matrix of all nucleating interactions (gray-shaded circles) detected in a comprehensive DAmFRET screen of > 10,000 DFD pairs. Each DFD-mEos3 (columns) was separately expressed with each DFD-μNS seed (rows). Darker shading of the circle denotes increased seedability. Interactions among members of the same signaling pathway (in legend) appear in color shaded squares. Asterisk denotes seeds that were screened in a separate experiment from the rest. The matrix was clustered on seedability values, on a log scale, using the scipy.cluster.hierarchy v1.11.1 linkage and dendrogram Python packages, using the Ward variance minimization algorithm to calculate distances. Procedure Defined Units (p.d.u). B. Circos plot of the nucleating interactions summarized by DFD subfamily. Each subfamily is represented with a segment proportional to the number of DFDs with a nucleating interaction, as indicated by ribbons within and between segments. Inner stacked bars around the perimeter show the numbers of DFDs in each subfamily seeded by the subfamily in that segment. Middle stacked bars around the perimeter show the numbers of DFDs in each subfamily that seed the subfamily in that segment. Outer stacked bars around the perimeter show total nucleating interactions involving the subfamily in that segment. C. Nucleating interactions involving DFDs in extrinsic apoptosis and pyroptosis, with blue edges highlighting the direct nucleating effect of AIM2 on FADD and ASC that is explored in Figure 4. The network was created in Cytoscape with node size corresponding to betweenness centrality and grouped by reported function. Interactions between full length proteins (Table S2) were included. Edge darkness indicates the seedability score of the corresponding interaction. See also Figure S6 and Table S4

In total, we identified 171 nucleating interactions, representing just ~1.5% of the total library. These were consistently detected and highly reproducible in replicate experiments (Figure S6D–F and Table S4). Most of the interactions were homotypic (within the same subfamily; Figure 5A and B) and recapitulated known signaling relationships, such as DDX58 activating MAVS in the antiviral MAVS signalosome;^17^ CARD9 activating BCL10 in the antifungal CBM signalosome;^19^ and NLRP3 activating ASC and in turn CASP1 in the inflammasome.^18^ Moreover, nucleating interactions were largely constrained to members of the same signaling subnetwork. For example, CARDs of the CBM signalosome nucleated each other but not CARDs of the inflammasome, and vice versa. We nevertheless did observe a small amount of crosstalk, most notably between the PYDs of the inflammasome and the DEDs of the Death-Inducing Signaling Complex (DISC) (Figure 5C). These nucleating interactions, for example between AIM2^PYD^ and CASP8^2DED^, are consistent with the close phylogenetic relationship between PYDs and DEDs,^89^ and may be responsible for the residual cell death in *ASC FADD* double knockout cells shown in Figure 4A. They are consistent with known endogenous crosstalk between pyroptosis and extrinsic apoptosis signaling that is critical for defense against certain pathogens.^76,78,82^

On the whole, the observed network of nucleating interactions confirm that DFDs from different pathways are generally independently supersaturable. The supersaturable DFDs are nevertheless hardwired via structurally-encoded nucleating interactions to specific autorepressed signal receptors and effectors, consistent with their soluble ensembles functioning to power innate immune signal amplification.

## Discussion

DFDs polymerize to transduce innate immune signals across the tree of life,^22–24^ but the energetics of how they do so had not been studied systematically. We investigated the possibility that soluble DFD ensembles store energy for signal amplification. We tested that hypothesis by comparing the supersaturabilities of all human DFDs in a tractable model of their physiological context, revealing that 17 of the 109 DFD modules exhibit nucleation barriers sufficient for them to globally supersaturate living cells. These nucleation barriers occurred preferentially in the hub of the DFD-containing protein interaction network, whereby they exert maximum control over information flow. We examined one such node in its endogenous context and confirmed it to be constitutively supersaturated. Whereas the vast majority of soluble proteins are expressed only to their respective thermodynamic solubility limits,^60^ DFDs exhibit the opposite trend, highlighting the functional relevance of their supersaturation.

Our findings challenge the implicit assumption that ASC and other signalosome adaptors are thermodynamically inhibited prior to stimulation.^1,90^ Instead, given that their soluble expression levels likely far exceed their saturating concentrations for active polymerization, kinetic barriers are the principal determinant of their activity. For a typical cell, the absence of a structural template appears to be the only thing preventing it from self-destructing at any moment.

### DFDs are phase change batteries

Our data altogether establish that adaptor DFDs of innate immunity are the biological equivalent of thermal batteries, wherein latent heat stored in a supersaturated “phase change material” is released by crystallization.^49,50^ The larger the structural nucleation barrier, the more supersaturated the material can be and the greater and more stable the charge stored by the battery. Since the structural nucleation barrier is dominated by the entropic cost of soluble subunits forming a nucleus that is, itself, soluble,^41,42^ the phase change material in innate immune batteries -- that is, soluble DFD ensembles -- will have evolved by natural selection *against* mutants that assemble too readily, as doing so would lower the nucleation barrier and therefore capacity of the battery. Once the nucleus forms, either spontaneously or through interactions with PAMPs or DAMPs, the battery discharges to drive amplification through near-crystalline polymerization.

We found that each of the major signalosomes has approximately one such battery. Some prominent innate immune signaling pathways appear to diverge from this trend, however. Most notable is the adaptor for the Myddosome, MYD88, which links the activation of Toll-like receptors (TLRs) to DFD-containing effector proteins,^91^ and which did not supersaturate in our experiments (Figure S4A). Our finding is consistent with the finite oligomeric structure of the Myddosome,^91,92^. The hypothetical battery of the Myddosome may lie upstream of MYD88. In particular, the non-DFD-containing adaptor protein, MAL, is typically required for Myddosome assembly^93–97^ and has been shown to form self-assembling filaments *in vitro*^98^ via its Toll/Interleukin-1 Receptor (TIR) domain. Alternatively, MYD88 may be a false negative in our experiments, perhaps as a consequence of the fact that, unlike other adaptors which transition from monomer to polymer, MYD88 activation involves a transition between different multimeric states^92,99^ that may not be discernibly different by DAmFRET. MYD88 was previously found to exhibit a nucleation barrier *in vitro*^100,101^ and to form switch-like and persistent assemblies at the subcellular level in vivo.^99^ Further study will be required to determine if MYD88 and/or MAL are supersaturated in vivo.

The other DFD-containing pathways that lacked structural nucleation barriers also lack strong connections to pathogen defense, specifically the intrinsic apoptosome, the PIDDosome, and the EDA pathway for ectoderm differentiation. We note that unlike the pathways with supersaturable adaptors, which execute life-or-death decisions informed by minute signals of pathogen infection, these pathways function in tissue homeostasis and differentiation informed by persistent sterile host-derived signals.^102,103^ Hence, our systematic comparison of all human DFDs identifies structural nucleation barriers specifically in pathogen defense pathways, consistent with the extraordinary degree of signal amplification necessitated by this function. We suspect that the battery-like function of DFDs is ancestral because pyroptosis-like defensive cell death appears to have preceded apoptosis and even the emergence of eukaryotes.^77^

### Implications for structure-function relationships

The functions that dictate DFD structure are presumed to emerge from the material polymers, and specifically, their unbounded stoichiometry.^22–24^ However, the polymers’ functions appear not to constrain DFD structure, based on the fact that arbitrarily condensing or even dimerizing innate immune effector proteins suffices to transduce signaling,^26,27,46^. This suggests that the specifics of DFD polymer structure are dispensable for effector activation. In contrast, the battery-like function of supersaturated DFDs does constrain polymer structure. The constraint is indirect. Because battery capacity increases with the near-inability of soluble “inactive” ensembles to activate, the structure of the “active” state necessarily becomes more ordered. This is a consequence of evolution eliminating soluble conformers that too readily self-associate, leaving only an ensemble that fails to self-assemble spontaneously despite a thermodynamic drive to do so. Interactions between subunits correspondingly strengthen to offset the entropic cost of polymerization.^41^ This perspective explains why strictly linear polymers, such as by DIX and SAM domains, are not employed in innate immunity despite their abundance in other signaling pathways.^104^ Relative to these polymers that are structurally less restrictive but fundamentally lack a nucleation barrier, DFDs polymerize through two-dimensional crystallization,^27^ which creates a tunable nucleation barrier. That this feature rather than other properties of DFDs explains their use in innate immunity is supported by the fact that innate immune signaling pathways have evolved from at least two other unrelated protein domains that likewise polymerize in effectively two-dimensions: TIR domains and amyloids, the latter of which are in most other contexts dysfunctional.^22,27^

### Functional logic of phase change batteries in immunity

Most signal transduction cascades use chemical fuel or electrochemical gradients to amplify signaling in the form of high-energy or otherwise short-lived states.^12–14,105^ Examples include phosphorylated proteins that are rapidly dephosphorylated, high local concentrations of molecules that diffuse away, ions that are rapidly pumped back across the membrane, or caspase heterodimers that irreversibly dissociate. These pathways are consequently “off” at steady state. Allowing the pathways to reset provides for more dynamic signaling that is also more forgiving of aberrant activation. By contrast, adaptor DFD polymerization represents the equilibration of an already high energy system. Notwithstanding negative feedback mechanisms such as autophagy^106^ that can rid the cell of the polymers in some circumstances, the polymerized “on” state is effectively irreversible. Indeed, the polymers can outlive the cell of origin and propagate inflammation when taken up by other cells.^15^

Why should innate immunity use this unusual and unforgiving mode of signal amplification? The most obvious explanation is that innate immune signaling is often terminal -- the cells differentiate or die, by necessity to protect the organism. Another reason may be that crystallization processes are inherently specific. The extraordinary specificity revealed in our cross-seeding analysis shows that despite their overtly similar structures, each supersaturated DFD stockpiles energy that is privately accessible only to itself, its immediate partners, and no other proteins in the cell. This not only allows each pathway to operate independently, it insulates them from other cellular processes and metabolic fluctuations that could aberrantly activate the pathway to immediately lethal consequence.^47,48,107^

Enzyme folds are evolutionarily constrained by the fixed properties of their substrate. By comparison, and despite their high specificity for self, protein self-assembles have few structural constraints. Amyloid formation is a “default” capability of polypeptides,^108^ and symmetric homo-oligomers tend to be susceptible to polymerization.^109^ It is therefore possible that the inexhaustible sequence space that can support structural nucleation barriers benefits the host in the never-ending evolutionary arms race against pathogens.

Phase change batteries discharge in response to nucleating stimuli. The relative lability of receptor polymers (Table S1) and their tendency to be autoinhibited by non DFDs agrees with current interpretations that they function as nuclei for adaptor DFD polymers when uninhibited by binding to PAMPs or DAMPs.^22–24^ Consistent with this role and the frequently multivalent nature of PAMPs and DAMPs, condensing DFDs in the absence of autoinhibitory non-DFD interactions generally eliminated barriers to polymer nucleation. That the multimeric nature of DFD polymers may heighten the sensitivity of signaling to PAMPs and DAMPs through cooperative effects is well-appreciated.^22–24^ Nevertheless, cooperativity is limited by the small sizes of the polymers’ nuclei,^21,110^ and is therefore insufficient to explain the use of polymers over finite oligomers.

**Figure 6.**
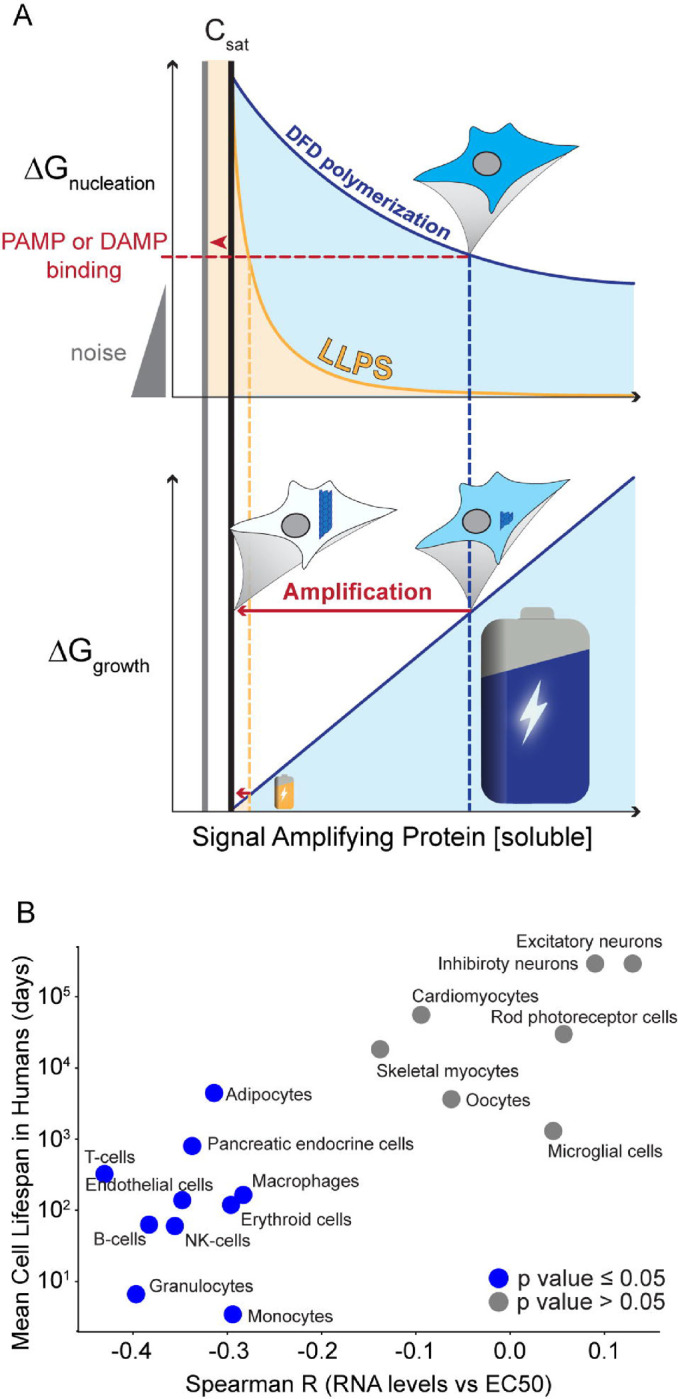
Structural nucleation barriers allow DFDs to act as phase change batteries A. Top: Nucleation barrier as a function of protein concentration. The nucleation barrier for phase separation is highest at the saturating concentration (C_sat_). For phase separation in the absence of structural ordering (LLPS, yellow solid line), the nucleation barrier declines precipitously with concentration above C_sat_. While this allows LLPS to respond to small changes in PAMP/DAMP concentrations or valencies that lower C_sat_ (red arrowhead), it prevents the system from achieving deep supersaturation. For phase separation concomitant with structural ordering (DFD polymer, blue solid line), the nucleation barrier falls more gradually with concentration above C_sat_, allowing the system to achieve deep supersaturation (dashed blue line). The energy released by PAMP or DAMP binding to a receptor protein complex can trigger nucleation (dashed red line). Bottom: Following nucleation, the polymers grow and deplete soluble protein until it is no longer supersaturated (solid red arrow). Hence the extent of supersaturation at the time of nucleation determines the extent to which the nucleating signal can be amplified. The structural nucleation barrier encoded by the soluble DFD ensemble therefore acts as a battery to power innate immune signal amplification. B. Scatter plot of the anticorrelation between DFD transcription level and EC50 versus mean lifespan for each cell type in the human body.^115^ Cell types with a significant anticorrelation, suggesting increased DFD supersaturation, have shorter lifespans than cell types without an anticorrelation.

A cooperativity function also fails to explain the use of polymerization over disordered condensation, which offers unlimited cooperativity near phase boundaries for liquid-liquid phase separation (LLPS).^111^ DFD-mediated polymerization has comparably limited ability to distinguish small differences in PAMP/DAMP concentration or valency. However, the absence of a structural nucleation barrier means that the susceptibility of LLPS to spontaneous nucleation increases dramatically beyond its phase boundary (Figure 6A).^41,43^ DFD proteins are therefore unlikely to be endogenously supersaturated with respect to LLPS, and their activation via this mechanism will be stoichiometrically limited by the concentration and valency of the respective PAMP/DAMP. In short, LLPS offers sensitivity but not signal amplification. However, coupling receptor LLPS to adaptor nucleation may allow pathways to maximize both sensitivity and amplification. Consistent with this possibility, some receptor proteins have recently been found to undergo LLPS via co-condensation of their non-DFDs with PAMPs/DAMPs.^112,113^ Whether receptor LLPS will prove to be the rule or the exception remains to be determined.

### Implications for aging

Hyperactive innate immune signaling is a causal hallmark of aging.^114^ Our findings suggest a simple thermodynamic basis for that relationship. The absence of a structural template appears to be the only thing that prevents self-destruction at any moment of a cell’s life. Every supersaturated protein creates an eventuality that will befall the cell if it lives long enough, whether that fate is nucleated by the intended signal or instead by an unintended but ultimately inevitable molecular fluctuation. Might DFD supersaturation therefore limit cell lifespan? As a prelude to answering that question in future work, we extended the analysis in Fig. 2B and S3C to all human cell types, and simply asked how DFD supersaturation (as approximated by the anticorrelation between DFD EC50 and expression level) relates to the mean lifespan of each cell type in the human body.^115^ From this we see that short-lived cells such as granulocytes and endothelial cells indeed appear to be more deeply supersaturated than long-lived cells such as oocytes and neurons (Figure 6B and S7).

We suspect that innate immune signaling networks have evolved to minimize the vulnerability to spontaneous self-destruction by functionalizing a minimal set of supersaturated adaptors through an expanding cadre of non-supersaturable receptors, effectors, and regulators. It is for this reason, we believe, that despite a fundamental role of self-assembly in collective DFD function, only a small fraction of human DFDs proved to be supersaturable. The extent to which those supersaturated DFDs limit longevity at both the cellular and organismal levels is now an essential question for further study.

### Limitations of the study

Multiple DFDs were found to populate stable ordered polymers in all cells. Although we did not observe a nucleation barrier for these, it is possible that they could still be supersaturable in vivo at much lower than the micromolar concentrations surveyed by DAmFRET. Similarly, a small number of DFDs that populated only a low AmFRET state did not express to high concentrations in yeast, and it is possible that they can self-assemble under cellular contexts that allow them to reach higher concentrations.

Our study necessarily simplifies and abstracts human innate immune signaling. We considered the cytoplasm of living yeast cells to be a suitable proxy for the physiological context of DFDs *as a whole*, but the exact context of endogenous DFDs will differ from that in our experiments. For example, several of the DFDs normally localize to the nucleus in human cells, and this could impact their phase behavior. Note, however, that none of these exhibited a structural nucleation barrier in our experiments, suggesting that if anything our findings may underreport the number of DFD batteries. Similarly, our work does not consider the roles of transcriptional regulation and post-translational modifications (PTMs). For example, NLRP3 is upregulated and becomes phosphorylated in response to LPS “priming” of the inflammasome,^116^ and polyubiquitination of RIG-I promotes its activation of MAVS.^117^ Such modifications imply that some signal amplification occurs upstream of supersaturated adaptors. Whether or to what extent each adaptor is supersaturated will differ between cell types and biological contexts, as a consequence of regulated expression, post-translational modifications, and binding partners. Such regulation will change how much of the corresponding battery’s capacity is used in that context.

## Materials and Methods

### Reagents and antibodies

Hygromycin B (Invivogen, ant-hg-1), Penicillin-Streptomycin (ThermoFisher, 1514014gp), PMA (BioVision, 1544–5), Puromycin (Invivogen, ant-pr-1), Sytox Orange (ThermoFisher, S11368), CellTox (Promega, G8741), Annexin V Alexa568 (ThermoFisher, A13202), Annexin V Alexa488 (ThermoFisher, A13201), Incucyte^®^ Caspase-3/7 Dye (Sartorius, 4440). Antibodies, anti-ASC (Santa Cruz Biotechnology, sc-514414), anti-FADD (Sigma, 05–486), anti-Actin (Santa Cruz Biotechnology, sc-8432) were obtained from the indicated vendors.

### Plasmid construction

Yeast expression plasmids were made as previously described^43^. Briefly, we used a golden gate cloning-compatible high copy episomal vector, V08, which contains inverted BsaI sites followed by a rigid helical linker 4x(EAAAR) and mEos3.1. This vector drives the expression of proteins from a *GAL1* promoter and contains the auxotrophic marker *URA3*. The vector V12 is identical to V08 except that mEos3.1 and linker precedes rather than follows the BsaI sites, for expressing proteins with an N-terminal fusion. Inserts were ordered as GeneArt Strings (Thermo Fisher) flanked by Type IIs restriction sites for ligation between BsaI sites in V08 and V12. All other inserts were cloned into respective vectors via Gibson assembly between the promoter and respective tag. All plasmids were verified by Sanger sequencing. All expression plasmids are listed in Table S1.

Lentivirus vectors were as previously described^46^. Briefly, optogenetic constructs were cloned into pLV-EF1a-IRES-Hygro (Addgene #85134) which encodes a Hygromycin B resistance cassette. To create lentiviral vectors expressing the the optogenetic constructs fused with miRFP670nano and Cry2, the corresponding sequences of AIM2^PYD^, APAF1^CARD^, NLRC4^CARD^ were inserted via Gibson assembly into pLV-EF1a-IRES-Hygro. Finally, The doxycycline-controlled lentiviral vectors were cloned via Gibson assembly with the respective coding sequences from PYCARD, CASP9, CASP1, and mScarlet-I into pCW57.1 (Addgene #41393). All lentivirus vectors are listed in Table S3.

### Yeast strain construction

Unseeded DAmFRET experiments were conducted using strain rhy1713.^43^ To create strains expressing DFD seeds, we first transformed AseI digests of each DFD plasmid along with a plasmid expressing Cas9 and a guideRNA targeting the *URA3* markers into rhy2153. This strain contains a genomic landing pad consisting of natMX followed by the *tetO7* promoter and counterselectable *URA3* ORFs derived from *C. albicans* and *K. lactis*, and stop-μNS-mCardinal as described^46^. Successful integration of the insert replaces the *URA3* marker with the gene of interest and fuses to the protein’s C-terminus μNS-mCardinal, under the control of a doxycycline-repressible promoter. Transformants were selected for resistance to 5-FOA and validated for successful seed integration by detection of mCardinal expression using flow cytometry. The arrayed library of resulting strains was then mated to each of the rhy1713 strains expressing separate DFD-mEos3.1 fusions, by pinning each pair of strains together onto agar omnitrays containing SD-URA+NAT+Dox media. The resulting colonies were then pinned into liquid SD-URA+NAT+Dox for continued diploid selection and creation of glycerol stocks. The entire nucleating interaction screening consisted of 384 96-well plates.

### DAmFRET assay preparation and data collection

We performed DAmFRET as previously described.^43^ Briefly, single transformant yeast colonies were inoculated in 200 μl of SD-URA in a 96-well microplate well and incubated in a Heidolph Titramax platform shaker at 30°C, 1350 RPM overnight. Cells were washed with sterile water, resuspended in galactose-containing media, and allowed to continue incubating for approximately 20 hours. Microplates were then illuminated for 25 min with 320–500 nm violet light to photoconvert a fraction of mEos3 molecules from a green (516 nm) form to a red form (581 nm). At this point, cells were either used to collect microscopy data or continue the DAmFRET protocol.

For the nucleating interaction screen, glycerol stock plates were pinned into liquid SD-URA without dox and incubated for 16 hours at 30°C with 1350 RPM shaking overnight. We then resuspended cells in fresh SD-URA media and continued incubation for an additional 20 hours. After this, we resuspended cells in SGal-URA and continued incubation for 20 hours to induce protein expression. Finally, we resuspended cells in fresh SGal-URA for four hours prior to DAmFRET data collection. The library was then consolidated into 96 384-well plates.

DAmFRET data were collected on a ZE5 cell analyzer cytometer. Autofluorescence was detected with 405 nm excitation and 460/22 nm emission; SSC and FSC were detected with 488 nm excitation and 488/10 nm emission. Donor and FRET fluorescence were detected with 488 nm excitation and 425/35 nm or 593/52 nm emission, respectively. Acceptor fluorescence was detected with 561 nm excitation and 589/15 nm emission. For each well, we collected a volume of 13 μL, resulting in approximately 500,000 events per sample. Data compensation was done in the built-in tool for compensation (Everest software V1.1) on single-color controls: non-photoconverted mEos3.1 and dsRed2 (as a proxy for the red form of mEos3). For nucleating interactions, we included an additional channel for mCardinal intensity with 561 nm excitation and 670/30 nm emission.

### DAmFRET data analysis

Data were processed on FCS Express Plus 6.04.0015 software (De Novo). Events were gated for single unbudded cells by FSC vs. SSC, followed by gating of live cells with low autofluorescence and positive donor and acceptor fluorescence. With the exception of TNFRSF10A^DD^ (TRAIL-R1) (rhx2933) which failed to express with either its C- or N-terminus tagged, all expression plasmids were processed. Plots represent the distribution of AmFRET (FRET intensity/acceptor intensity) vs. Acceptor intensity (protein expression).

We then analyzed the data as previously described.^53^ Briefly, FCS files were gated using an automated R-script running in flowCore. Before gating, the forward scatter (FS00.A, FS00.W, FS00.H), side scatter (SS02.A), donor fluorescence (FL03.A), and autofluorescence (FL17.A) channels were transformed using a logicle transform in R. Single cells were gated using FS00.A vs SS02.A and FS00.H vs FS00.W. These were gated for expressing cells using FL03.A vs FL17.A. Cells falling within these gates were then exported as FCS3.0 files for further analysis.

DAmFRET histograms were divided into 64 logarithmically spaced bins across a predetermined range large enough to accommodate all data sets. The upper gate values were determined for each bin as the 99th percentile of the DAmFRET distribution in that bin. We used the expression of mEos3.1 alone to delineate the region of a DAmFRET plot that corresponds to no assembly. For all samples, cells falling above this region are considered to contain protein assemblies (FRET-positive). The fraction of cells in the assembled population was plotted as a ratio to the total cells in the bin for all 64 bins. The gross fraction of such cells expressing a given protein is reported as fgate.

### Determination of continuity

We initially attempted to classify each DAmFRET dataset as one-state or two-state, and discontinuous or continuous for the latter, using an algorithm previously developed for this purpose.^54^ However, the algorithm invariably misclassified discontinuous datasets as “continuous” when the AmFRET level of the high FRET state changed with concentration, i.e. exhibited positive or negative slope as for FAS^DD^ and CASP2^CARD^, respectively. Due to this limitation, we adopted a different method.

To determine the continuity of an adequately expressed plasmid, we analyzed the distribution of the AmFRET values about the transition point. To do so, we fit a spline to the median AmFRET values across binned concentrations ranging from the 1st to 99th percentile of concentration values. The starting point of the spline was determined as the first bin with at least 1000 cells that reached at least the 20th percentile of all bin densities, as measured by the number of events divided by the interquartile range (IQR) of AmFRET values within the bin. The number of bins was determined using the Freedman-Diaconis rule. The median AmFRET values were calculated for each bin, and then denoised using the python scipy.signal package, resulting in the spline fit. The transition point was then defined as the maximum of the first derivative of the fitted spline, using the numpy.diff package. We then used Hartigan & Hartigan’s dip test for unimodality to determine the continuity on a narrow window of +/− 12% of the expression range around the transition point. The width of the window was chosen to account for the variability in and guided by the distribution of concentration values across the transition point of robustly self-seeded samples. We classified each plot as “discontinuous” or “continuous” for p values less than or greater than 0.05, respectively. The latter were further classified by KMeans clustering into three categories, wherein AmFRET values were either uniformly low (“low”), uniformly high (“high”), or transitioned from low to high with increasing concentration (“low to high”). We derived clustering parameters from the spline fit, encompassing overall change, maximum consecutive change, minimum, and end AmFRET values, along with the fraction of positive AmFRET. We transformed the fraction positive AmFRET data into log10 and also calculated the cube root of the ending AmFRET values. We standardized all parameters, giving twice the weight to the fraction positive, minimum, and ending AmFRET values compared to other parameters.

### Calculating centrality measures

To determine betweenness and degree of centrality, we extracted interactions involving DFD-containing proteins (listed in Table S1) from STRING version 12.0, considering only physical interactions with scores of 900 or higher. Using NetworkX v3.1, we analyzed these interactions as an undirected graph to calculate betweenness and degree of centrality. For proteins with multiple DFDs, we classified a protein as discontinuous if any of its DFDs were identified as discontinuous.

### Determination of positive nucleating interactions

We first excluded files with fewer than 2500 events positive for mEos3 or mean acceptor intensities less than 3.5 AU. From this, any DFD or seed left with less than 25% of their original instances after filtering were removed from the analysis completely. Next, we identified nucleating interactions as DFD pairs that decreased the EC50 and increased the fraction assembled (fgate). We standardized all variables for each experimental batch of DFDs to a mean of 0 and variance of 1. We then determined the outlier degree for EC50 and fgate based on the number of interquartile ranges below or above the median for these values. This was done directionally on a per-DFD basis. We defined the “nucleating interactions” for a given mEos3-fused DFD as those whose mean of these two values (reported as “seedability”) is greater than or equal to 3 standard deviations above the mean of all seedability values. We confirmed that the seedability values for most DFD pairs partitioned with that of two negative controls included for each DFD.

To evaluate the reproducibility of our assay, we replicated it for a set of 36 DFDs. This replicate analysis mirrored the original, except it utilized the DFD distributions and cutoff values from the first set. The Pearson correlation (R) between the two sets was 0.91 (p < 0.0001). To minimize the impact of random variations in negatives and outliers, we excluded double negative instances, resulting in a slightly altered Pearson correlation (R) of 0.90 (p < 0.0001). Of the 3478 DFD + seed combinations reassessed, 17 showed inconsistent hit-calling, indicating an assay consistency rate of 99.51% with a 95% confidence interval ranging from 99.28% to 99.74%.

### Approximating saturating concentrations

To generate an average DAmFRET curve, we computed the mean of each histogram bin in the DAmFRET dataset, focusing on bins containing a minimum of 100 cells. The average DAmFRET curve for each DFD in the presence of its self-seed was fit to a Weibull function as follows. We first calculated the average AmFRET value in each concentration bin. The resulting curves resemble the fraction-assembled curves except that the asymptote is the maximum AmFRET value rather than 1. Therefore, we used the following equation for fitting:

(Eq. 1) $$◻◻◻◻◻◻(◻)=◻◻◻[1-◻◻◻(-◻◻(2){\left(\frac{◻}{◻◻50}\right)}^{◻})]$$

Here Amp is the AmFRET asymptotic value of the curve, c is the concentration, EC50 is the concentration at which the curve has reached 50% of its asymptotic value, and ◻ is related to the steepness of the stretched exponential. Initial values of the parameters were chosen based on Gaussian smoothed versions of the curves and constrained in the fit to at minimum a 2-fold change from those initial guesses. The ◻ parameter was constrained between 0.1 and 10 based on expected reasonable values of that parameter. At low and high concentration values, the average AmFRET values are clearly unstable and influenced by noise and minor compensation errors. Therefore, we chose the beginning and ending points of each curve by visual inspection, choosing starting points where the curve begins to increase and ending points where the curve levels off. Error values were determined from Monte Carlo simulations as in the fitting of fraction assembly.

### Approximating supersaturability

To approximate the supersaturability of DFDs, the average EC50 of seeds that were deemed to have a nucleating interaction were compared to the average of those that did not, for each DFD, as a ratio. Only EC50s from accurate Weibull fits were used for this analysis, as determined by their requiring 10 or fewer iterations for the function to be fit. The median fold change reduction for discontinuous DFDs was found to be 6.0078, with a range of 3.6010 to 14.0324. The fold change reduction in EC50 for discontinuous DFDs was compared to continuous DFDs and found to be significantly higher. Mann Whitney U=1.0 (p=0.0004), n_continuous_ = 9, n_discontinuous_ = 10. The continuous DFDs that exhibited positive seeding interactions were either fibrillar or diffuse, not punctate as characterized in Figure 1A, when imaged in the absence of seeding. This suggests that they likely form ordered polymers, much like the discontinuous DFD counterparts.

### Cell culture

HEK293T cells and THP-1 cells were purchased from ATCC. THP-1 *PYCARD*-KO (thp-koascz) cells were purchased from InvivoGen. HEK293T cells were grown in Dulbecco’s Modified Eagle’s Medium (DMEM) with L-glutamine, 10% fetal bovine serum (FBS), and PenStrep 100U/mL. THP-1 cells were grown in Roswell Park Memorial Institute (RPMI) medium 1640 with L-glutamine and 10% FBS. All cells were grown at 37°C in a 5% CO2 atmosphere incubator. Cell lines were regularly tested for mycoplasma using the Universal mycoplasma detection kit (ATCC, #30–1012K).

### Generation of stable cell lines

Stable cell lines were created as described.^46^ Briefly, constructs were packaged into lentivirus in a 10 cm plate 60% confluent of HEK293T cells using the TransIT-LT1 (Mirus Bio, MIR2300) transfection reagent and 7 μg of the vector, 7 μg psPAX2, and 1 μg pVSV-G. Lentivirus was harvested and incubated with 293T with polybrene or infected at 1000x g for 1 hour for THP-1 cells. For transduction of pCW57.1 derived vectors, HEK293T and THP-1 cells were selected with Puromycin (1 μg/mL) for 7 days. After this time, cells were sorted for positive expression of mScarlet and expanded in continuing selection with puromycin. For transduction of plasmids encoding fusions to miRFP670nano-Cryclust, THP-1 and HEK293T cells were selected with Hygromycin B (350 μg/mL and 150 μg/ml, respectively) for 7 days. Cells were sorted for positive expression of miRFP670nano and expanded for further experiments with continued selection. To generate THP-1 PYCARD-KO + FADD-KO cells, sgRNA targeting FADD exon1 was cloned into the lentiCRISPR v2-Blast (Addgene #83480). This vector was packaged into lentivirus as described above. THP-1 PYCARD-KO cells were transduced using spinfection and supplemented with polybrene. 24 hr after spinfection, media was replaced. 48 hr after spinfection, cells were selected with blasticidin (1 μg/ml). After 10 days of blasticidin selection, single-cell clonal expansion was done by serial dilution of resistant cells to achieve complete knockouts. Selected wells were analyzed by immunoblot to confirm the absence of FADD protein and sequence-verified.

### High-content imaging analysis

High-content imaging was performed on the Opera Phenix high-content screening system (PerkinElmer) using a 63x water immersion objective. Briefly, yeast (rhy2977) transformed with individual plasmids were cultured and induced as for DAmFRET assays. Then, 10 μl were transferred into a well containing 90 μl of SGal-URA of a 96-well optically clear flat-bottom plate (PerkinElmer 6055302). Data analysis of the high content imaging was performed in Fiji. Images of mEos3 were acquired using 488 nm excitation and a standard GFP filter set. Small z stacks were acquired over 5 μm total range with 1 μm steps. The image containing the brightest mEos3 signal was used. The mEos3 signal was then background-subtracted with a rolling ball radius of 100 pixels then found and converted to Fiji ROIs using the Fiji Default method of traditional image thresholding. The mean, standard deviation, and aspect ratio (AR) were measured for each object. The coefficient of variation (CV) in pixel intensity was calculated for every object following the formula: CV = Std Dev/Mean*100. The wells were divided into 3 categories based on their AR and CV. Objects that had a CV > 55 and an AR > 1.159 were designated “fibrillar”. Objects that had a CV > 55 and an AR < 1.16 were designated “punctate”. Finally, objects that had a CV < 55 and an AR < 1.16 were designated “diffuse”. These cutoffs were determined manually from a visual inspection of the data. The results were then manually verified for all wells. We found inconsistent classification by these cutoffs for plasmids (rhx2935, rhx2637, rhx0989) due to heterogeneity in the structures formed. We also detected three DFDs with anomalously high AR (rhx1113, rhx1097, rhx2937) due to their low expression. Plasmids rhx4763 - rhx4767 were acquired in a second data set that used different laser powers and integration times. Hence, for these plasmids specifically, “fibrillar” had a CV > 17 and an AR > 1.4, “punctate” had a CV > 17 and an AR < 1.41, and “diffuse” had a CV < 18 and an AR < 1.41. These cutoffs were determined manually from a visual inspection of the data. The results were then manually verified for several wells.

### Fluorescence microscopy and optogenetic activation

The yeast and mammalian cells were imaged in an LSM 780 microscope with a 63x Plan-Apochromat (NA = 1.40) objective. T-Sapphire was excited with a 405 nm laser. mEos3 and mScarlet-I were excited with a 488 nm and 561 nm laser, respectively. For time-lapse imaging, samples were maintained at 37 °C and 5% CO2 with a stage top incubator. To stimulate Cry2clust we used the 488 nm laser at a power setting of 50% for a pulse of 10 seconds, which is the amount of time it took to scan the user-generated region of interest unless indicated otherwise. 561 and 633 nm lasers were used for imaging mScarlet-I and miRFP670nano, respectively. Pyroptosis events were tracked by incorporating the Sytox Orange reagent into the cell. To quantify the CV, images were subjected to an in-house Fiji adapted implementation of Cellpose^118^ for cellular segmentation. The Cellpose-generated regions of interest (ROIs) were used to measure specified imaging channels.

For quantification of cell dead events using IncuCyte (Sartorius), THP-1 cells were plated on a 24 or 96 well plate at a density of 4×10^8^/well or 1×10^8^/well, respectively, with PMA (10 ng/mL) for 16 hrs. Media was replaced with fresh media supplemented with Annexin V-Alexa488 and Sytox Orange. For AIM2-Cry2clust activation, an initial collection of unexposed measurements was taken for 30 min. Then, the plate was exposed to 488 nm laser every 5 min. For treatments with poly(dA:dT), cells were treated and immediately subjected to imaging every 30 minutes for 19 hours. Positive cells for either fluorophore were indentified using the integrated software in the IncuCyte instrument.

For optogenetic activation of APAF1^CARD^ and NCLR4^CARD^, HEK293T cells expressing lentivirus constructs were seeded on a 35-mm dish (ibidi) at a density of 4×10^4^/mL with 2 mL of media. The next day, Dox was added at a concentration of 1 μg/mL to induce the expression of mScarlet tagged proteins. Twenty-four hours after protein induction, media was replaced with fresh media supplemented with Incucyte^®^ Caspase-3/7 Dye (4440) 2 hours prior to the experiment or Annexin V-Alexa488. Cells were imaged using a spinning-disk confocal microscope (Nikon, CSU-W1) with a ×60 Plan Apochromat objective (NA = 1.40) and a Flash 4 sCMOS camera (Hamamatsu). A region of interest (ROI) was selected to induce optogenetic activation for indicated times using a 488 nm laser at 50% laser power for the indicated time ranging from a fraction of a second to 30 seconds. ROIs were generated by Cellpose segmentation algorithm around each cell contour. These ROIs were then used to measure the area, mean, standard deviation, and integrated density of each cell on the 488 nm and 560 nm fluorescence channels.

### Protein immunodetection

We performed capillary based protein immunodetection (Wes, Simple Protein) as described.^46^ Briefly, protein lysates were prepared as per recommended manufacturer instructions to a final concentration of 1 μg/ml. An assay plate was filled with samples, blocking reagent, primary antibodies (1:50 dilution for anti-Actin, 1:200 dilution for anti PYCARD), HRP-conjugated secondary antibodies and chemiluminescent substrate. The plate was subjected to electrophoretic protein separation and immunodetection in the fully automated capillary system. The rulting data was processed using the open-source software Compass (https://www.proteinsimple.com/compass/downloads/) to extract the intensities for the peaks corresponding to the expected molecular weight of proteins of interest. For Western Blot, cells were centrifuged at 1000 × g for 5 min and resuspended in lysis buffer (50 mM Tris (pH 7.4), 137 mM NaCl, 1 mM EDTA, 1% Triton X-100, 10 mM DTT (dithiothreitol), cOmplete protease inhibitor (1 tablet / 10 ml) (Roche, 11697498001). Protein lysates were resolved on a NuPAGE^™^ 4 to 12%, Bis-Tris gel and transferred onto a PVDF membrane (IPVH00010, Millipore) using the Pierce Power Blotter (ThermoFisher). The membrane was blocked with 5% skim milk and incubated overnight with the antibodies: anti-Actin (1:1000, sc-8432), anti-FADD (1:500, 05–486) and anti-PYCARD (1:1000, sc-514414). Primary antibody was removed by several washes with TBS + 0.1% Tween-20 and then subjected to incubation with secondary antibody (anti-mouse-HRP, 7076S, Cell Signaling Technology). The detection of protein bands was then carried out using enhanced chemiluminescence (ECL) (SuperSignal West Pico Chemiluminescent Substrate, ThermoFisher, 34577). The chemiluminescent signal was acquired by placing the membrane in a film cassette and exposing it to X-ray film (Kodak) at varying durations in a darkroom. After exposure, films were developed using an automatic film processor.

### Semi-Denaturating Detergent Agarose Gel Electrophoresis (SDD-AGE)

SDD-AGE was performed as previously described.^43^ Briefly, cells were lysed using a 2010 Geno/Grinder with bead-beating. Samples were prepared with 2% sarkosyl and separated in a 1.5% agarose gel with 0.1% SDS. The distribution of mEos3-fused proteins was analyzed directly in the gel with a GE Typhoon Imaging System. Images were processed to remove background using a 250-pixel rolling ball, cropped, and contrast-adjusted.

### Quantification and statistical analysis

Two-sided Student’s t-tests were used for significance testing unless stated otherwise for two sample comparisons. The graphs represent the means ± SEM of independent biological experiments unless stated otherwise. Statistical analysis was performed using GraphPad Prism 9, Python and R packages.

## Supplementary Material

Supplement 1 Table S1. Metadata, construct specifics, and data for the human DFDs analyzed in this study, related to Figures 1 and 2.
ND (not determined). NA (not applicable/available). FL (full-length). arhx1052 was found to be discontinuous in 1 of 4 independent experiments. brhx1053 was found to be discontinuous in 1 of 3 independent experiments. cOur results suggest that this domain of DR6 be re-annotated as DED.

Supplement 2 Table S2. Metadata, construct specifics, and data for additional proteins analyzed in this study, related to Figure 2.
FL (full-length). aMutations were made to inactivate enzymatic activity for cell death effectors.

Supplement 3 Table S3. Specifics of lentivirus vectors used in this study, related to Figures 4 and 5.

Supplement 4 Table S4. **Nucleating interactome of DFDs, related to** Figure 5. Note that plasmids rhx2938, rhx1071, rhx2934, rhx1113, rhx1064, rhx2933, and rhx1368; and seeds from rhx2938, rhx2933 and rhx2934; did not pass quality control as outlined in methods and were therefore omitted from the analysis.

Supplement 5

## Figures and Tables

**Key Resources Table:** 

Reagent type (species) or resource	Designation	Source or reference	Identifiers	Additional information
*S. cerevisiae*, strain background: S288c	rhy1713	PMID: 29979963		
*S. cerevisiae*, strain background: S288c	rhy2153	PMID: 35727133		
*S. cerevisiae*, strain background: S288c	rhy2977	PMID: 36920097		
Cell line (*H. sapiens*)	HEK293T	American Type Culture Collection	# CRL-3216	
Cell line (*H. sapiens*)	THP-1	American Type Culture Collection	# TIB-202	
Cell line (*H. sapiens*)	THP-1 PYCARD-KO	Invivogen	thp-koascz	
antibody	anti-Actin	Santa Cruz Biotechnolog y	sc-8432	Protein Simple (1:50); Western Blot (1:1000)
antibody	anti-PYCARD	Santa Cruz Biotechnolog y	sc-514414	Protein Simple (1:200); Western Blot (1:1000)
antibody	anti-FADD	Sigma-Aldrich	05–486	Protein Simple (1:10); Western Blot (1:500)
recombinant DNA reagent	pCW57.1-PYCARD-mScarlet	This paper		Lentiviral plasmid for expressing HsPYCARD-(4xEAAAR)-mScarlet-I from an inducible Doxycicline promoter
recombinant DNA reagent	pLV_AIM2-PYD-miRFP670-Cry2clust	This paper		Lentiviral plasmid for expressing HsAIM2(PYD)-(4xEAAAR)-miRFP670nano-Cry2clust from a constitutive promoter
commercial assay or kit	Sytox Orange	ThermoFisher	S11368	1:1000
commercial assay or kit	Annexin V Alexa568	ThermoFisher	A13202	1:200
commercial assay or kit	Annexin V Alexa488	ThermoFisher	A13201	1:200
commercial assay or kit	Incucyte^®^ Caspase-3/7 Dye	Sartorius	4440	1:1000
software, algorithm	Fiji/ImageJ	http://fiji.sc	RRID:SCR_002285	
software, algorithm	GraphPad Prism 9	https://www.graphpad.com/	RRID:SCR_002798	

## Data Availability

Original data underlying this manuscript can be accessed from the Stowers Original Data Repository at http://www.stowers.org/research/publications/libpb-2387
