# Supplementary figures and images for: Protein phase change batteries drive innate immune signaling and cell fate
Source: bioRxiv. 2025 Jun 6:2023.03.20.533581. Originally published 2023 Mar 21. Preprint. [Version 3] doi: 10.1101/2023.03.20.533581 (PMC10055258; doi:10.1101/2023.03.20.533581)

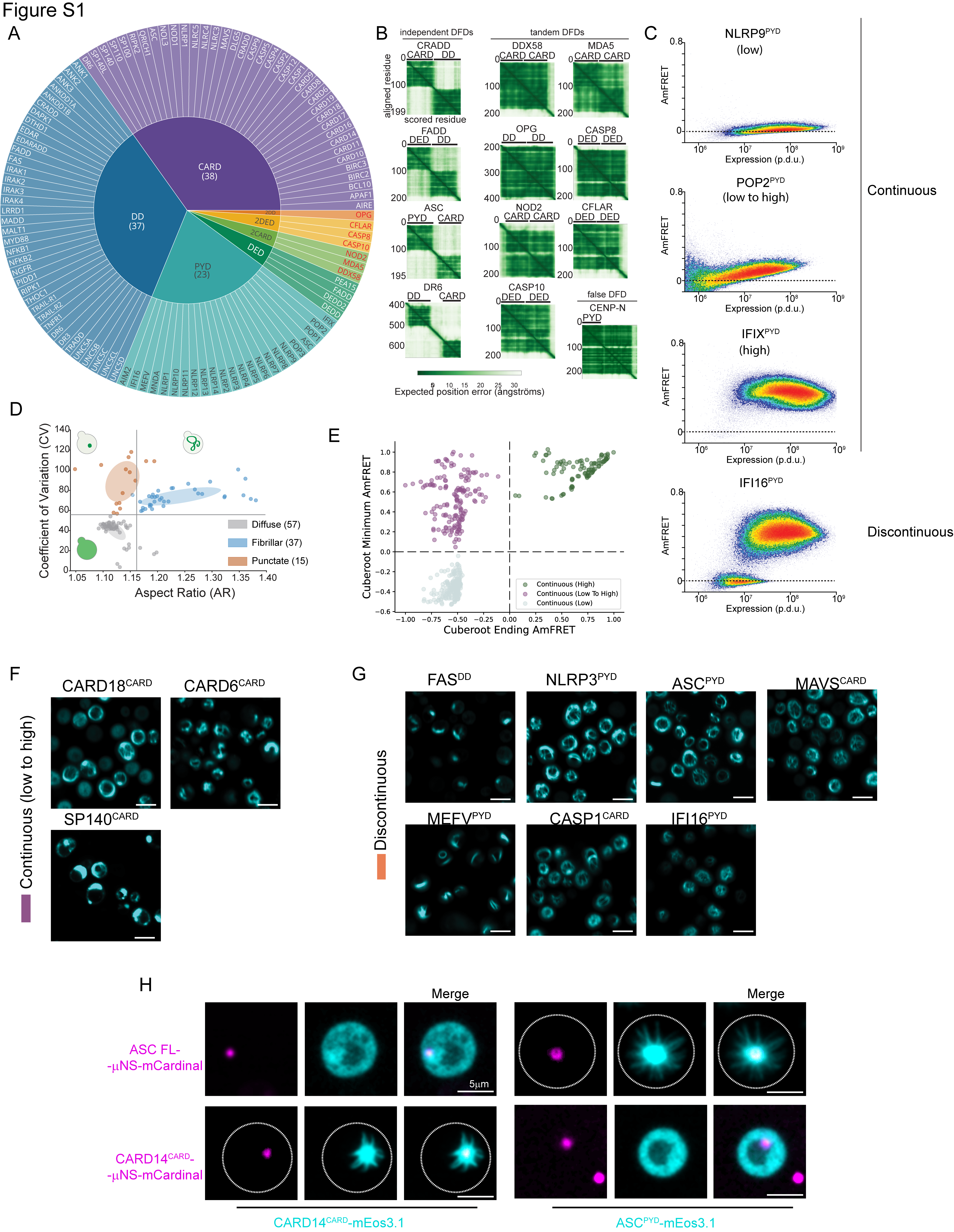

Supplement: Supplement 1 — A. Schematic diagram of all DFDs characterized in this paper, and their classification into structural subfamilies. Tandem DFDs are highlighted in red, but were analysed with their corresponding single DFD subfamilies. B. Matrices of predicted alignment error (PAE) for the indicated regions of proteins containing two DFDs, as reported in the AlphaFold Protein Structure Database,79 grouped into two categories according to interdomain PAE values consistent with either independent (left) or dependent (right) relative geometries of the DFDs. C. DAmFRET profiles of representative DFDs classified as continuous or discontinuous. D. Classification of the proteins as entirely diffuse, fibrillar or punctate based on boundaries on the scatter plot of coefficient of variation vs aspect ratio. The colored circles represent the mean and covariance of the values for each category. E. Classification of continuous DFD as “low”, “low to high” or “high” by thresholding on the minimum and ending AmFRET values of a fitted spline, normalized to that of a control DFD. F. Images of yeast expressing representative DFDs classified as fibrillar that produced continuous (low to high) DAmFRET profiles. G. Images of yeast expressing representative DFDs classified as fibrillar that produced discontinuous DAmFRET profiles. H. Representative confocal microscopy images of yeast expressing the indicated DFD constructs in the presence of the ASC or CARD14CARD seeds. The images show the emergence of filaments only from matching μNS-DFD seeds. [file media-1.tif]

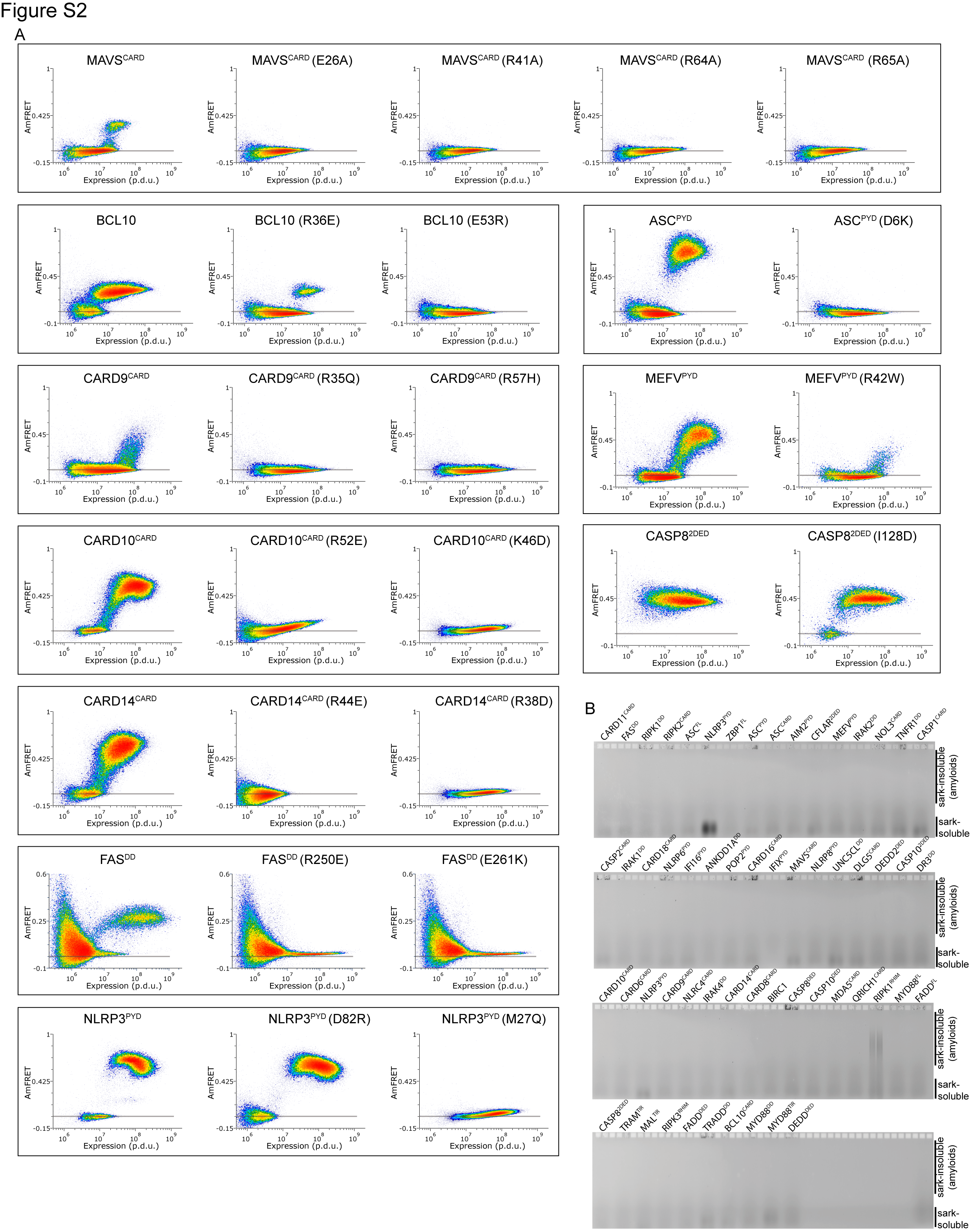

Supplement: Supplement 2 — A. Representative DAmFRET plots for the indicated DFDs with the indicated point mutations. The horizontal line approximates the mean AmFRET value for monomeric mEos3. Procedure defined units (p.d.u.). B. Image of SDD-AGE showing the size distribution of detergent-resistant multimers (where present) of mEos3-fused proteins expressed in yeast. The amyloid-forming protein, RIPK1RHIM, formed detergent-resistant multimers whereas all DFD multimers were detergent-labile. [file media-2.tif]

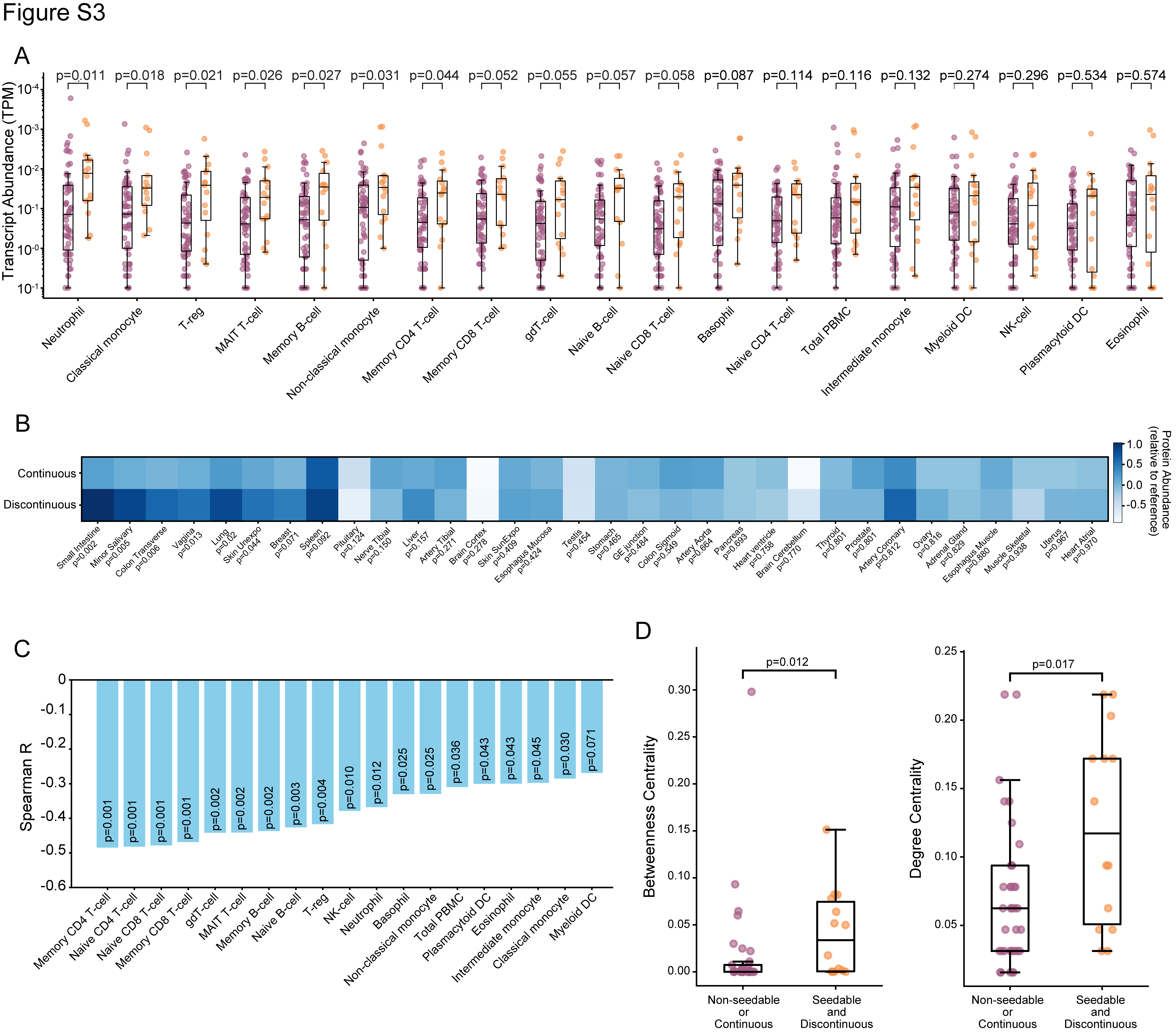

Supplement: Supplement 3 — A. Transcripts encoding proteins with discontinuous DFDs have higher expression in immune cells. P values are from Mann-Whitney test (see also Table S5). Transcripts per million (TPM) values are from the immune cell data of the Human Protein Atlas, comprising 18 cell types and total Peripheral Blood Mononuclear Cells (PBMC). B. Heatmap of protein abundance relative to reference, of discontinuous and continuous DFD containing proteins for the indicated tissues. Tissues are ordered by significance. P values are from Mann-Whitney test (see also Table S5). Protein abundance values are from the Proteome Map of the Human Body.20 C. Bar plot of the Spearman R correlation between immune cell type transcript abundance and Csat values of DFDs shows consistent negative and significant anticorrelation among immune cell types. Data were obtained from the immune cell section of the Human Protein Atlas. D. Boxplot comparing the betweenness (left) and degree centrality (right) of DFD-containing proteins that are either non-seedable or continuous (n = 37) to those that are both seedable and discontinuous (n = 14). Seedable, discontinuous proteins were found to have a significantly higher betweenness and degree centrality than non-seedable or continuous proteins. Mann-Whitney U = 145.5 (p = 0.012) and U = 146.5 (p = 0.017), respectively. [file media-3.tif]

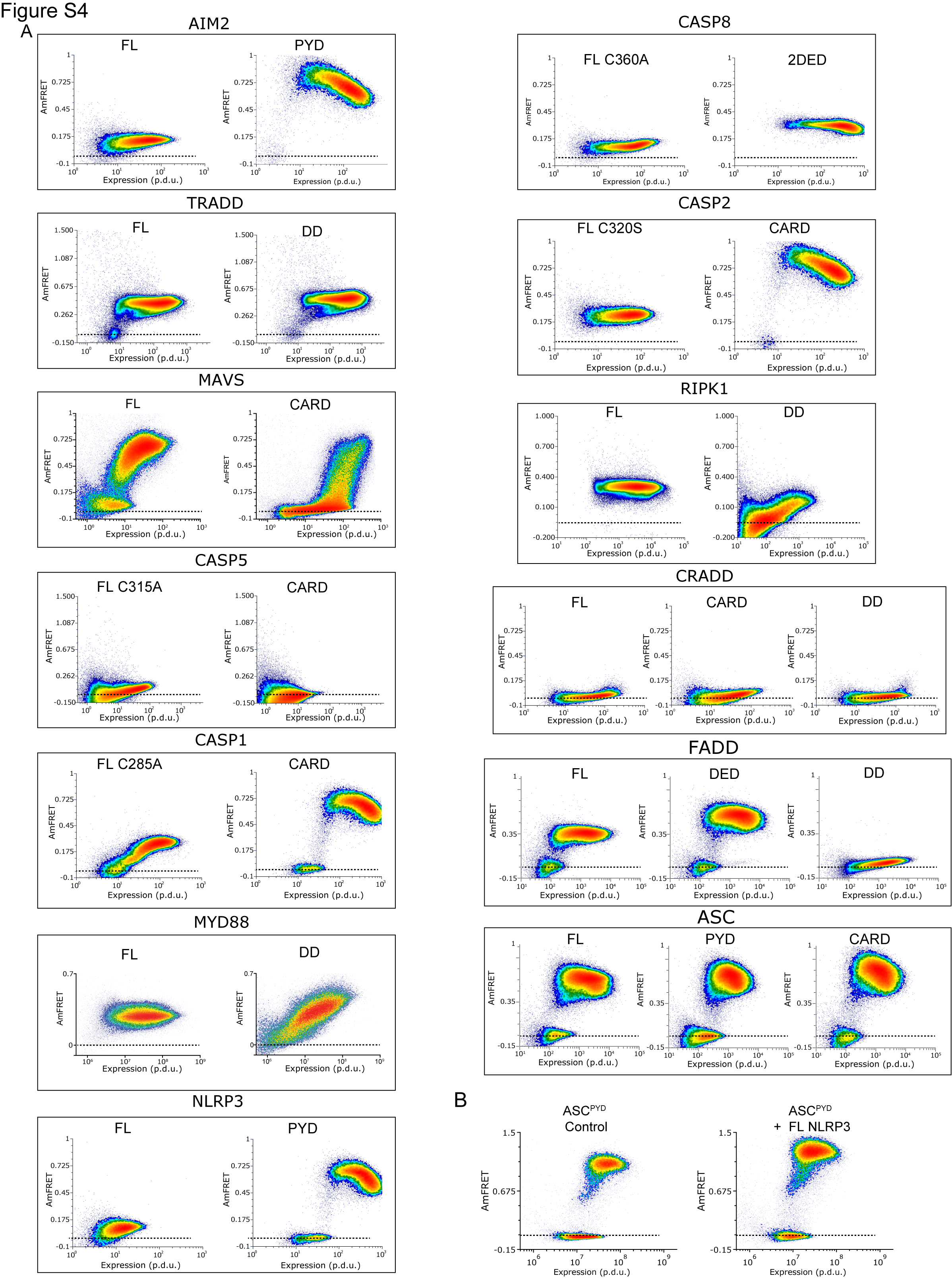

Supplement: Supplement 4 — A. Pairs of DAmFRET plots comparing the behaviours of representative DFDs and their corresponding FL proteins. Dashed horizontal lines approximate the mean AmFRET value for monomeric mEos3. FL MAVS has appreciable AmFRET in the supersaturated state that we attribute to its mitochondrial localization signal. B. Left, DAmFRET plot of ASCPYD expressed alone. Right, DAmFRET plot of ASCPYD co-expressed with FL NLRP3 showing persistence of the supersaturated bottom population indicating that FL NLRP3 oligomers are not active (in the absence of stimulation). [file media-4.tif]

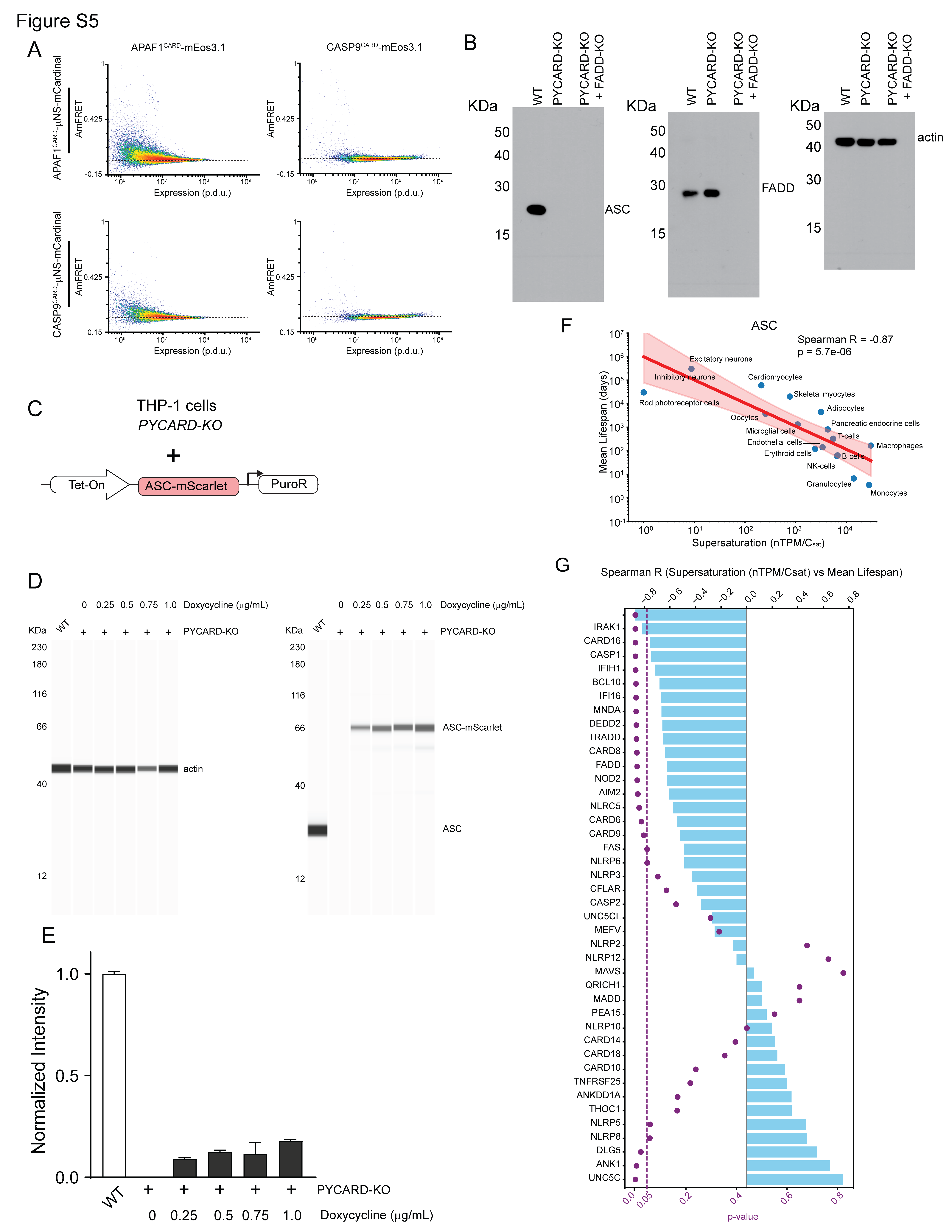

Supplement: Supplement 5 — A. DAmFRET plots of APAF1CARD and CASP9CARD measured in the presence of the indicated “seeds” expressed in trans. Both proteins fail to populate a high-AmFRET state. B. Western blot verifying the knock-out status of ASC and/or FADD in the respective engineered stable THP-1 cell lines. Actin is the loading control. C. Cartoon depicting the doxycycline-inducible ASC-mScarlet-I that replaced endogenous ASC in THP-1 PYCARD-KO cells. D. Representative capillary western blots comparing expression levels of the dox-inducible ASC-mScarlet-I construct alongside endogenous ASC. Actin is the loading control. E. Quantification of the data showing significantly lower-than endogenous levels of ASC in the engineered construct at even the highest level of induction by doxycycline. For each condition, one million cells were sorted and lysed. F. Scatter plot showing the relationship between ASC supersaturation (as approximated by the ratio of transcription levels and Csat values) and mean lifespan for each cell type as indicated in Figure 4H. The red line represents the best-fit power-law regression, obtained by performing linear regression in log-log space. The shaded region represents the 95% confidence interval for the trend line. Spearman R = −0.87 (two-tailed p = 0.0000057). G. Bar plot of the Spearman R correlation between supersaturation and cell mean lifespan of cell types as shown in Figure 4H for each DFD. Transcript levels for each cell type were obtained from the single cell RNA dataset of the Human Protein Atlas. [file media-5.tif]

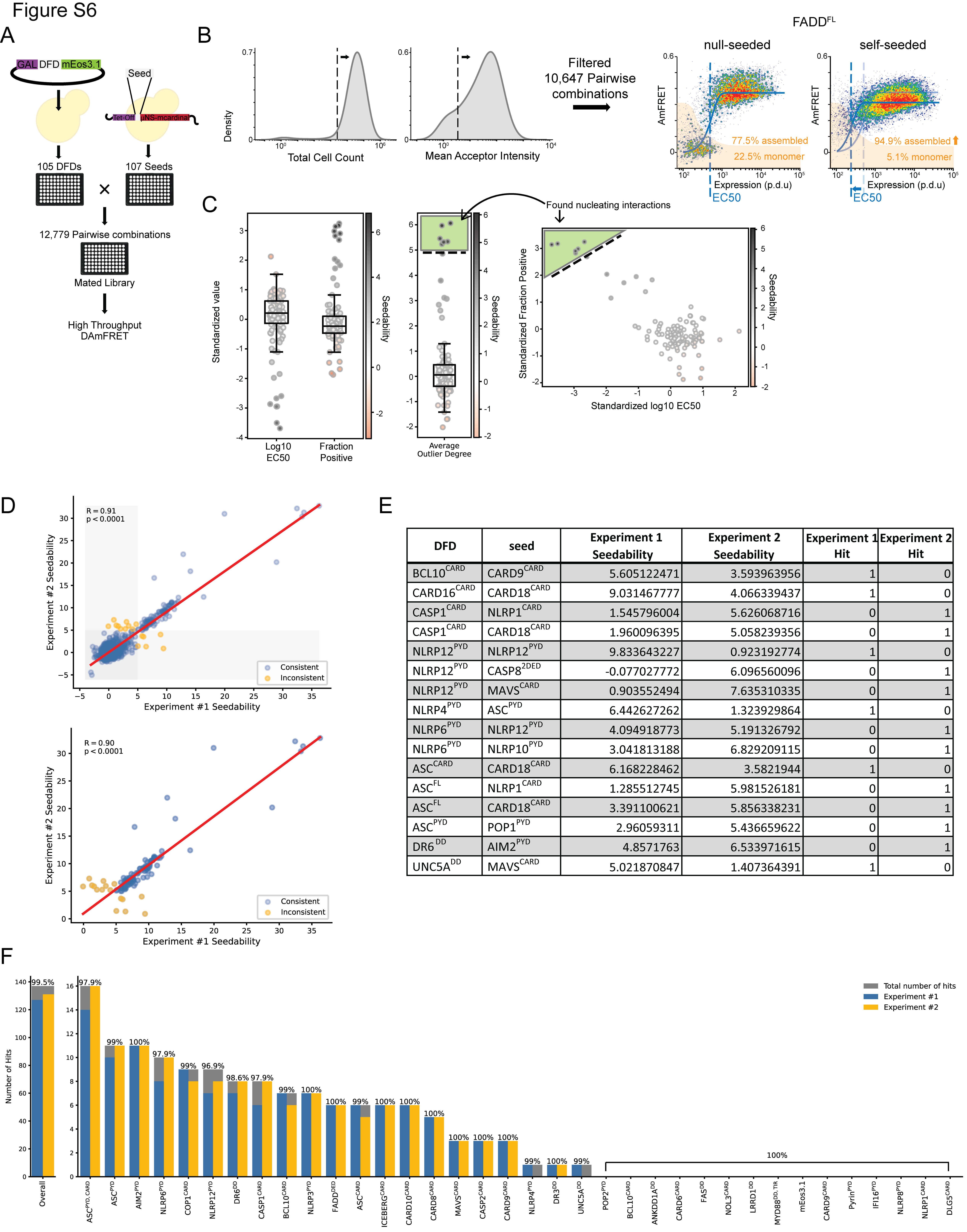

Supplement: Supplement 6 — A. Illustration of how the library of all pairs of DFDs was created. An arrayed sublibrary of yeast transformed with 105 DFD-mEos3 fusions was mated to a separate arrayed sublibrary of yeast strains expressing 107 chromosomally integrated DFD-μNS-mCardinal fusions, to create a library of 12,660 diploid strains representing all pairwise combinations. B. Left, DAmFRET was run on all pairwise combinations. Only high quality datasets -- having a total cell count greater or equal to 2500 and a mean acceptor intensity greater or equal to 3.5 p.d.u. -- were used in the analysis. Right, DAmFRET plots of FADDFL either null-seeded (lacking a DFD) or self-seeded. Nucleating interactions (as shown by the self-seeded example) are indicated by a reduced C50 and increased percentage of cells with self-assemblies (those above the gate delimiting low FRET, shown in orange). C. Hits are determined by a multiparameter combination of the degree of C50 outlier and the degree of fraction assembled outlier as defined by how many interquartile ranges (IQR) a plot is below or above the median, respectively. Points on the graph are shaded by this parameter. The leftmost set of boxplots show the distribution of standardized log10 C50 and fraction assembled for all seeds for a representative protein, FADDFL. The middle boxplot shows the average outlier degree value of these two parameters. This value is referred to as “seedability” throughout the text and is used in determining hits. The cutoff value for hit determination was set to be 3 standard deviations above the mean of all seedability values across the screen. The scatter plot on the right shows the standardized log10 C50 and fraction assembled values, depicting the contribution of both to the scoring value and positive nucleating interactions within the green box. D. Top, scatter plot of seedability values from the two replicate experiments containing 3478 DFD + seed combinations. Points are colored according to agreement between the tw [file media-6.tif]

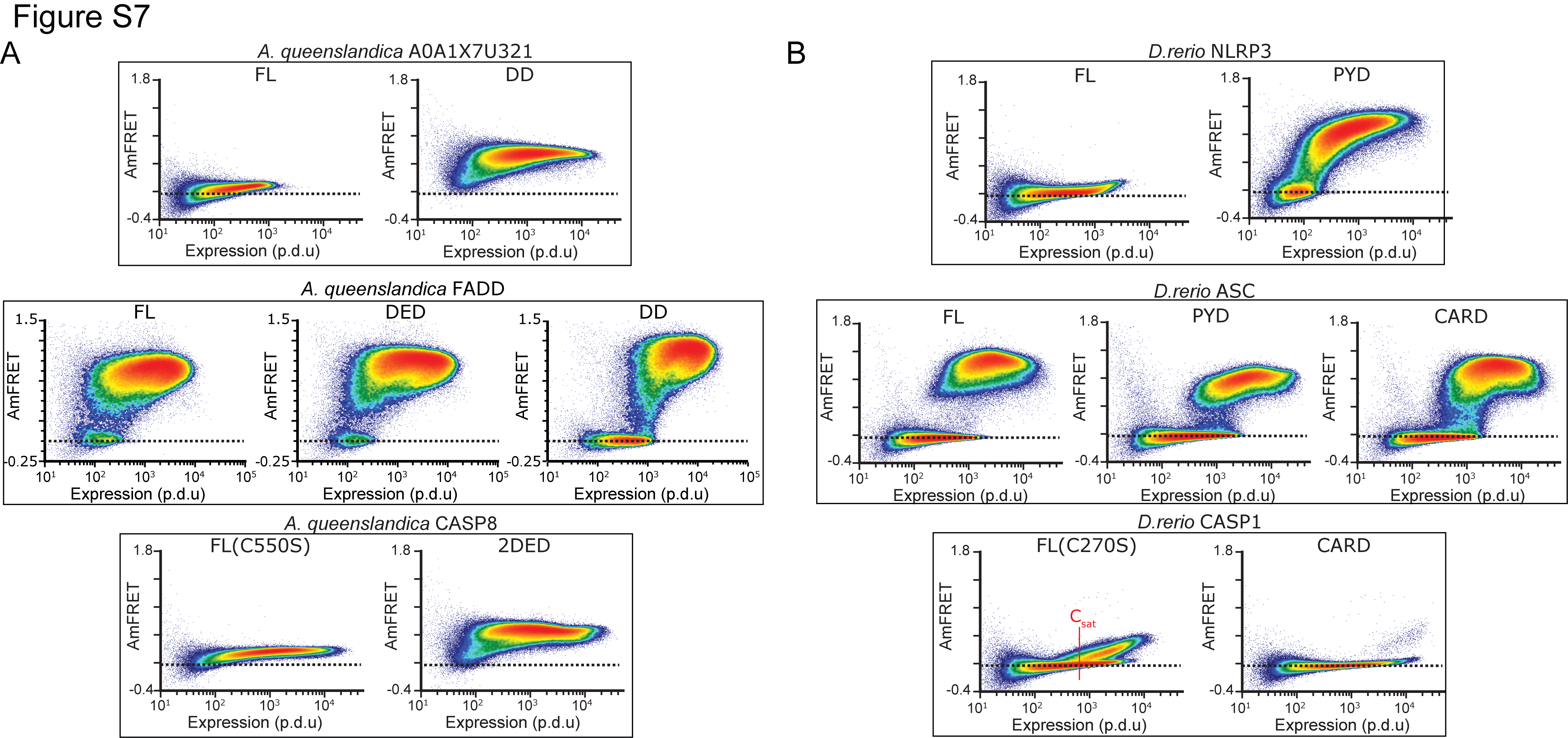

Supplement: Supplement 7 — A. DAmFRET data of DFD-only and full length inferred DISC components from the model sponge, Amphimedon queenslandica. The distant homolog to human FADD exhibits supersaturability in its FL and isolated DFDs. B. DAmFRET data of DFD-only and full length inflammasome components from the model fish Danio rerio. [file media-7.tif]
