# Supplementary material for: Protein phase change batteries drive innate immune signaling and cell fate
Source: bioRxiv. 2025 Jun 6:2023.03.20.533581. Originally published 2023 Mar 21. Preprint. [Version 3] doi: 10.1101/2023.03.20.533581 (PMC10055258; doi:10.1101/2023.03.20.533581)
Supplement: Supplement 17 [file NIHPP2023.03.20.533581v3-supplement-17.pdf]

bioRxiv preprint doi: https://doi.org/10.1101/2023.03.20.533581; this version posted June 6, 2025. The copyright holder for this preprint (which was not certified by peer review) is the author/funder, who has granted bioRxiv a license to display the preprint in perpetuity. It is made available under aCC-BY 4.0 International license.

Supplementary Materials

Supplementary text. **Experiments to investigate the nature of DFD assembly.** DAmFRET data do not clarify if self-assembly involves native DFD interactions rather than amyloid-like misfolding.[12] To address this question, we introduced point mutations to disrupt assembly via conserved known interfaces between folded DFD subunits.[16,27] Across the multiple DFDs examined, all such mutations indeed reduced or eliminated the high-AmFRET population (**Figure S2A**). To directly evaluate the nature of DFD assembly in our experiments, we subjected the seeded and unseeded cells to semi-denaturing detergent-agarose gel electrophoresis (SDD-AGE), a technique that distinguishes amyloids from other protein states based on their detergent-resistance and size dispersity.[84] We found that unlike our amyloid control (RIPK1^{RHIM}), none of the DFD assemblies survived sarkosyl exposure (**Figure S2B**), consistent with their retaining the death fold rather than misfolding into amyloid. Ongoing work is elucidating the physical basis of these nucleation barriers.
