# Supplementary material for: Protein supersaturation powers innate immune signaling
Source: bioRxiv. 2024 Mar 3:2023.03.20.533581. Originally published 2023 Mar 21. Preprint. [Version 2] doi: 10.1101/2023.03.20.533581 (PMC10055258; doi:10.1101/2023.03.20.533581)
Supplement: Supplementary file 1 [file NIHPP2023.03.20.533581v2-supplement-5.pdf]

bioRxiv preprint doi: https://doi.org/10.1101/2023.03.20.533581; this version posted March 3, 2024. The copyright holder for this preprint (which was not certified by peer review) is the author/funder, who has granted bioRxiv a license to display the preprint in perpetuity. It is made available under aCC-BY 4.0 International license.

Supplementary figure legends

Figure S1. **Sequence, imaging and DAmFRET analysis reveal diverse sequence-encoded phase behaviors of DFDs, related to Figure 1**

A. Schematic of all DFDs characterized in this paper, and their classification into structural subfamilies. Tandem DFDs are highlighted in red, but were analyzed with their corresponding single DFD subfamilies.
B. Matrices of Predicted Alignment Error (PAE) for the indicated regions of proteins containing two DFDs, as reported in the AlphaFold Protein Structure Database,[51] grouped into two categories according to interdomain PAE values consistent with either independent (left) or dependent (right) relative geometries of the DFDs.
C. DAmFRET profiles of representative DFDs classified as continuous or discontinuous.
D. Classification of the proteins as entirely diffuse, fibrillar or punctate based on boundaries on the scatter plot of coefficient of variation vs aspect ratio. The colored circles represent the mean and covariance of the values for each category.
E. Analysis showing a prominence of fibrillar morphology of DFDs that had a discontinuous DAmFRET profile (chi-square contingency test, $p<0.001$).
F. Images of yeast expressing representative DFDs classified as fibrillar that produced continuous (low to high) DAmFRET profiles.
G. Images of yeast expressing representative DFDs classified as fibrillar that produced discontinuous DAmFRET profiles.
H. Representative confocal microscopy images of yeast expressing the indicated DFD constructs in the presence of the ASC or $CARD14^{CARD}$ seeds. The images show the emergence of filaments from homotypic but not heterotypic μNS-DFD seeds.
I. Image of SDD-AGE showing the size distribution of detergent-resistant multimers (where present) of mEos3-fused proteins expressed in yeast. The amyloid-forming protein, $RIPK1^{RHIM}$, formed detergent-resistant multimers whereas all DFD multimers were detergent-labile.

Figure S2. **Self-assembly involves subunit interfaces shared with solved DFD polymer structures, related to Figure 1**

Representative DAmFRET plots for the indicated DFDs with the indicated point mutations. The horizontal line approximates the mean AmFRET value for monomeric mEos3. Procedure Defined Units (p.d.u).

Figure S3. **Proteins with DFDs that have seedable and/or discontinuous DAmFRET are central to their physical interaction networks and are more likely to be supersaturated *in vivo*, related to Figure 2**

A. Transcripts encoding proteins with discontinuous DFDs have higher expression (normalized Transcipts Per Million, nTPM) in monocytes. Mann Whitney U = 378, $n_{continuous} = 84$, $n_{discontinuous} = 15$ ($p = 0.0137$). nTPM values were extracted from the single cell RNA dataset from the Human Protein Atlas.

bioRxiv preprint doi: https://doi.org/10.1101/2023.03.20.533581; this version posted March 3, 2024. The copyright holder for this preprint (which was not certified by peer review) is the author/funder, who has granted bioRxiv a license to display the preprint in perpetuity. It is made available under aCC-BY 4.0 International license.

B. Proteins with discontinuous DFDs have higher protein abundances at the whole organism level. Mann Whitney U = 400.5, $n_{continuous}$ = 82, $n_{discontinuous}$ = 16 (p = 0.0295).
C. Bar plot of the Spearman R correlation between immune cell type transcript abundance and EC50 values of DFDs shows consistent negative and significant anticorrelation among immune cell types. Transcript data was obtained from the immune cell section of the Human Protein Atlas.
D. Boxplot comparing the betweenness (left) and degree centrality (right) of DFD-containing proteins that are either non-seedable or continuous (n = 53) to those that are both seedable and discontinuous (n = 16). Seedable, discontinuous proteins were found to have a significantly higher betweenness and degree centrality than non-seedable or continuous proteins. Mann Whitney U = 134 (p = 0.0035) and U = 160.5 (p = 0.0192), respectively.

Figure S4. **Proteins characterized as signaling adaptors display discontinuity in its DFD and FL context, related to Figure 2**

A. Pairs of DAmFRET plots comparing the behaviors of representative DFDs and their corresponding full-length proteins. Dashed horizontal lines approximate the mean AmFRET value for monomeric mEos3. FL MAVS has appreciable AmFRET in the supersaturated state due to its mitochondrial localization signal that sequesters it to internal membranes.
B. Left, DAmFRET plot of ASC^{PYD} expressed alone. Right, DAmFRET plot of ASC^{PYD} co-expressed with FL NLRP3 showing persistence of the supersaturated bottom population indicating that FL NLRP3 oligomers are not active (in the absence of stimulation).

Figure S5. **Characterization of engineered THP-1 cell lines and apoptosome assembly, related to Figures 3 and 4**

A. DAmFRET plots of $APAF1^{CARD}$ and $CASP9^{CARD}$ measured in the presence of the indicated “seeds” expressed in trans. Both proteins fail to populate a high-AmFRET state.
B. Western blot verifying the knock-out status of ASC and/or FADD in the respective engineered stable THP-1 cell lines. Actin is the loading control.
C. Cartoon depicting the doxycycline-inducible ASC-mScarlet that replaced endogenous ASC in THP-1 *PYCARD-KO* cells.
D. Representative capillary Western blots comparing expression levels of the dox-inducible ASC-mScarlet construct alongside endogenous ASC. Actin is the loading control.
E. Quantification of the data showing significantly lower-than endogenous levels of ASC in the engineered construct at even the highest level of induction by Doxycycline. For each condition, 1 million cells were sorted and lysed.

Figure S6. **Generation and validation of the DFD nucleating interactome, related to Figure 5**

A. Illustration of how the library of all pairs of DFDs was created. An arrayed sublibrary of yeast transformed with 105 DFD-mEos3 fusions was mated to a separate arrayed

bioRxiv preprint doi: https://doi.org/10.1101/2023.03.20.533581; this version posted March 3, 2024. The copyright holder for this preprint (which was not certified by peer review) is the author/funder, who has granted bioRxiv a license to display the preprint in perpetuity. It is made available under aCC-BY 4.0 International license.

sublibrary of yeast strains expressing 107 chromosomally integrated DFD-μNS-mCardinal fusions, to create a library of 12,660 diploid strains representing all pairwise combinations.

B. Left, DAmFRET was run on all pairwise combinations. Only high quality datasets -- having a total cell count greater or equal to 2500 and a mean acceptor intensity greater or equal to 3.5 p.d.u. -- were used in the analysis. Right, DAmFRET plots of $FADD^{FL}$ either null-seeded (lacking a DFD) or self-seeded. Nucleating interactions (as shown by the self-seeded example) are indicated by a reduced EC50 and increased percentage of cells with self-assemblies (those above the gate delimiting low FRET, shown in orange).
C. Hits are determined by a multiparameter combination of the degree of EC50 outlier and the degree of fraction assembled outlier as defined by how many interquartile ranges (IQR) a plot is below or above the median, respectively. Points on the graph are shaded by this parameter. The leftmost set of boxplots show the distribution of standardized log10 EC50 and fraction assembled for all seeds for a representative protein, $FADD^{FL}$. The middle boxplot shows the average outlier degree value of these two parameters. This value is referred to as “seedability” throughout the text and is used in determining hits. The cutoff value for hit determination was set to be 3 standard deviations above the mean of all seedability values across the screen. The scatter plot on the right shows the standardized log10 EC50 and fraction assembled values, depicting the contribution of both to the scoring value and positive nucleating interactions within the green box.
D. Top, scatter plot of seedability values from the two replicate experiments containing 3478 DFD + seed combinations. Points are colored according to agreement between the two experiments. Gray rectangles indicate seedability values of negative interactions, with the dark gray square containing the interaction found to be negative for both instances. Bottom, the replicates after the removal of instances found to be negative in both experiments to reduce random and outlier effects. The line of best fit is shown in red. Pearson correlation R between the two experiments = 0.91 ($p < 0.0001$). In order to reduce the effects of random variations of the negatives as well as outlier effects, we omitted double negative instances. In this case, the Pearson correlation R = 0.90 ($p < 0.0001$).
E. 17 DFD + seed combinations that had inconsistent hit-calling out of the 3478 combinations reassessed. From this we determine that our assay had a consistency of 99.51% with a 95% confidence interval of 99.28 - 99.74%.
F. Bar plots showing the number of hits per experiment, as well as total number of hits, determined for each replicated DFD. Blue and yellow indicate separate hit counts for each experiment. Gray are the number of unique hits found across both experiments. What percentage of seeds are consistently called hits are shown for each DFD. The left bar plot shows the overall summary of all DFDs included in both sets.

Figure S7. Bar plot of the Spearman R correlation between cell type transcript abundance and EC50 values of DFDs, related for Figure 6

Transcript levels for each cell type were obtained from the single cell RNA dataset of the Human Protein Atlas.

bioRxiv preprint doi: https://doi.org/10.1101/2023.03.20.533581; this version posted March 3, 2024. The copyright holder for this preprint (which was not certified by peer review) is the author/funder, who has granted bioRxiv a license to display the preprint in perpetuity. It is made available under aCC-BY 4.0 International license.

Figure S1

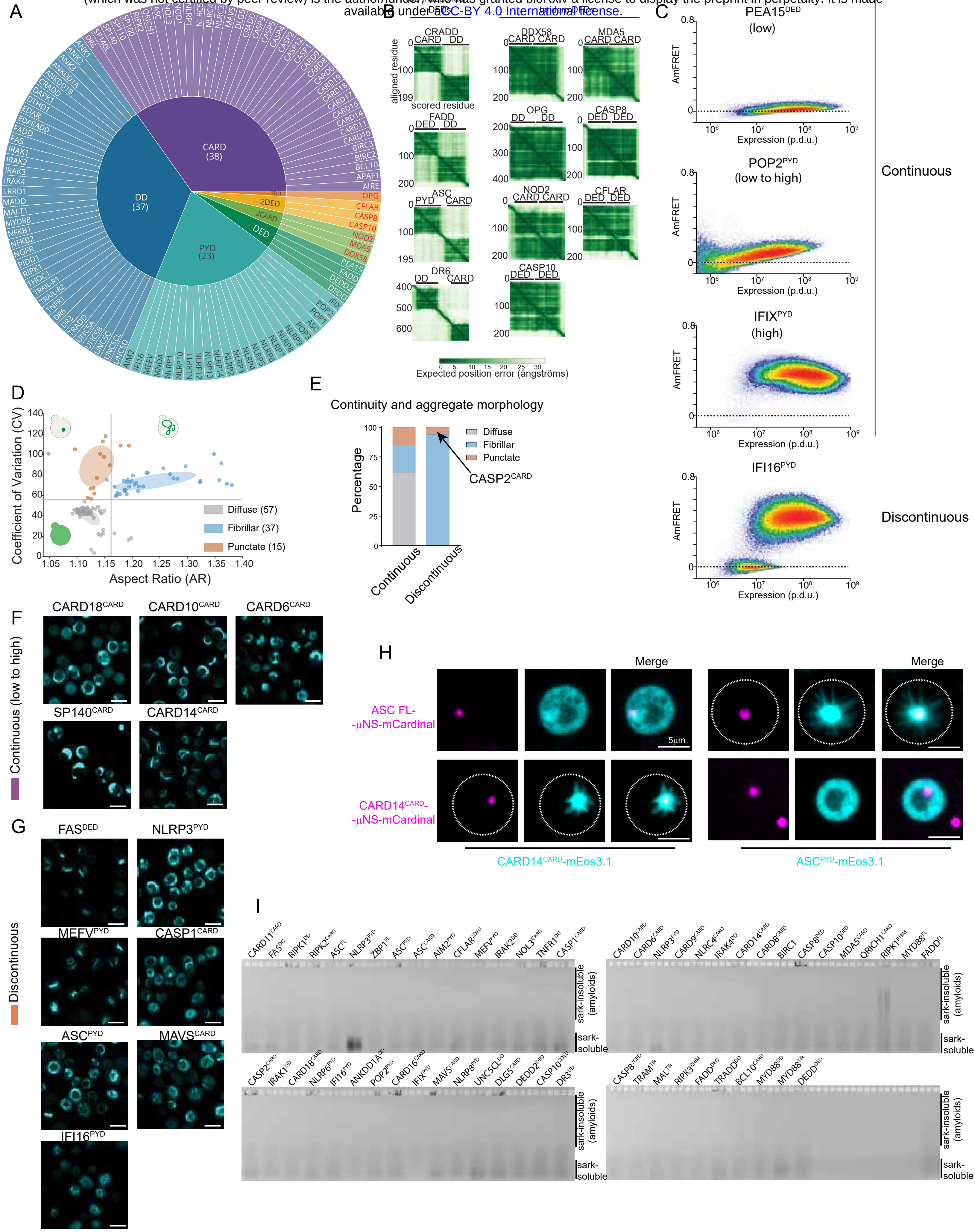

Figure S2

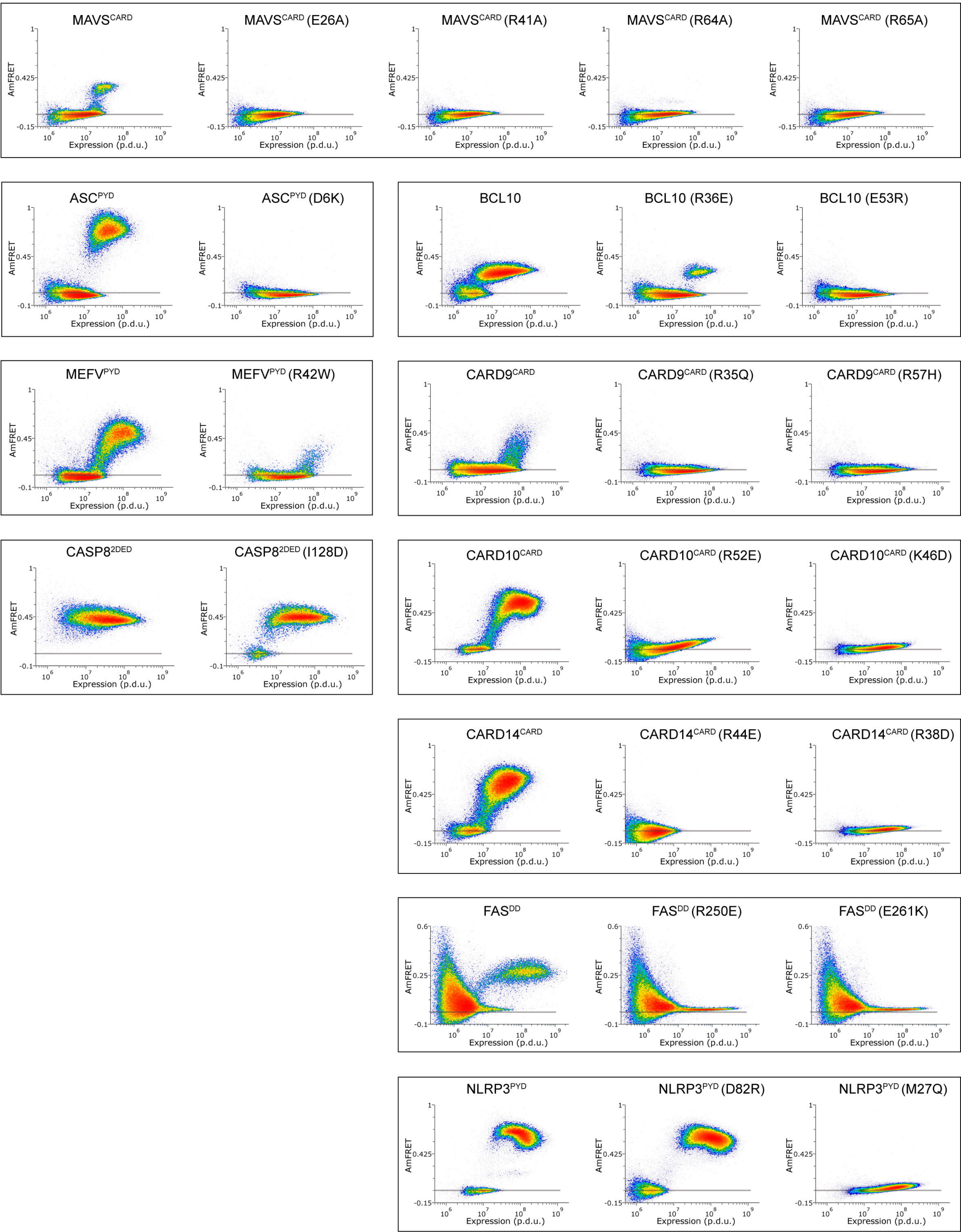
MAVS^{CARD}
MAVS^{CARD} (E26A)
MAVS^{CARD} (R41A)
MAVS^{CARD} (R64A)
MAVS^{CARD} (R65A)
ASC^{PYD}
ASC^{PYD} (D6K)
BCL10
BCL10 (R36E)
BCL10 (E53R)
MEFV^{PYD}
MEFV^{PYD} (R42W)
CARD9^{CARD}
CARD9^{CARD} (R35Q)
CARD9^{CARD} (R57H)
CASP8^{2DED}
CASP8^{2DED} (I128D)
CARD10^{CARD}
CARD10^{CARD} (R52E)
CARD10^{CARD} (K46D)
CARD14^{CARD}
CARD14^{CARD} (R44E)
CARD14^{CARD} (R38D)
FAS^{DD}
FAS^{DD} (R250E)
FAS^{DD} (E261K)
NLRP3^{PYD}
NLRP3^{PYD} (D82R)
NLRP3^{PYD} (M27Q)
AmFRET
Expression (p.d.u.)

Figure S3

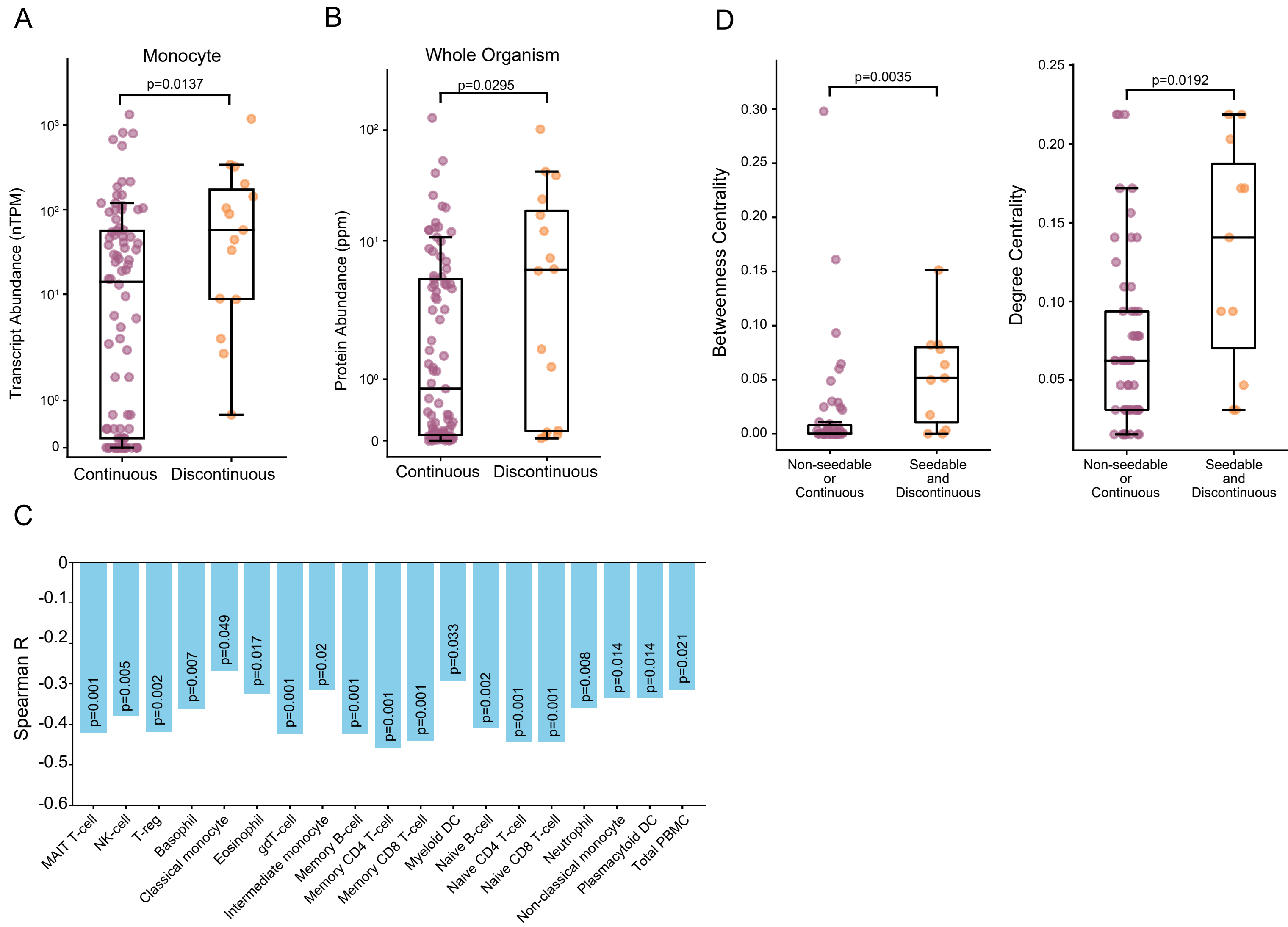
A
Monocyte
p=0.0137
Transcript Abundance (nTPM)
Continuous
Discontinuous
B
Whole Organism
p=0.0295
Protein Abundance (ppm)
Continuous
Discontinuous
D
p=0.0035
Betweenness Centrality
Non-seedable or Continuous
Seedable and Discontinuous
p=0.0192
Degree Centrality
Non-seedable or Continuous
Seedable and Discontinuous
C
Spearman R
p=0.001
p=0.005
p=0.002
p=0.007
p=0.049
p=0.017
p=0.001
p=0.02
p=0.001
p=0.001
p=0.001
p=0.033
p=0.002
p=0.001
p=0.001
p=0.008
p=0.014
p=0.014
p=0.021
MAIT T-cell
NK-cell
T-reg
Basophil
Classical monocyte
Eosinophil
gdT-cell
Intermediate monocyte
Memory B-cell
Memory CD4 T-cell
Memory CD8 T-cell
Myeloid DC
Naive B-cell
Naive CD4 T-cell
Naive CD8 T-cell
Neutrophil
Non-classical monocyte
Plasmacytoid DC
Total PBMC

bioRxiv preprint doi: https://doi.org/10.1101/2023.03.20.533581; this version posted March 3, 2024. The copyright holder for this preprint (which was not certified by peer review) is the author/funder, who has granted bioRxiv a license to display the preprint in perpetuity. It is made available under aCC-BY 4.0 International license.

Figure S4

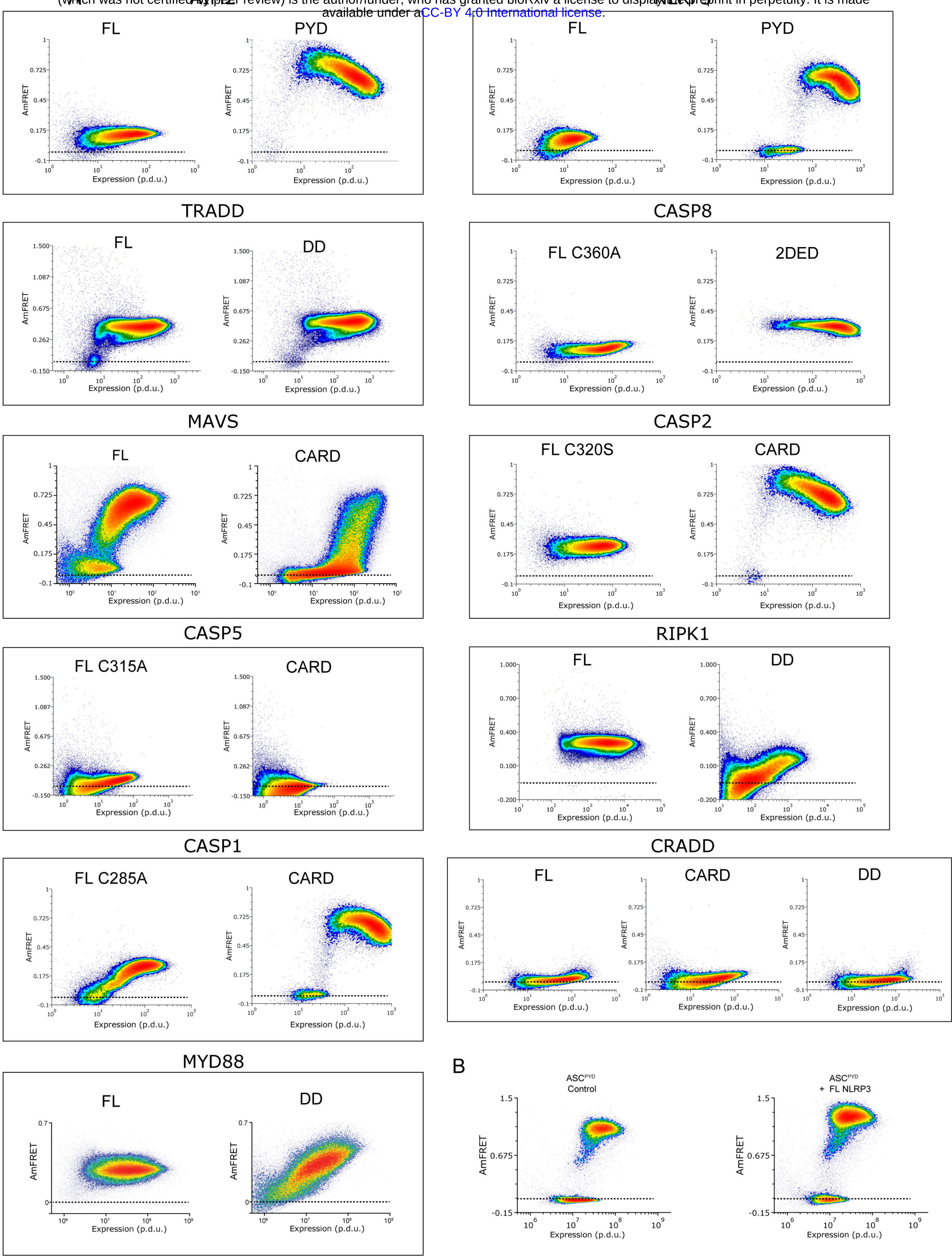

Figure S5

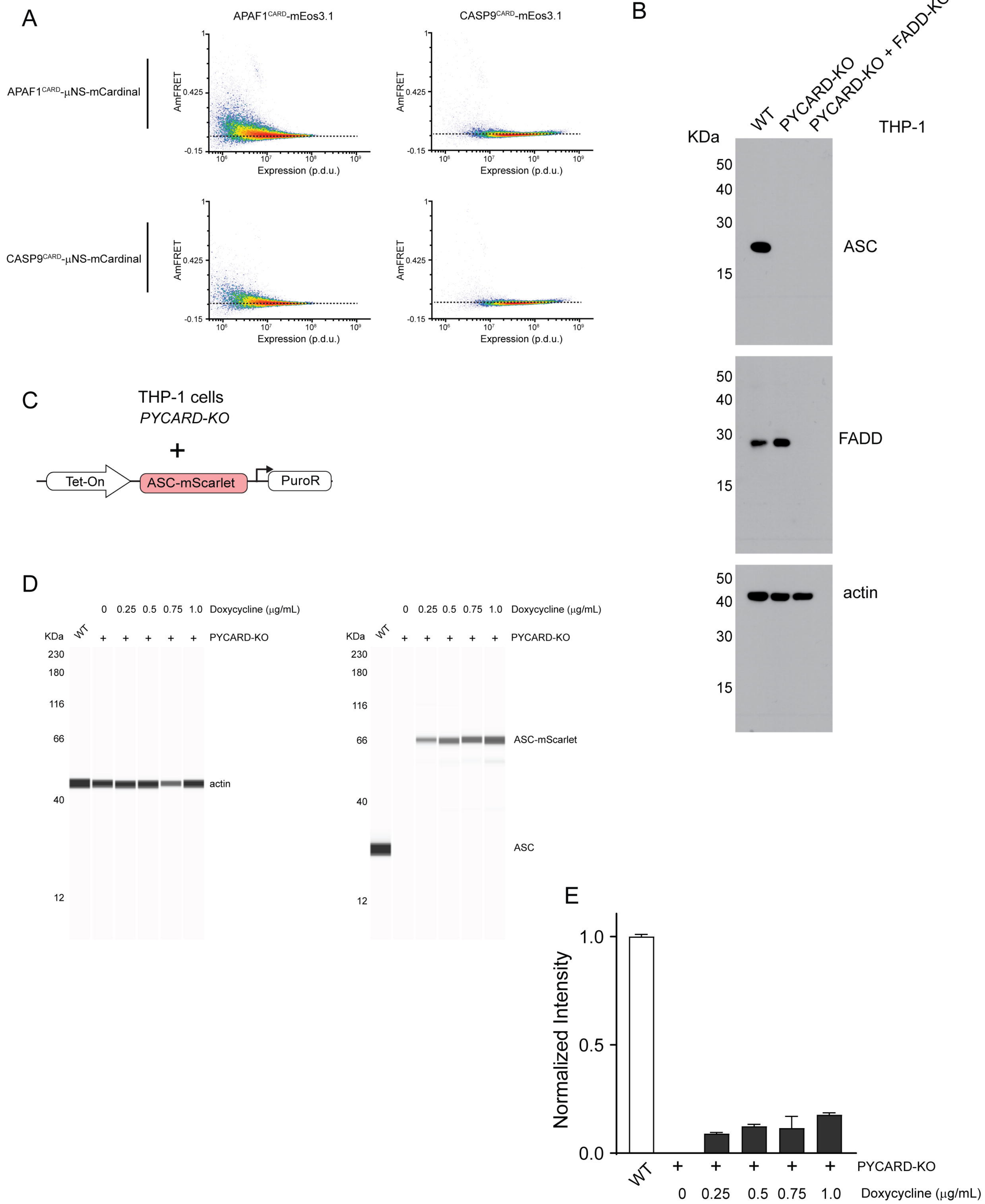

A
APAF1CARD-mEos3.1
CASP9CARD-mEos3.1
APAF1CARD-μNS-mCardinal
CASP9CARD-μNS-mCardinal
AmFRET
1
0.425
-0.15
Expression (p.d.u.)
B
WT
PYCARD-KO
PYCARD-KO + FADD-KO
THP-1
KDa
50
40
30
15
ASC
FADD
actin
C
THP-1 cells
PYCARD-KO
+
Tet-On
ASC-mScarlet
PuroR
D
0 0.25 0.5 0.75 1.0 Doxycycline (μg/mL)
KDa
WT
+ + + + + PYCARD-KO
230
180
116
66
40
12
actin
ASC-mScarlet
ASC
E
Normalized Intensity
1.0
0.5
0.0
WT
+ + + + + PYCARD-KO
0 0.25 0.5 0.75 1.0 Doxycycline (μg/mL)

Figure S6

bioRxiv preprint doi: https://doi.org/10.1101/2023.03.20.533581; this version posted March 3, 2024. The copyright holder for this preprint (which was not certified by peer review) is the author/funder, who has granted bioRxiv a license to display the preprint in perpetuity. It is made available under aCC-BY 4.0 International license.

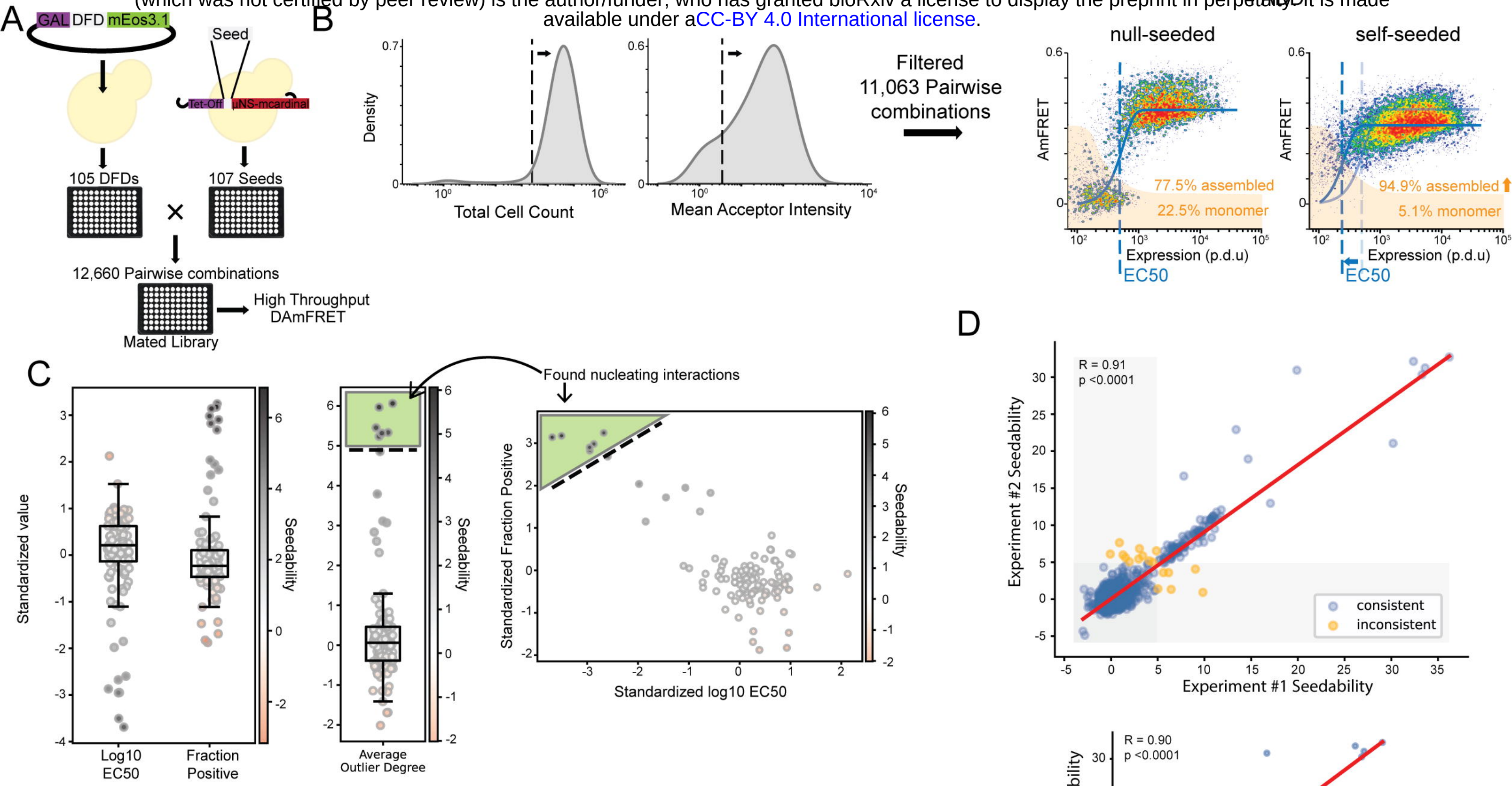

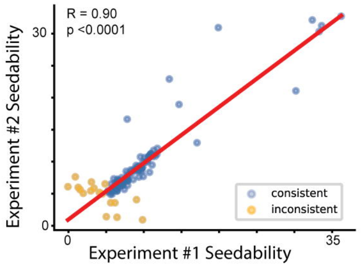

E

DFD	seed	Experiment 1 Seedability	Experiment 2 Seedability	Experiment 1 Hit	Experiment 2 Hit
BCL10^{CARD}	CARD9^{CARD}	5.605122471	3.593963956	1	0
CARD16^{CARD}	CARD18^{CARD}	9.031467777	4.066339437	1	0
CASP1^{CARD}	NLRP1^{CARD}	1.545796004	5.626068716	0	1
CASP1^{CARD}	CARD18^{CARD}	1.960096395	5.058239356	0	1
NLRP12^{PYD}	NLRP12^{PYD}	9.833643227	0.923192774	1	0
NLRP12^{PYD}	CASP8^{2DED}	-0.077027772	6.096560096	0	1
NLRP12^{PYD}	MAVS^{CARD}	0.903552494	7.635310335	0	1
NLRP4^{PYD}	ASC^{PYD}	6.442627262	1.323929864	1	0
NLRP6^{PYD}	NLRP12^{PYD}	4.094918773	5.191326792	0	1
NLRP6^{PYD}	NLRP10^{PYD}	3.041813188	6.829209115	0	1
ASC^{CARD}	CARD18^{CARD}	6.168228462	3.5821944	1	0
ASC^{FL}	NLRP1^{CARD}	1.285512745	5.981526181	0	1
ASC^{FL}	CARD18^{CARD}	3.391100621	5.856338231	0	1
ASC^{PYD}	POP1^{PYD}	2.96059311	5.436659622	0	1
DR6^{DD}	AIM2^{PYD}	4.8571763	6.533971615	0	1
DR6^{DD}	NLRP6^{PYD}	4.493927648	4.971884105	0	1
UNC5A^{DD}	MAVS^{CARD}	5.021870847	1.407364391	1	0

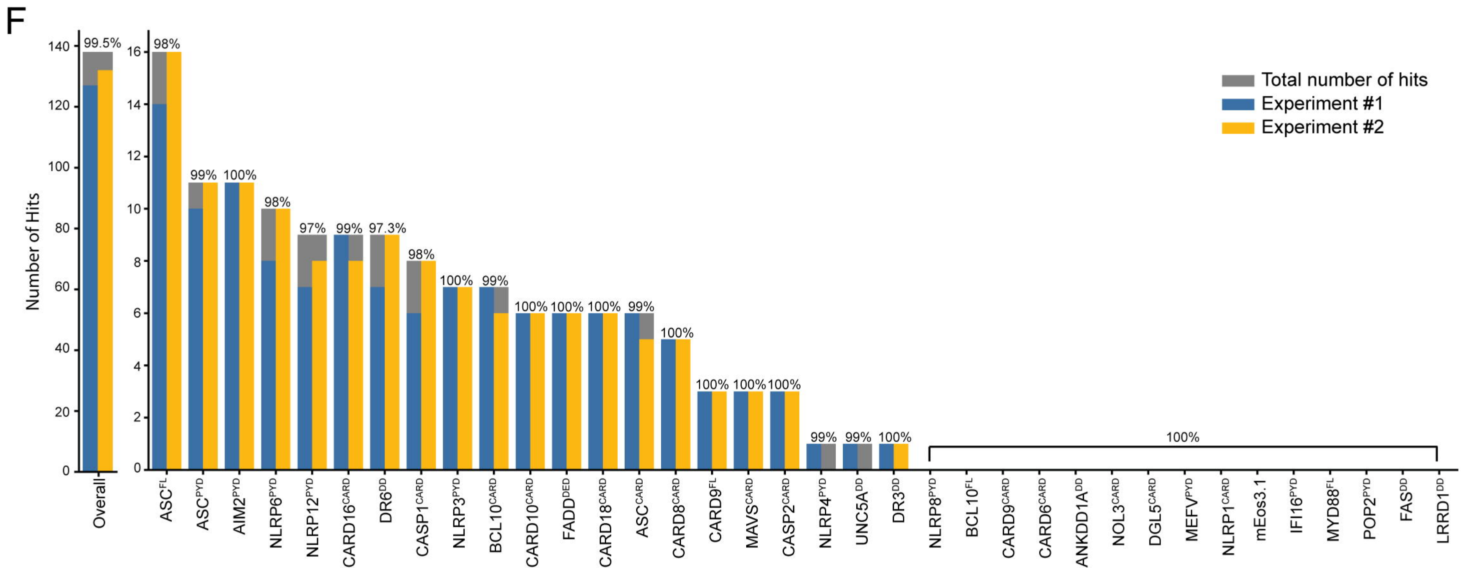

Figure S7

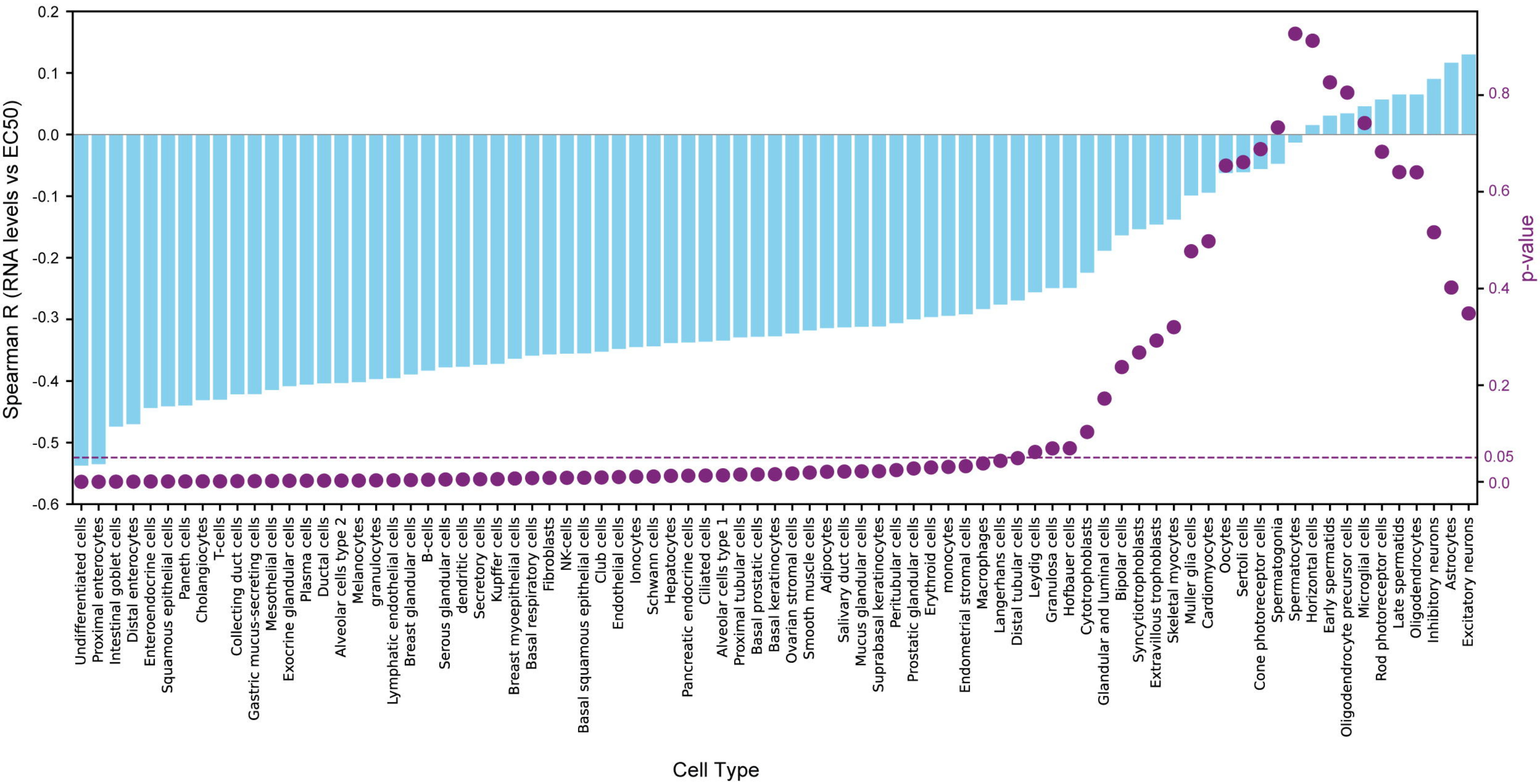

Spearman R (RNA levels vs EC50)
p-value
0.05
Cell Type
Undifferentiated cells
Proximal enterocytes
Intestinal goblet cells
Distal enterocytes
Enteroendocrine cells
Squamous epithelial cells
Paneth cells
Cholangiocytes
T-cells
Collecting duct cells
Gastric mucus-secreting cells
Mesothelial cells
Exocrine glandular cells
Plasma cells
Ductal cells
Alveolar cells type 2
Melanocytes
granulocytes
Lymphatic endothelial cells
Breast glandular cells
B-cells
Serous glandular cells
dendritic cells
Secretory cells
Kupffer cells
Breast myoepithelial cells
Basal respiratory cells
Fibroblasts
NK-cells
Basal squamous epithelial cells
Club cells
Endothelial cells
Ionocytes
Schwann cells
Hepatocytes
Pancreatic endocrine cells
Ciliated cells
Alveolar cells type 1
Proximal tubular cells
Basal prostatic cells
Basal keratinocytes
Ovarian stromal cells
Smooth muscle cells
Adipocytes
Salivary duct cells
Mucus glandular cells
Suprabasal keratinocytes
Peritubular cells
Prostatic glandular cells
Erythroid cells
monocytes
Endometrial stromal cells
Macrophages
Langerhans cells
Distal tubular cells
Leydig cells
Granulosa cells
Hofbauer cells
Cytotrophoblasts
Glandular and luminal cells
Bipolar cells
Syncytiotrophoblasts
Extravillous trophoblasts
Skeletal myocytes
Muller glia cells
Cardiomyocytes
Oocytes
Sertoli cells
Cone photoreceptor cells
Spermatogonia
Spermatocytes
Horizontal cells
Early spermatids
Oligodendrocyte precursor cells
Microglial cells
Rod photoreceptor cells
Late spermatids
Oligodendrocytes
Inhibitory neurons
Astrocytes
Excitatory neurons
