# Supplementary material for: Protein supersaturation powers innate immune signaling
Source: bioRxiv. 2023 Mar 21:2023.03.20.533581. Preprint. [Version 1] doi: 10.1101/2023.03.20.533581 (PMC10055258; doi:10.1101/2023.03.20.533581)

Figure S1

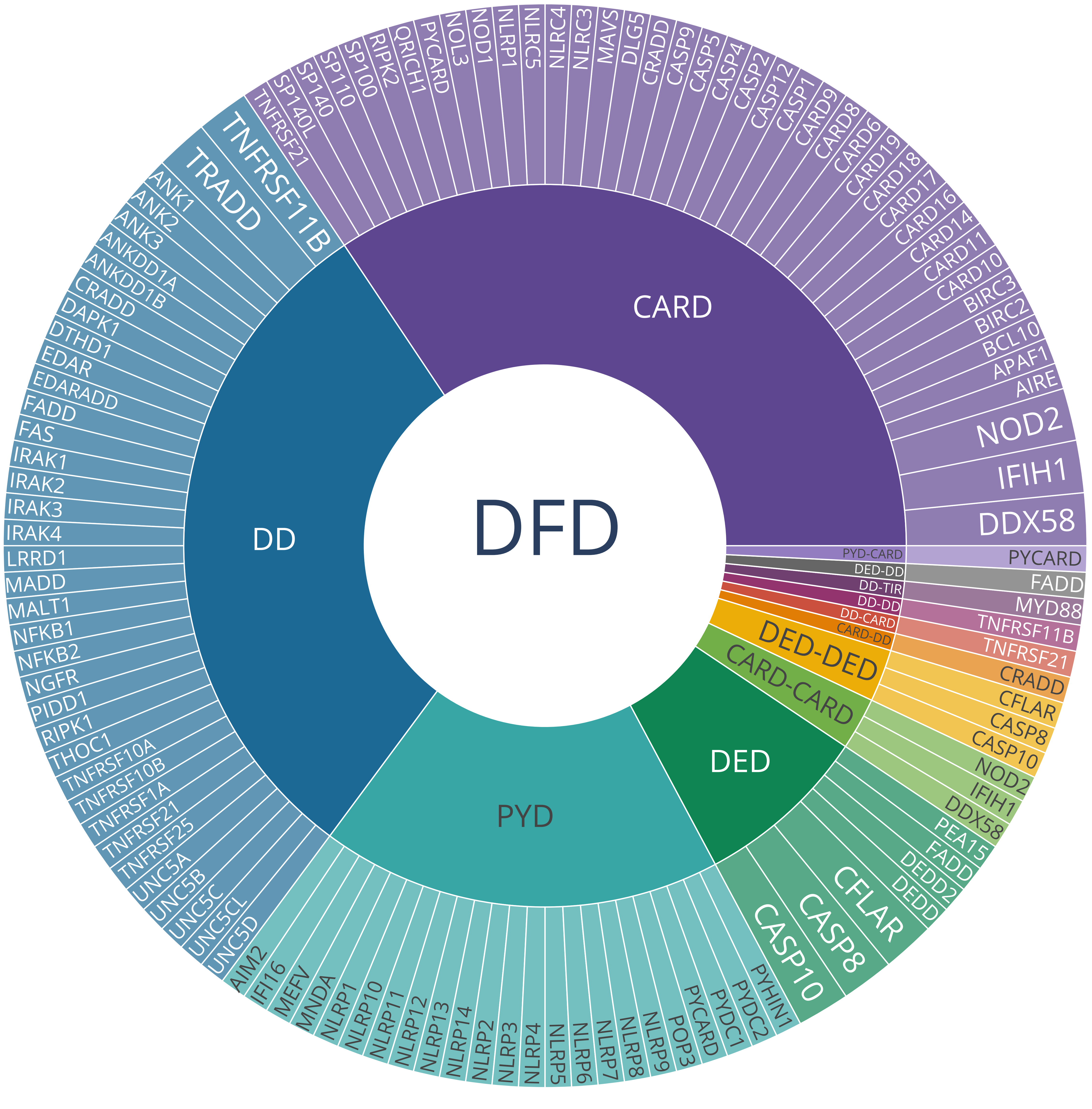

DFD
CARD
DD
PYD
DED
PYD-CARD
DED-DD
DD-TIR
DD-DD
DD-CARD
CARD-DD
DED-DED
CARD-CARD
TNFRSF21
SP140L
SP140
SP110
SP100
RIPK2
QRICH1
PYCARD
NOL3
NOD1
NLRP1
NLRC5
NLRC4
NLRC3
MAVS
DLG5
CRADD
CASP9
CASP5
CASP4
CASP2
CASP12
CASP1
CARD9
CARD8
CARD6
CARD19
CARD18
CARD17
CARD16
CARD14
CARD11
CARD10
BIRC3
BIRC2
BCL10
APAF1
AIRE
NOD2
IFIH1
DDX58
PYCARD
FADD
MYD88
TNFRSF11B
TNFRSF21
CRADD
CFLAR
CASP8
CASP10
NOD2
IFIH1
DDX58
PEA15
FADD
DEDD2
DEDD
CFLAR
CASP8
CASP10
PYHIN1
PYDC2
PYDC1
PYCARD
POP3
NLRP9
NLRP8
NLRP7
NLRP6
NLRP5
NLRP4
NLRP3
NLRP2
NLRP14
NLRP13
NLRP12
NLRP11
NLRP10
NLRP1
MNDA
MEFV
IFI16
AIM2
UNC5D
UNC5CL
UNC5C
UNC5B
UNC5A
TNFRSF25
TNFRSF21
TNFRSF1A
TNFRSF10B
TNFRSF10A
THOC1
RIPK1
PIDD1
NGFR
NFKB2
NFKB1
MALT1
MADD
LRRD1
IRAK4
IRAK3
IRAK2
IRAK1
FAS
FADD
EDARADD
EDAR
DTHD1
DAPK1
CRADD
ANKDD1B
ANKDD1A
ANK3
ANK2
ANK1
TRADD
TNFRSF11B

Figure S2

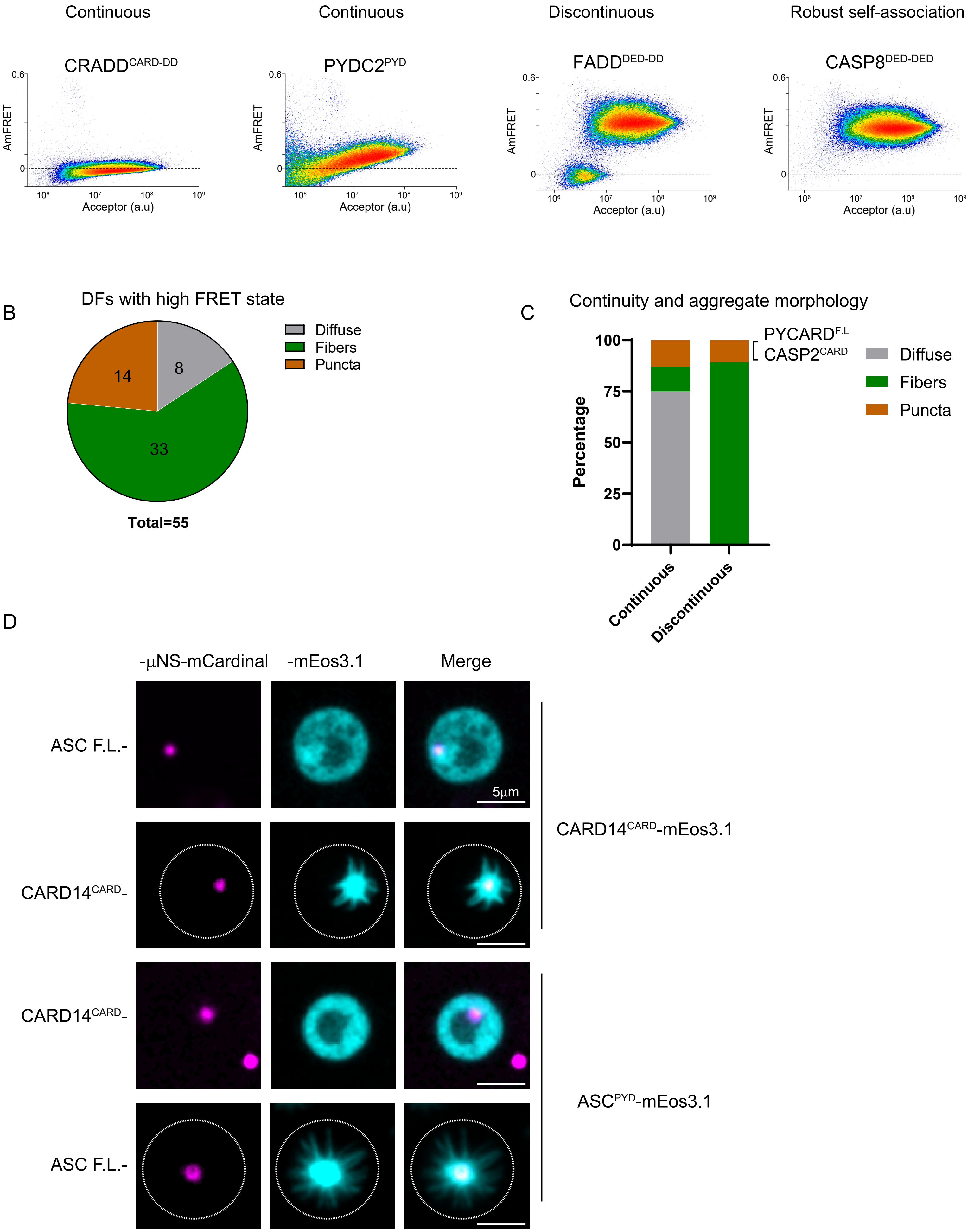

A
Continuous
Continuous
Discontinuous
Robust self-association
CRADD^CARD-DD
PYDC2^PYD
FADD^DED-DD
CASP8^DED-DED
AmFRET
Acceptor (a.u)
0.6
0
10^6
10^7
10^8
10^9
B
DFs with high FRET state
Diffuse
Fibers
Puncta
8
14
33
Total=55
C
Continuity and aggregate morphology
PYCARD^F.L
CASP2^CARD
Percentage
100
75
50
25
0
Continuous
Discontinuous
D
-µNS-mCardinal
-mEos3.1
Merge
ASC F.L.-
CARD14^CARD-
CARD14^CARD-
ASC F.L.-
5µm
CARD14^CARD-mEos3.1
ASC^PYD-mEos3.1

Figure S3

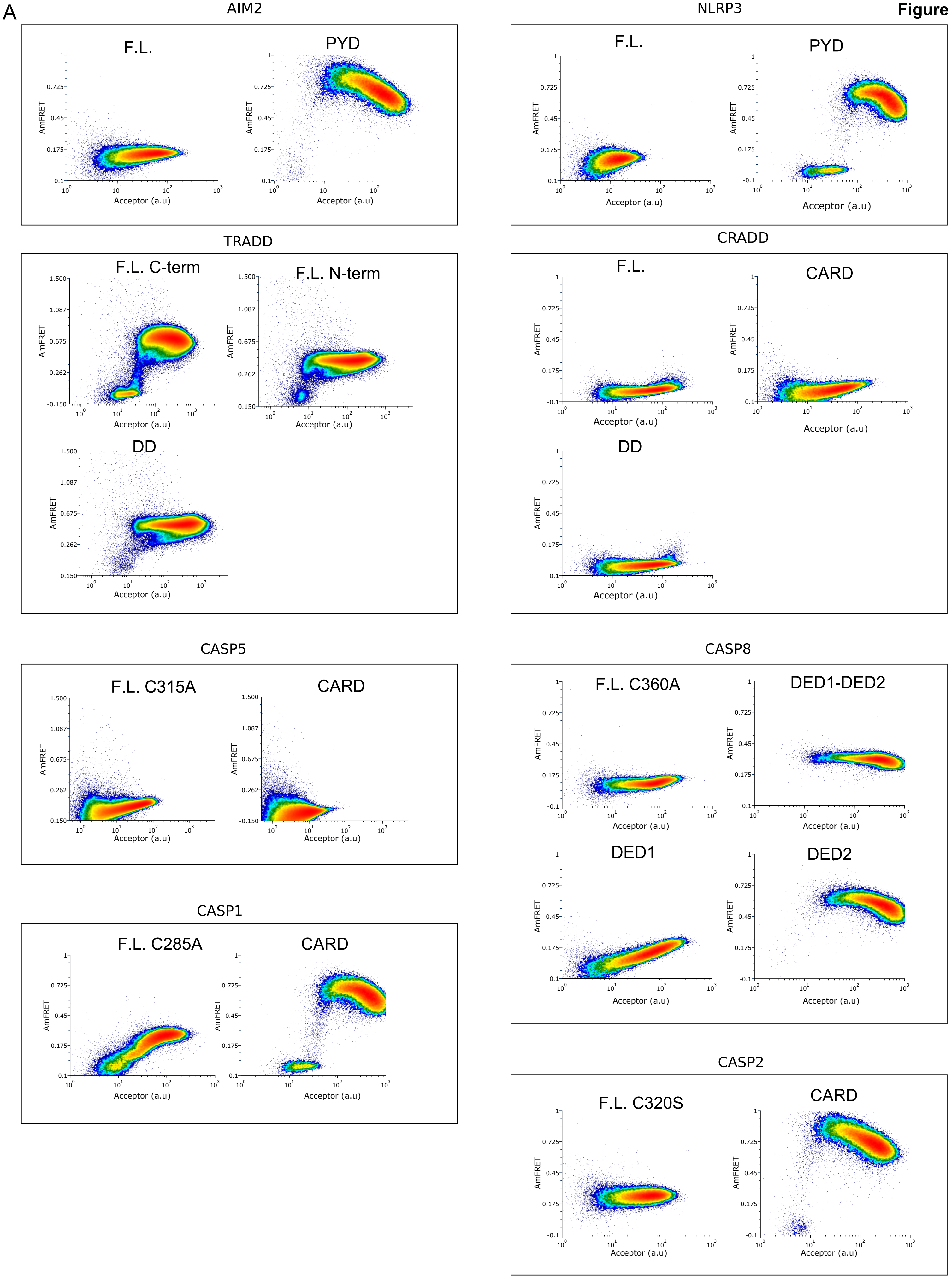
A
AIM2
F.L.
PYD
NLRP3
F.L.
PYD
TRADD
F.L. C-term
F.L. N-term
DD
CRADD
F.L.
CARD
DD
CASP5
F.L. C315A
CARD
CASP8
F.L. C360A
DED1-DED2
DED1
DED2
CASP1
F.L. C285A
CARD
CASP2
F.L. C320S
CARD
AmFRET
Acceptor (a.u)

Figure S4

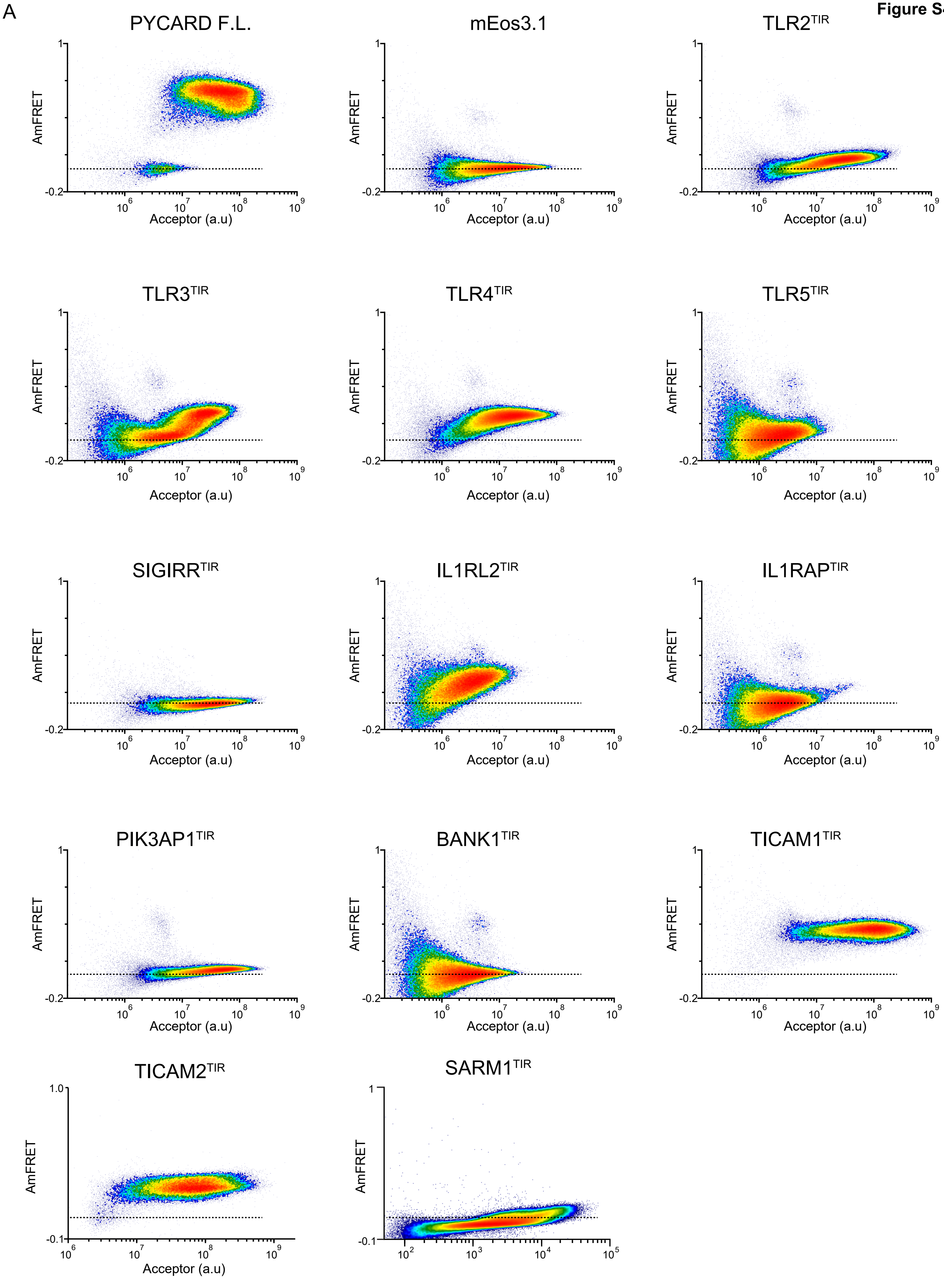
A
PYCARD F.L.
mEos3.1
TLR2TIR
TLR3TIR
TLR4TIR
TLR5TIR
SIGIRRTIR
IL1RL2TIR
IL1RAPTIR
PIK3AP1TIR
BANK1TIR
TICAM1TIR
TICAM2TIR
SARM1TIR
AmFRET
Acceptor (a.u)
1
-0.2
1.0
-0.1
10^6
10^7
10^8
10^9
10^2
10^3
10^4
10^5

Figure S5

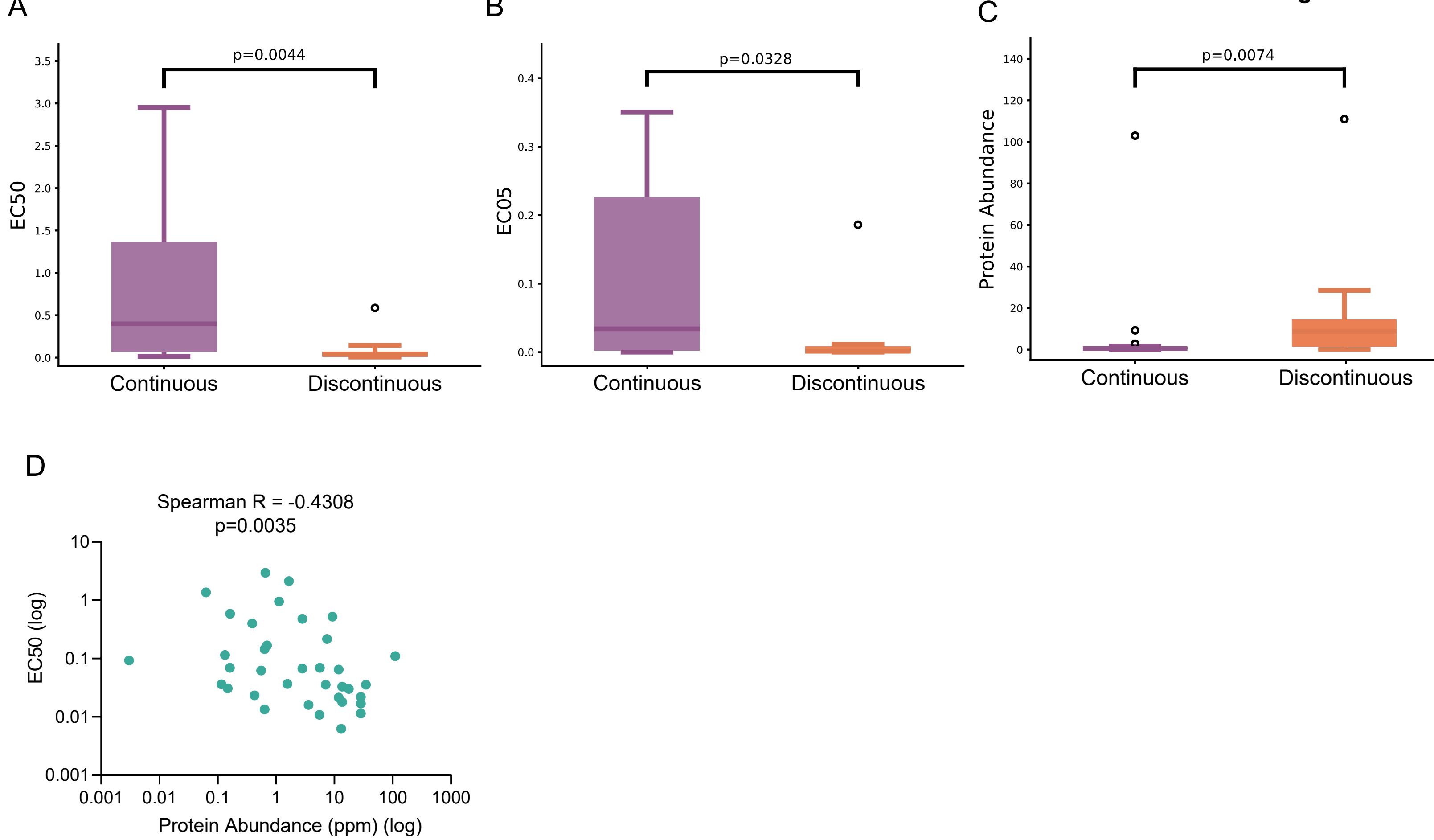

A
p=0.0044
EC50
3.5
3.0
2.5
2.0
1.5
1.0
0.5
0.0
Continuous
Discontinuous
B
p=0.0328
EC05
0.4
0.3
0.2
0.1
0.0
Continuous
Discontinuous
C
p=0.0074
Protein Abundance
140
120
100
80
60
40
20
0
Continuous
Discontinuous
D
Spearman R = -0.4308
p=0.0035
EC50 (log)
10
1
0.1
0.01
0.001
0.001
0.01
0.1
1
10
100
1000
Protein Abundance (ppm) (log)

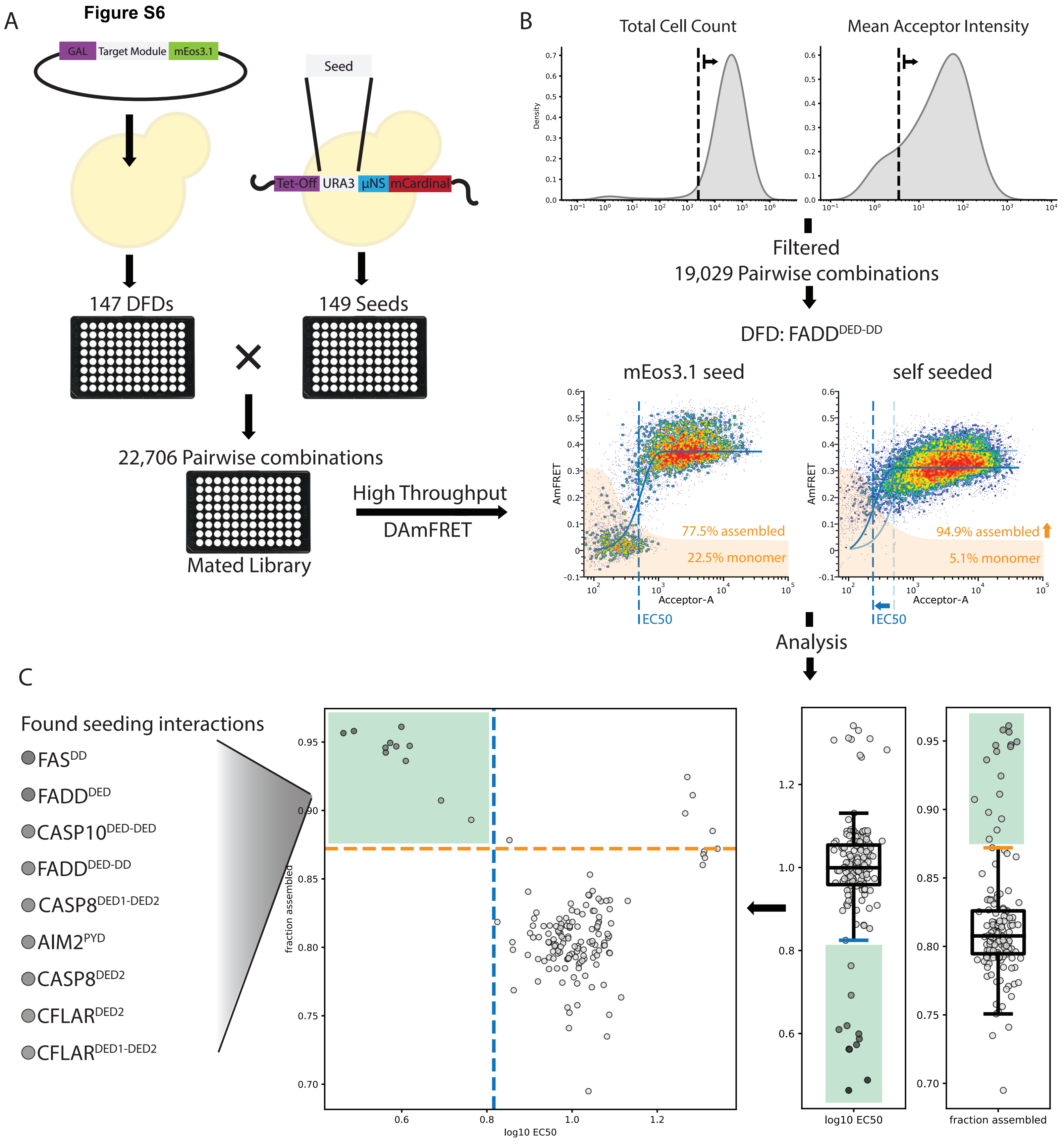
Figure S6
A
GAL
Target Module
mEos3.1
Seed
Tet-Off
URA3
µNS
mCardinal
147 DFDs
149 Seeds
22,706 Pairwise combinations
Mated Library
High Throughput
DAmFRET
B
Total Cell Count
Mean Acceptor Intensity
Density
Filtered
19,029 Pairwise combinations
DFD: FADDDED-DD
mEos3.1 seed
self seeded
AmFRET
Acceptor-A
77.5% assembled
22.5% monomer
94.9% assembled
5.1% monomer
EC50
Analysis
C
Found seeding interactions
FASDD
FADDDED
CASP10DED-DED
FADDDED-DD
CASP8DED1-DED2
AIM2PYD
CASP8DED2
CFLARDED2
CFLARDED1-DED2
fraction assembled
log10 EC50

Figure S7

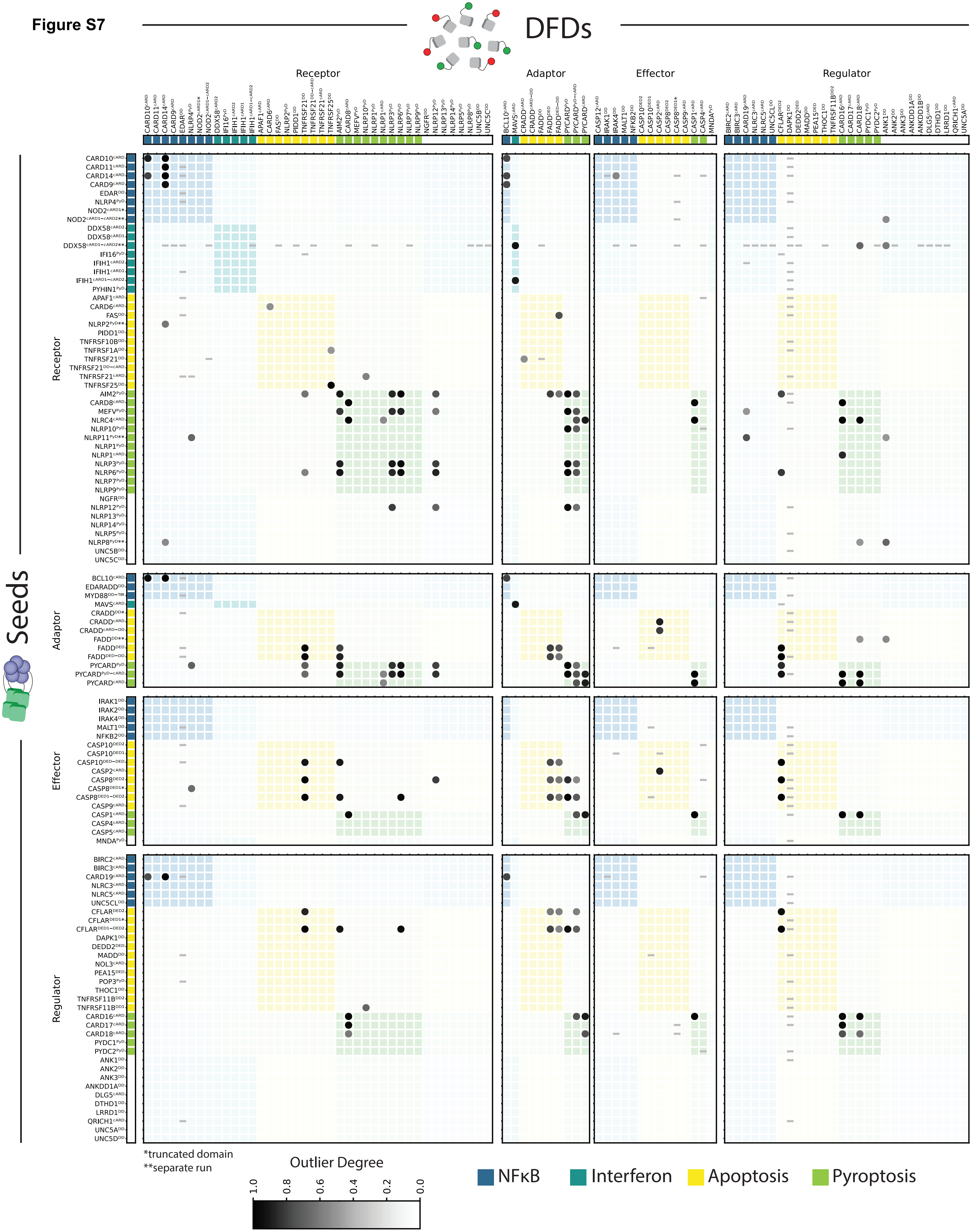
DFDs
Receptor
Adaptor
Effector
Regulator
Seeds
*truncated domain
**separate run
Outlier Degree
1.0
0.8
0.6
0.4
0.2
0.0
NFκB
Interferon
Apoptosis
Pyroptosis

Figure S8

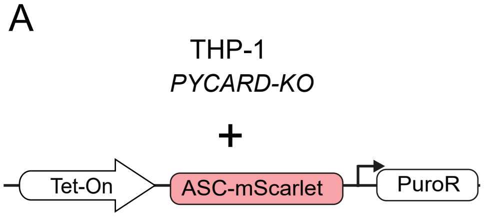

A
THP-1
PYCARD-KO
+
Tet-On
ASC-mScarlet
PuroR

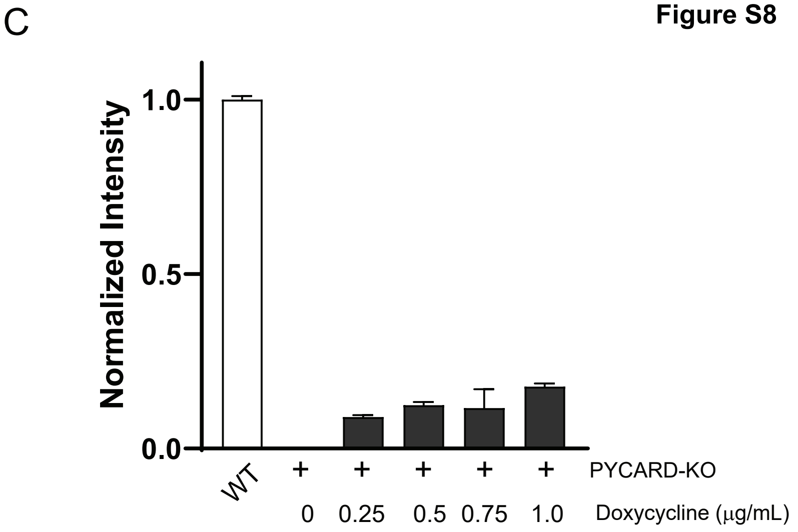

C
Normalized Intensity
1.0
0.5
0.0
WT
PYCARD-KO
0
0.25
0.5
0.75
1.0
Doxycycline (μg/mL)

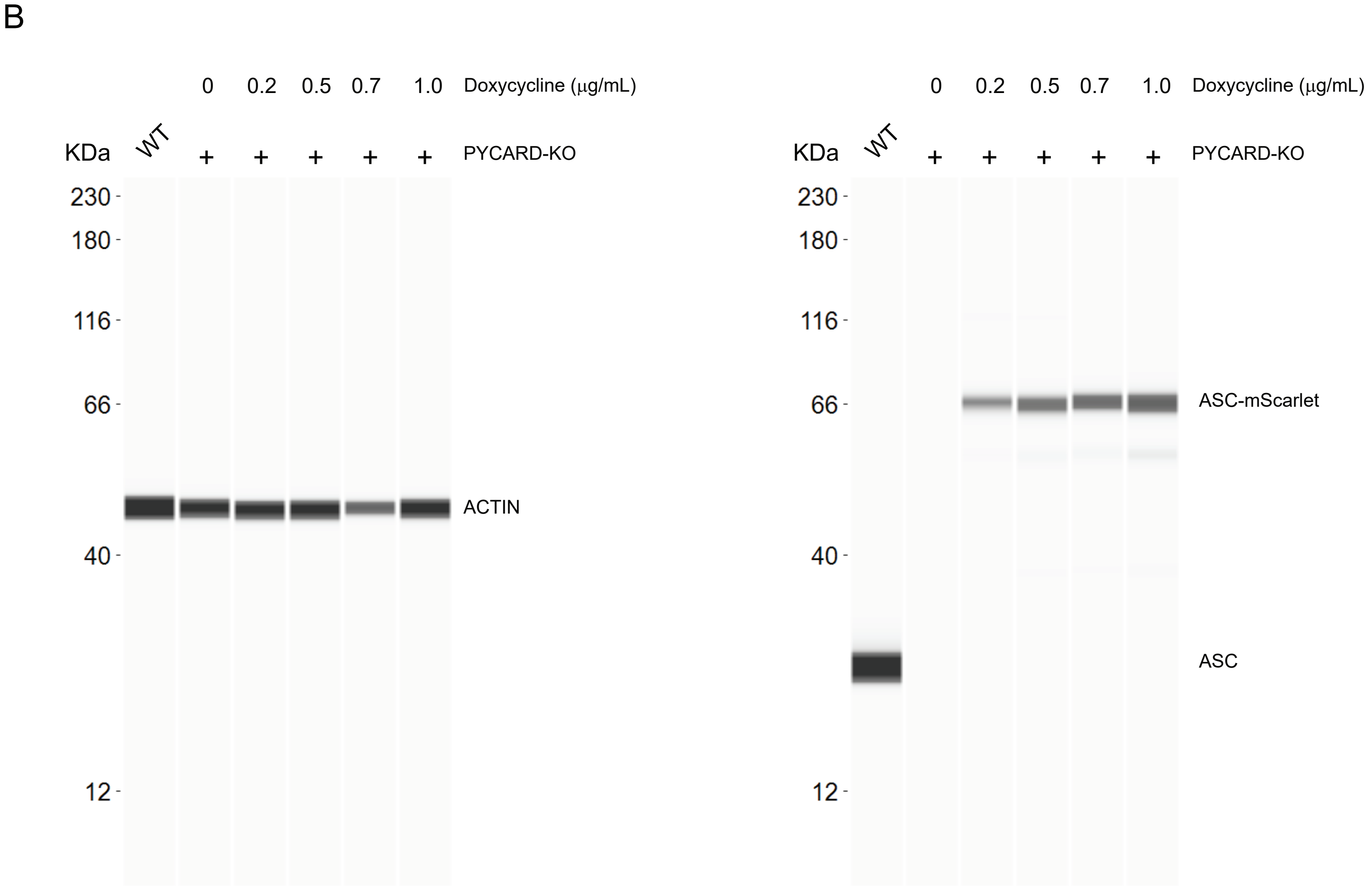

B
0
0.2
0.5
0.7
1.0
Doxycycline (μg/mL)
KDa
WT
PYCARD-KO
230
180
116
66
40
12
ACTIN
ASC-mScarlet
ASC

A

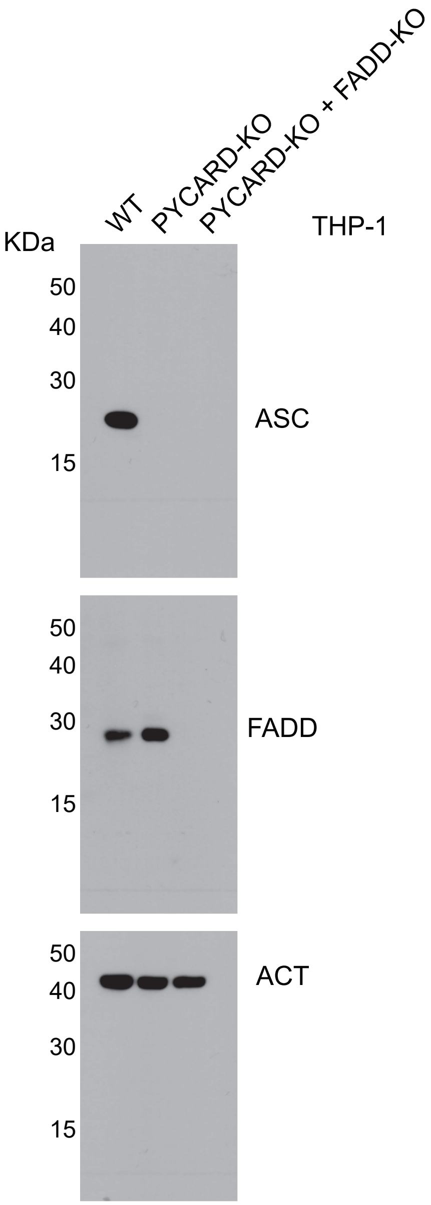

Supplement: Supplementary file 1 [file media-1.pdf]
