# Supplementary material for: Protein supersaturation powers innate immune signaling
Source: bioRxiv. 2023 Mar 21:2023.03.20.533581. Preprint. [Version 1] doi: 10.1101/2023.03.20.533581 (PMC10055258; doi:10.1101/2023.03.20.533581)
Supplement: Supplementary file 2 [file media-2.pdf]

expression_clone	gene_name	subfamily	uniprot	region_of_gene	pathway	protein_function	continuity	AR	CV	image_result	protein_abundance (ppm)	EC50	EC50_err	EC05	EC05_err
rhx0644	APAF1	CARD	O14727	2-100	apoptosis	adaptor	continuous (all low FRET)	1.1	27.9	D	2.79	nd	nd	nd	nd
rhx0653	FAS	DD	P25445	221-335	apoptosis	receptor	discontinuous	1.2	73.2	F	5.65	0.069277	0.0023714	0.011429153	0.0012935
rhx0660	RIPK1	DD	Q13546	576-671	necroptosis	adaptor	continuous	1.2	58.8	P	5.48				
rhx0661	RIPK2	CARD	O43353	431-530	NFKB	adaptor	continuous	1.1	46.8	P	3.13				
rhx0927	PYCARD	PYD-CARD	Q9ULZ3	FL	pyroptosis	adaptor	discontinuous	1.1	97.7	P	28.5	0.011411	0.0006554	0.001405914	0.0002729
rhx0928	NLRP3	PYD	Q96P20	2-95	pyroptosis	receptor	discontinuous	1.2	72.9	F	0.161	0.069372	0.0026428	0.003676004	0.000511
rhx0988	PYCARD	PYD	Q9ULZ3	2-94	pyroptosis	adaptor	discontinuous	1.2	60.7	F	28.5	0.021932	0.0010808	0.000807005	0.0001631
rhx0989	PYCARD	CARD	Q9ULZ3	110-195	pyroptosis	adaptor	discontinuous	1.2	64.5	F	28.5	0.016919	0.0389909	3.14756E-08	nd
rhx1033	LRRD1	DD	A4D1F6	759-860		regulator	continuous	1.2	40.5	D	0.091	nd	nd	nd	nd
rhx1051	TNFRSF10B	DD	O14763	338-428	apoptosis	receptor	continuous (all low FRET)	1.1	28.1	D	1.69				
rhx1052	AIM2	PYD	O14862	2-92	pyroptosis	receptor	discontinuous	1.3	69.6	F	0.429	0.023432	0.0145394	4.5718E-05	nd
rhx1053	CFLAR	DED1-DED2	O15519	2-181	apoptosis	regulator	all high fret	1.3	77.7	F	0.701				
rhx1054	MEFV	PYD	O15553	2-98	pyroptosis	receptor	discontinuous	1.3	81.8	F	0.163	0.586288	0.0130321	0.185988152	0.0118953
rhx1055	IRAK2	DD	O43187	9-93	NFKB	effector	all high fret	1.3	92.5	P	0.062				
rhx1056	NOL3	CARD	O60936	8-96	apoptosis	regulator	all high fret	1.1	43.4	P	12.3				
rhx1058	TNFRSF21	DD-CARD	O75509	414-655	apoptosis	receptor	continuous (all low FRET)	1.1	34.0	D	0.171	nd	nd	nd	nd
rhx1060	UNC5C	DD	O95185	844-931		receptor	continuous (all low FRET)	1.1	36.7	D	0.099	0.522693	0.6460374	0.363173446	nd
rhx1062	NGFR	DD	P08138	340-425		receptor	continuous (all low FRET)	1.1	34.5	D	2.41	nd	nd	nd	nd
rhx1063	ANK1	DD	P16157	1402-1487		regulator	continuous (all low FRET)	1.1	36.4	D	58.7	1.440413	0.4736286	0.444934753	nd
rhx1064	TNFRSF1A	DD	P19438	355-441	apoptosis	receptor	all high fret	1.3	79.8	F	0.369				
rhx1066	CASP1	CARD	P29466	2-91	pyroptosis	effector	discontinuous	1.2	54.3	F	17.7	0.03003	0.001796	0.001154893	0.0002717
rhx1067	MNDA	PYD	P41218	2-93		effector	continuous (all low FRET)	1.1	34.0	D	44.3	2.670571	0.4206506	0.51149621	0.1305746
rhx1068	CASP2	CARD	P42575	32-121	apoptosis	effector	discontinuous	1.3	67.6	P	3.61	0.015988	0.0010877	0.000211288	6.249E-05
rhx1069	CASP4	CARD	P49662	2-91	pyroptosis	effector	continuous (all low FRET)	1.1	27.6	P	3.23	nd	nd	nd	nd
rhx1070	IRAK1	DD	P51617	23-102	NFKB	effector	continuous	1.2	94.1	P	5.58	0.010872	0.0092306	1.29867E-05	nd
rhx1071	CASP5	CARD	P51878	56-148	pyroptosis	effector	continuous (all low FRET)	1.1	14.6	D	0.581				
rhx1072	DAPK1	DD	P53355	1305-1398	apoptosis	regulator	continuous (all low FRET)	1.2	48.8	F	1.71	nd	nd	nd	nd
rhx1073b	CARD18	CARD	P57730	FL	pyroptosis	regulator	continuous	1.2	49.3	F	0.656	2.952491	5.5238656	0.02380993	0.0761474
rhx1074	NLRP6	PYD	P59044	14-109	pyroptosis	receptor	discontinuous	1.2	65.1	F	0.148	0.030793	0.0199735	7.25538E-05	nd
rhx1075b	NLRP11	PYD	P59045	2-92	pyroptosis	receptor	continuous (all low FRET)	1.1	33.9	D	0.031				
rhx1076	NLRP12	PYD	P59046	3-98		receptor	continuous (all low FRET)	1.1	33.5	D	0.048	1.248323	0.1281376	0.350602029	0.0534896
rhx1077	NLRP5	PYD	P59047	59-151		receptor	continuous (all low FRET)	1.1	36.2	D	0.013	1.71183	0.3963441	0.226587784	0.0857261
rhx1078	CRADD	CARD-DD	P78560	2-199	apoptosis	adaptor	continuous (all low FRET)	1.1	36.1	D	3.02	nd	nd	nd	nd
rhx1097	NFKB2	DD	Q00653	772-851	NFKB	effector	n.d.	1.1	7.5	D	17.2	nd	nd	nd	nd
rhx1098	ANK2	DD	Q01484	3568-3653		regulator	continuous (all low FRET)	1.1	33.0	D	4.66	nd	nd	nd	nd
rhx1099	ANK3	DD	Q12955	4089-4174		regulator	continuous (all low FRET)	1.1	36.7	D	3.66	nd	nd	nd	nd
rhx1100	BIRC3	CARD	Q13489	441-529	NFKB	regulator	continuous (all low FRET)	1.1	34.7	D	0.472	nd	nd	nd	nd
rhx1101	BIRC2	CARD	Q13490	453-543	NFKB	regulator	continuous (all low FRET)	1.1	35.3	D	1.32	nd	nd	nd	nd
rhx1102b	PEA15	DED	Q15121	FL	apoptosis	regulator	continuous (all low FRET)	1.1	34.3	D	103	2.030306	0.3231547	0.308949117	0.0740248
rhx1103	IFI16	PYD	Q16666	2-93	interferon	receptor	discontinuous	1.2	51.9	F	111	0.110167	0.0031405	0.008587647	0.0007091
rhx1105	ANKDD1A	DD	Q495B1	411-504		regulator	continuous	1.2	45.0	F	0.003	0.092671	0.0086207	0.002353901	0.0006644
rhx1106	PYDC2	PYD	Q56P42	2-97	pyroptosis	regulator	continuous	1.2	43.4	P	0.133	0.115222	0.1719827	0.000684613	nd
rhx1107	CARD16	CARD	Q5EG05	2-91	pyroptosis	regulator	discontinuous	1.2	57.8	F	7.13	0.035674	0.0611639	1.09629E-06	nd
rhx1108	CARD17	CARD	Q5XLA6	2-91	pyroptosis	regulator	continuous (all low FRET)	1.1	37.0	D	0.644	2.489146	2.2942659	0.355774248	nd
rhx1109	PYHIN1	PYD	Q6K0P9	2-93	interferon	receptor	all high fret	1.2	58.8	F	6.29				
rhx1113	UNC5D	DD	Q6UXZ4	853-942		regulator	n.d.	1.1	11.6	n.d	0.092				
rhx1114	DTHD1	DD	Q6ZMT9	678-764		regulator	continuous (all low FRET)	1.1	31.8	D	0.013	nd	nd	nd	nd
rhx1115	UNC5A	DD	Q6ZN44	755-842		regulator	continuous (all low FRET)	1.2	51.6	F	0.234	nd	nd	nd	nd
rhx1116	NLRP9	PYD	Q7RTR0	2-95	pyroptosis	receptor	continuous (all low FRET)	1.1	36.7	D	0.021	nd	nd	nd	nd
rhx1117	MAVS	CARD	Q7Z434	2-97	interferon	adaptor	discontinuous	1.2	55.9	F	34.9	0.035502	0.0659459	3.25009E-07	nd
rhx1118	NLRP14	PYD	Q86W24	6-104		receptor	n.d.	1.1	32.5	D	0.01	nd	nd	nd	nd

rhx1119	NLRP13	PYD	Q86W25	9-110		receptor	continuous (all low FRET)	1.1	34.4	D	0.518	nd	nd	nd	nd
rhx1120	NLRP10	PYD	Q86W26	2-100	pyroptosis	receptor	continuous (all low FRET)	1.1	38.3	D	0.04	0.88416	0.5055932	0.235968983	nd
rhx1121	NLRP8	PYD	Q86W28	31-130		receptor	continuous	1.2	65.7	P	0	0.188682	0.0086344	0.034106456	0.0044879
rhx1122	UNC5CL	DD	Q8IV45	408-493	NFKB	regulator	continuous	1.2	71.0	F	0.392	0.398557	0.0095301	0.102042165	0.0076912
rhx1123	UNC5B	DD	Q8IZJ1	858-945		receptor	continuous (all low FRET)	1.1	35.1	D	0.885	nd	nd	nd	nd
rhx1124	DLG5	CARD	Q8TDM6	2-90		regulator	continuous	1.1	43.2	P	1.67	2.131524	0.0646661	0.265069487	0.0132348
rhx1125	EDARADD	DD	Q8WWZ3	119-199	NFKB	adaptor	n.d.	1.1	4.5	n.d	0.983				
rhx1126	NLRP7	PYD	Q8WX94	2-96	pyroptosis	receptor	continuous (all low FRET)	1.1	34.1	D	1.95	nd	nd	nd	nd
rhx1127	PYDC1	PYD	Q8WXC3	2-89	pyroptosis	regulator	continuous (all low FRET)	1.1	35.3	D	1.52	nd	nd	nd	nd
rhx1128	DEDD2	DED	Q8WXF8	14-109	apoptosis	regulator	continuous	1.1	88.8	P	0.554	0.062444	0.0067307	0.000332539	0.0001376
rhx1129	CASP10	DED-DED	Q92851	13-200	apoptosis	effector	all high fret	1.2	55.5	F	2.83				
rhx1130	TNFRSF25	DD	Q93038	331-413	apoptosis	receptor	discontinuous	1.2	62.2	F	0.637	0.145577	0.0033245	0.010660595	0.0009199
rhx1131	THOC1	DD	Q96FV9	568-652	apoptosis	regulator	continuous (all low FRET)	1.1	36.5	D	10.7	0.46424	10.635254	2.25625E-05	nd
rhx1132	CARD19	CARD	Q96LW7	2-105	NFKB	regulator	continuous (all low FRET)	1.1	37.2	D	5	nd	nd	nd	nd
rhx1133	NLRP4	PYD	Q96MN2	2-95	NFKB	receptor	continuous (all low FRET)	1.1	39.4	D	0.285	nd	nd	nd	nd
rhx1134	CARD10	CARD	Q9BWT7	22-115	NFKB	receptor	continuous	1.4	99.0	F	0.116	0.036149	0.0048957	2.77212E-05	1.533E-05
rhx1135	CARD6	CARD	Q9BX69	7-94	apoptosis	receptor	continuous	1.2	73.4	F	0.117	0.036846	0.0015024	0.000629114	0.0001028
rhx1136	NLRP2	PYD	Q9NX02	2-104	apoptosis	receptor	continuous (all low FRET)	1.1	34.6	D	19.5	2.146323	2.7762016	0.056848737	0.1039871
rhx1137	IFIH1	CARD-CARD	Q9BYX4	2-200	interferon	receptor	continuous (all low FRET)	1.1	45.5	P	7.5	0.252814	0.1452971	0.012268696	28962263
rhx1138	NLRP1	CARD	Q9C000	1376-1463	pyroptosis	receptor	continuous	1.1	34.9	D	0.063	1.363606	0.3206906	0.042047664	0.0158344
rhx1139	NLRP1	PYD	Q9C000	2-93	pyroptosis	receptor	continuous (all low FRET)	1.1	33.3	D	0.063	0.530785	2.962448	0.001397446	nd
rhx1140	CARD9	CARD	Q9H257	2-98	NFKB	receptor	continuous	1.2	59.9	F	1.13	0.948502	0.0175558	0.283140532	0.0149305
rhx1141	PIDD1	DD	Q9HB75	788-873	apoptosis	receptor	continuous (all low FRET)	1.1	35.5	D	0.169	nd	nd	nd	nd
rhx1142	NLRC4	CARD	Q9NPP4	2-89	pyroptosis	receptor	all high fret	1.3	69.8	F	0.248				
rhx1143	IRAK4	DD	Q9NWZ3	12-106	NFKB	effector	continuous (all low FRET)	1.1	39.3	D	9.1	nd	nd	nd	nd
rhx1144	CARD14	CARD	Q9BXL6	9-107	NFKB	receptor	continuous	1.3	76.9	F	0.639	0.013489	0.002949	5.63012E-06	5.926E-06
rhx1145	EDAR	DD	Q9UNE0	354-431	NFKB	receptor	continuous (all low FRET)	1.1	35.8	D	0.229				
rhx1146	CARD8	CARD	Q9Y2G2	339-431	pyroptosis	receptor	discontinuous	1.2	65.0	F	1.57	0.036588	0.0003877	0.001263368	5.068E-05
rhx1174	NLRC5	CARD	Q86WI3	2-101	NFKB	regulator	continuous (all low FRET)	1.1	34.3	D	0.046	0.171297	1.5060503	0.000150247	nd
rhx1176	NLRC3	CARD	Q7RTR2	19-114	NFKB	regulator	continuous (all low FRET)	1.1	27.0	D	0.1	nd	nd	nd	nd
rhx1211	NOD2	CARD1	Q9HC29	25-123	NFKB	receptor	continuous (all low FRET)	1.2	37.4	P	0.023	nd	nd	nd	nd
rhx1212	NOD2	CARD2	Q9HC29	130-220	NFKB	receptor	continuous (all low FRET)	1.1	3.8	D	0.023				
rhx1213	CASP8	DED1	Q14790	2-82	apoptosis	effector	continuous	1.1	14.3	D	11.9	0.021522	0.0167325	0.000128167	nd
rhx1214	CASP8	DED2	Q14790	95-190	apoptosis	effector	discontinuous	1.3	66.9	F	11.9	0.064665	0.0015271	0.002308758	0.0001702
rhx1215	TNFRSF11B	DD1	O00300	198-278	apoptosis	regulator	n.d.	1.1	10.2	n.d	0.193				
rhx1216	TNFRSF11B	DD2	O00300	279-367	apoptosis	regulator	continuous (all low FRET)	1.1	32.3	D	0.193	nd	nd	nd	nd
rhx1217	CFLAR	DED1	O15519	2-75	apoptosis	regulator	continuous	1.1	3.8	n.d	0.701				
rhx1218	CFLAR	DED2	O15519	87-181	apoptosis	regulator	continuous	1.1	24.2	D	0.701	0.168181	0.0101115	0.021221596	0.0030154
rhx1219	TNFRSF21	DD	O75509	414-497	apoptosis	receptor	n.d.	1.2	15.8	D	0.171	nd	nd	nd	nd
rhx1220	TNFRSF21	CARD	O75509	576-655	apoptosis	receptor	continuous (all low FRET)	1.1	35.7	D	0.171	nd	nd	nd	nd
rhx1221	DDX58	CARD1	O95786	2-93	interferon	receptor	n.d.	1.1	5.9	n.d	9.73				
rhx1222	DDX58	CARD2	O95786	94-199	interferon	receptor	continuous (all low FRET)	1.1	32.6	D	9.73	nd	nd	nd	nd
rhx1223	CRADD	CARD	P78560	2-95	apoptosis	adaptor	continuous (all low FRET)	1.1	33.6	D	3.02	nd	nd	nd	nd
rhx1224	CRADD	DD	P78560	116-199	apoptosis	adaptor	continuous	1.1	11.9	D	3.02				
rhx1225	CASP10	DED1	Q92851	13-105	apoptosis	effector	continuous	1.1	29.8	D	2.83	0.067418	0.0121613	0.004562742	0.0022382
rhx1226	CASP10	DED2	Q92851	109-200	apoptosis	effector	continuous	1.1	32.4	D	2.83	0.481409	0.097531	0.308614872	nd
rhx1227	IFIH1	CARD1	Q9BYX4	2-103	interferon	receptor	continuous	1.1	34.8	P	7.5	0.21556	0.4259633	0.020705469	nd
rhx1228	IFIH1	CARD2	Q9BYX4	107-200	interferon	receptor	continuous (all low FRET)	1.1	35.6	D	7.5	3.467906	1.9518002	0.351857131	0.246961
rhx1229	QRICH1	CARD	Q2TAL8	2-84		regulator	continuous	1.1	88.7	P	9.31	0.522401	0.0170639	0.100513748	0.0077675
rhx1311	MYD88	DD-TIR	Q99836	FL	NFKB	adaptor	all high fret	1.2	98.0	F	13.3				
rhx1312	FADD	DED-DD	Q13158	FL	apoptosis	adaptor	discontinuous	1.3	81.8	F	13.6	0.03302	0.0491929	7.73586E-07	nd
rhx1313	CASP8	DED1-DED2	Q14790	2-190	apoptosis	effector	all high fret	1.2	57.2	F	11.9				
rhx1368	POP3	PYD	W6CW81	FL	apoptosis	regulator	n.d.	1.1	21.1	n.d	n.a.				

rhx1884	DDX58	CARD1-CARD2	O95786	1-200	interferon	receptor	continuous (all low FRET)	1.1	28.6	D	9.73				
rhx1899	FADD	DED	Q13158	2-90	apoptosis	adaptor	discontinuous	1.3	91.2	F	13.6	0.017984	0.0197648	3.42241E-08	3.815E-07
rhx2115	MALT1	DD	Q9UDY8	3-130	NFKB	effector	continuous (all low FRET)	1.1	34.4	D	6.17	nd	nd	nd	nd
rhx2116	MADD	DD	Q8WXG6	1312-1425	apoptosis	regulator	continuous (all low FRET)	1.1	33.3	D	4.41	0.449418	1.4617039	0.007598945	nd
rhx2117	CASP9	CARD	P55211	2-93	apoptosis	effector	continuous (all low FRET)	1.1	33.4	D	3.47	nd	nd	nd	nd
rhx2118b	TRADD	DD	Q15628	147-312		adaptor	continuous	1.2	63.9	F	4.71				
rhx2232	CARD11	CARD	Q9BXL7	1-110	NFKB	receptor	n.d.	1.2	40.8	F	0.659	nd	nd	nd	nd
rhx2409	BCL10	CARD	O95999	1-115	NFKB	adaptor	discontinuous	1.2	78.1	F	13.1	0.006242	0.0004013	0.000108698	2.938E-05
rhx2919	NOD2	CARD1-CARD2	Q9HC29	2-220	NFKB	receptor	continuous (all low FRET)	1.1	35.2	D	0.023	0.737566	9.3941514	0.00026231	nd
rhx2932	ANKDD1B	DD	A6NHY2	426-516		regulator	continuous	1.1	36.5	D	0.001				
rhx2933	TNFRSF10A	DD	O00220	364-454	apoptosis	receptor	n.d.	1.1	3.7	D	0.499				
rhx2934	TNFRSF11B	DD1-DD2	O00300	198-367	apoptosis	regulator	continuous (all low FRET)	1.1	27.7	D	0.193				
rhx2935	DEDD	DED	O75618	14-108	apoptosis	adaptor	all high fret	1.2	95.3	P	0.715				
rhx2937	CASP12	CARD	Q6UXS9	2-97	NFKB	effector	continuous (all low FRET)	1.2	9.4	D	n.a.				
rhx2938	IRAK3	DD	Q9Y616	18-105	NFKB	regulator	n.d.	1.1	9.7	n.d	0.417				
rhx3077	FADD	DD	Q13158	97-208	apoptosis	adaptor	continuous (all low FRET)	1.1	33.9	D	13.6	2.942257	2.386841	0.220561446	0.2209993
rhx3078	NOD1	CARD	Q9Y239	2-110		receptor	continuous (all low FRET)	1.1	36.7	D	0.34				
rhx3079	TRADD	DD	Q15628			adaptor	discontinuous	1.5	95.6	F	4.71				
rhx3081	NFKB1	DD	P19838	802-896		effector	continuous (all low FRET)	1.1	34.2	D	22.4				
rhx4763	AIRE	CARD	O43918	2-109			all high fret	n.d	n.d	n.d	0				
rhx4764	SP100	CARD	P23497	2-165			all high fret	n.d	n.d	n.d	21.7				
rhx4765	SP110	CARD	Q9HB58	2-140			continuous	n.d	n.d	n.d	5.14				
rhx4766	SP140	CARD	Q13342	2-184			continuous	n.d	n.d	n.d	0.88				
rhx4767	SP140L	CARD	Q9H930	2-165			all high fret	n.d	n.d	n.d	2.21				
